# The transcriptome, extracellular proteome and active secretome of agroinfiltrated *Nicotiana benthamiana* uncover a large, diverse protease repertoire

**DOI:** 10.1111/pbi.12852

**Published:** 2017-12-17

**Authors:** Friederike Grosse‐Holz, Steven Kelly, Svenja Blaskowski, Farnusch Kaschani, Markus Kaiser, Renier A.L. van der Hoorn

**Affiliations:** ^1^ Plant Chemetics Laboratory Department of Plant Sciences University of Oxford Oxford UK; ^2^ Department of Plant Sciences University of Oxford Oxford UK; ^3^ Chemische Biologie Zentrum für Medizinische Biotechnologie Fakultät für Biologie Universität Duisburg‐Essen Essen Germany

**Keywords:** activity‐based protein profiling, *Agrobacterium tumefaciens*, chlorosis, plant protease annotation, post‐translational activation, silencing inhibitor p19

## Abstract

Infiltration of disarmed *Agrobacterium tumefaciens* into leaves of *Nicotiana benthamiana* (agroinfiltration) facilitates quick and safe production of antibodies, vaccines, enzymes and metabolites for industrial use (molecular farming). However, yield and purity of proteins produced by agroinfiltration are hampered by unintended proteolysis, restricting industrial viability of the agroinfiltration platform. Proteolysis may be linked to an immune response to agroinfiltration, but understanding of the response to agroinfiltration is limited. To identify the proteases, we studied the transcriptome, extracellular proteome and active secretome of agroinfiltrated leaves over a time course, with and without the P19 silencing inhibitor. Remarkably, the P19 expression had little effect on the leaf transcriptome and no effect on the extracellular proteome. 25% of the detected transcripts changed in abundance upon agroinfiltration, associated with a gradual up‐regulation of immunity at the expense of photosynthesis. By contrast, 70% of the extracellular proteins increased in abundance, in many cases associated with increased efficiency of extracellular delivery. We detect a dynamic reprogramming of the proteolytic machinery upon agroinfiltration by detecting transcripts encoding for 975 different proteases and protease homologs. The extracellular proteome contains peptides derived from 196 proteases and protease homologs, and activity‐based proteomics displayed 17 active extracellular Ser and Cys proteases in agroinfiltrated leaves. We discuss unique features of the *N. benthamiana* protease repertoire and highlight abundant extracellular proteases in agroinfiltrated leaves, being targets for reverse genetics. This data set increases our understanding of the plant response to agroinfiltration and indicates ways to improve a key expression platform for both plant science and molecular farming.

## Introduction

Agroinfiltration of *Nicotiana benthamiana* (a relative of tobacco) is widely applied to transiently express proteins, either as biopharmaceutcials, for other industrial use or to study their functions. Agroinfiltration is based on the transient genetic manipulation of leaves by infiltration with disarmed *Agrobacterium tumefaciens* (Agrobacterium) carrying gene(s) of interest on the transfer DNA (T‐DNA) of binary plasmid(s) (Bevan, 1984). Agrobacterium delivers the T‐DNA to the nucleus of its host plant, where genes are expressed within a few days upon agroinfiltration. Co‐expression of several transgenes is simply achieved by mixing Agrobacterium cultures delivering these different transgenes before agroinfiltration. Co‐expression with silencing inhibitor P19 is frequently used to boost protein overexpression by preventing the decline of the transgene transcript levels (Van der Hoorn *et al*., 2003).

The versatility and potential of agroinfiltration are illustrated by many use cases. For instance, production of biopharmaceuticals (molecular farming) (Stoger *et al*., 2014) in agroinfiltrated *N. benthamiana* offers speed, scalability and low risk of contamination with human pathogens when compared to classical insect or mammalian cell culture systems. An agroinfiltration‐based expression platform can now deliver ten million doses of the latest influenza vaccine within a record time of 6 weeks (Pillet *et al*., 2016). Large‐scale agroinfiltration has also produced many different functional monoclonal antibodies (Yusibov *et al*., 2016), including the Ebola neutralizing drug ZMapp (Qiu *et al*., 2014). Transient, spatially restricted overexpression of synthetic biology building blocks can shift plant secondary metabolism towards valuable products with minor impact on fitness (Nielsen *et al*., 2013). Along similar lines, pathogen‐derived effectors that would likely have severe phenotypic effects if expressed in stable lines have been studied by agroinfiltration (Bos *et al*., 2006; Dagdas *et al*., 2016; Petre *et al*., 2016). Speed and simplicity of agroinfiltration are leveraged for high‐throughput screening of fluorescently tagged proteins to study their subcellular localization (Martin *et al*., 2009).

Although agroinfiltration is a widely used tool, remarkably little is known about how *N. benthamiana* responds to agroinfiltration. Agrobacterium elicits immune responses, including the induction of pathogenesis‐related (PR) genes and the accumulation of extracellular PR proteins (Goulet *et al*., 2010; Pitzschke, 2013; Zhou *et al*., 2017). As in other plants, this immune response reduces subsequent pathogen infections (Li *et al*., 2017; Rico *et al*., 2010; Robinette and Matthysse, 1990; Sheikh *et al*., 2014) and may limit transgene delivery. Transgene delivery in older, flowering *N. benthamiana* is limited due to the perception of Agrobacterium cold‐shock protein (Saur *et al*., 2016). In younger plants, which are used for agroinfiltration, responses are elusive. Furthermore, the impact of silencing inhibitor P19 on the response to agroinfiltration and its timing are unresolved.

We focus on extracellular proteases, as they may limit the accumulation of recombinant proteins (RPs) passing through the secretory pathway to become glycosylated. Proteolytic degradation is a bottleneck on the way to industrial viability of agroinfiltration (Mandal *et al*., 2016). Indeed, RP degradation can occur in the extracellular space (Hehle *et al*., 2011) and proteolysis hampers yield and purity of biopharmaceuticals produced in *N. benthamiana* (Hehle *et al*., 2015; Mandal *et al*., 2014; Niemer *et al*., 2014). Papain‐like Cys proteases can degrade RPs *in vitro* (Paireder *et al*., 2016, 2017), but the proteases degrading RP *in planta* are unidentified. Extracellular proteases commonly accumulate in leaves during immune responses. The extracellular tomato Ser protease P69 and Cys proteases Pip1 and Rcr3, for example, accumulate upon infection with viroids, oomycetes, fungi and bacteria (Jordá *et al*., 1999; Kaschani *et al*., 2010; Tian *et al*., 2004). Transcripts and proteins corresponding to proteases also accumulate in Arabidopsis infected with *Pseudomonas* (Xia *et al*., 2004; Zhao *et al*., 2003), and extracellular Ser and Cys protease activities increase in tomato upon fungal infection with *Cladosporium fulvum* (van Esse *et al*., 2008; Sueldo *et al*., 2014). These examples indicate that activity and/or abundance of extracellular proteases, especially Ser and Cys proteases, may increase in *N. benthamiana* upon agroinfiltration, linking proteolytic RP degradation to plant immunity. Therefore, both comprehensive annotation of the *N. benthamiana* protease repertoire and improved understanding of the response to agroinfiltration are needed to limit undesired proteolysis. RP accumulation has been increased by depleting proteases by knockdown in rice cell cultures (Kim *et al*., 2008) and in *Nicotiana tabacum* (Duwadi *et al*., 2015; Mandal *et al*., 2014) and protease inhibitor overexpression in *N. benthamiana* (Goulet *et al*., 2012; Sainsbury *et al*., 2013). These studies indicate that once targets are identified, protease depletion could improve agroinfiltrated *N. benthamiana* as a protein expression platform.

Here, we investigated how RP production may be affected by the immune response to agroinfiltration, especially immune proteases. Time‐resolved leaf transcriptome and extracellular proteome data sets of agroinfiltrated leaves revealed an immune response that is mounted at the expense of photosynthesis and not affected by P19. We analysed the exceptionally large *N. benthamiana* protease repertoire in the context of other plant proteases and identified active Ser and Cys proteases. Taken together, the data will advance strategies to improve transient protein expression by engineering plant immunity and depleting proteases.

## Results and discussion

**Figure 1:**
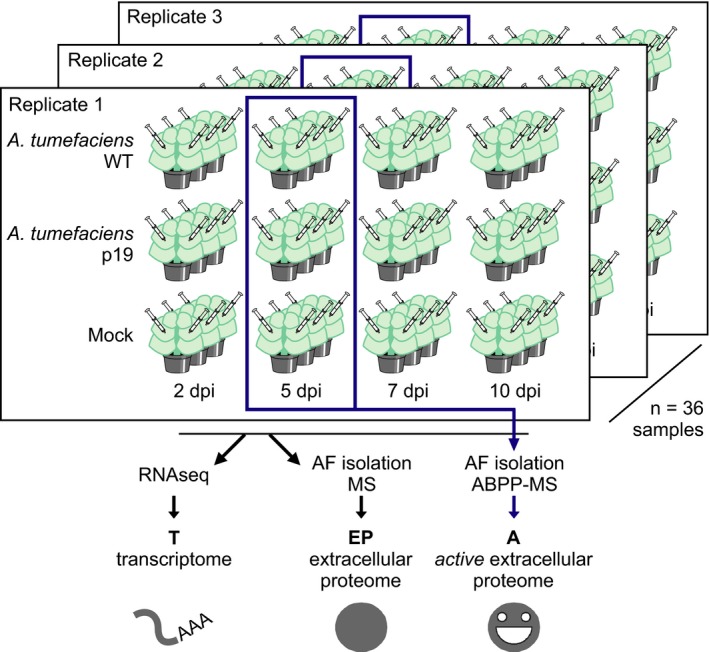
Experimental setup. Leaves of *Nicotiana benthamiana* were infiltrated with *Agrobacterium* GV3101‐pMP90 without any T‐DNA plasmid (WT) or carrying a plasmid for P19 expression (P19) or with buffer (mock). Abbreviations: dpi, days postinfiltration; RNAseq, mRNA sequencing; AF, apoplastic fluid; MS, protein mass spectrometry; ABPP, activity‐based protein profiling.

To characterize agroinfiltrated *N. benthamiana* leaves, we infiltrated *N. benthamiana* leaves with wild‐type *A. tumefaciens* GV3101‐pMP90 (no binary vector, WT), Agrobacterium P19 (T‐DNA encoding viral silencing suppressor P19 (Chapman *et al*., 2004), P19) or buffer (mock treatment). We took samples at two, five, seven and 10 days postinfiltration (dpi) and quantified transcripts, extracellular proteins and extracellular protein activity using RNAseq, label‐free quantification mass spectrometry (MS) and activity‐based proteomics (activity‐based protein profiling coupled to mass spectrometry, ABPP‐MS) (Figure 1).

During a first annotation of the transcriptome and proteome data, we observed that well‐known proteases including papain‐like Cys proteases (PLCPs, MEROPS family C01) and subtilases (family S08) often appeared truncated or lacked conserved domains in the Niben101 proteome database (https://solgenomics.net/). To obtain a database with protease families that are adequately annotated for the evaluation of transcriptomics and proteomics experiments, we compared four *N. benthamiana* proteome databases and manually curated the proteases in the best database (described in detail in Appendix S1). Searching the extracellular proteome MS spectra with our curated proteome, we identified peptides corresponding to 30 proteins more than with the best published database, showing that the curation improved interpretation of experimental data (Appendix S1).

### The *N. benthamiana* response to agroinfiltration

#### The P19 silencing suppressor has minor effects on the transcriptome and no effect on the extracellular proteome of *N*. *benthamiana*

**Figure 2:**
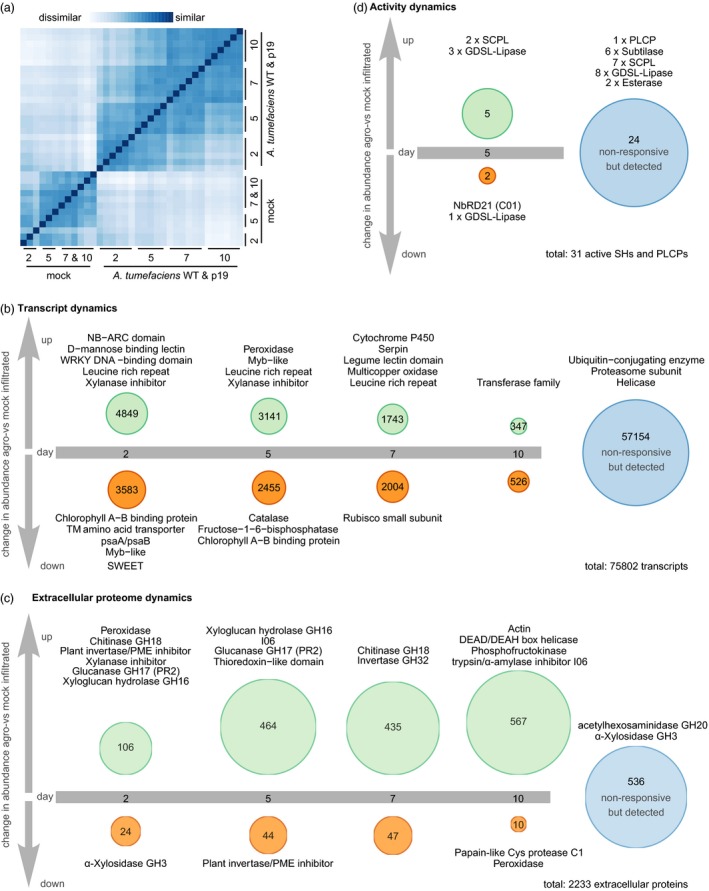
The immune response to agroinfiltration entails an increase in the extracellular proteome and is not affected by P19 overexpression. (a) Euclidean sample distances between the transcriptomes obtained from all 36 samples. Samples were ordered by hierarchical clustering based on the sample distances. (b, c): Transcripts (b) or proteins (c) were grouped by when their abundance first changed significantly (Wald test for transcripts, Student's *t*‐test for proteins; Benjamini–Hochberg (BH) adjusted *P* < 0.05) and more than twofold in (WT and P19) agroinfiltrated samples compared to mock‐infiltrated samples. Annotations given above the circles are representatives of the PFAM families that are significantly (Hypergeometric test, BH‐adjusted *P* < 0.05) overrepresented in the respective regulatory category compared to all detected transcripts (b) or proteins (c). Protein groups for which corresponding peptides were identified are counted as one protein. (d) Activity of extracellular PLCPs and Ser hydrolases was assayed by ABPP‐MS at 5 dpi, counting each protein group for which peptides were identified as one active protein. Proteins were grouped by whether they were enriched in the WT agroinfiltrated samples, controls or both (*t*‐test probe sample vs no‐probe control, BH‐adjusted *P* < 0.1). Differences in abundance between agroinfiltrated samples and controls were not significant in any case. Only proteins annotated as SHs or PLCPs are included in the figure. Full data sets are given in Tables S4 and S5 (a&b), S6 and S7 (c) and S11 (d). The R code to generate the figures is given in Appendices S2 (a, b), S3 (c) and S5 (d).

To assess how *N. benthamiana* responds to agroinfiltration and how silencing suppression affects these responses, we sequenced mRNA from WT agroinfiltrated, P19 agroinfiltrated and mock‐infiltrated leaves. Euclidean distance clustering revealed that transcriptomes from agroinfiltrated samples cluster together by time point regardless of whether WT or P19 bacteria were present (Figure 2a). Surprisingly, only 0.75% of all detected transcripts (569/75802) differed significantly in abundance at any time point between WT and P19 agroinfiltrated leaves (Table S1). Among the differentials is the transcript encoding P19, which was very abundant up to 7 dpi and slightly decreased in abundance at 10 dpi, potentially because older leaves are less transcriptionally active (Figure S1). Transcripts encoding components of the silencing machinery such as members of the Argonaute PFAM family were significantly enriched among the transcripts with differential abundance between P19 and WT agroinfiltrated leaves, but most of the differential transcripts (404 of 569) are not annotated (Figure S1 and Table S2). There were no significant differences between extracellular proteomes from WT and P19 agroinfiltrated leaves at any time point (Table S3). We thus compare agroinfiltrated (WT and P19) to mock‐infiltrated leaves for further analysis.

#### Agroinfiltration induces leaf transcriptome changes associated with immune responses

Of all detected transcripts (*n* = 75 802), 24.6% (*n* = 18 648) significantly changed more than twofold in abundance at any time point and were thus considered differential in abundance. Among the differentials, the biggest category (*n* = 4849) is that of transcripts increasing in abundance for the first time at 2 dpi (6.4% of the transcriptome) (Figure 2b, Table S4, Appendix S2). In this category, transcripts encoding proteins associated with immunity are overrepresented (representatives in Figure 2b, complete lists in Table S5). This includes transcripts encoding LRR (leucine‐rich repeat) domain containing receptors such as the recently identified receptor for Agrobacterium cold‐shock protein NbCSPR (Niben101Scf03240g00007) (Saur *et al*., 2016), as well as signalling components carrying NB‐ARC (nucleotide‐binding adaptor shared by Apaf‐1, resistance proteins and CED‐4) and WRKY domains. Among the categories of transcripts whose abundance first increases at 5 or 7 dpi, transcripts encoding Myb transcription factors and serpins, LRRs and xylanase inhibitors are overrepresented. Besides transcripts encoding proteins associated with immune signalling and first‐line defence, we detected a 4.5‐fold average decrease in abundance of 13 transcripts encoding SWEET sugar efflux transporters, which may decrease the nutrient content of the extracellular space to control bacterial growth (Chen, 2014). Differential abundance of transcripts encoding both generators and quenchers of reactive oxygen species, as well as increased accumulation of transcripts encoding cytochrome P450 enzymes, shows that the plants are stressed upon agroinfiltration. Transcripts encoding members of the photosynthetic machinery and assimilatory metabolism in general are enriched among the transcripts decreasing in abundance from 2 dpi onwards, explaining the chlorotic phenotype of agroinfiltrated leaves (Pruss *et al*., 2008). Among the transcripts detected constantly, transcripts encoding for housekeeping proteins like members of the ubiquitin‐proteasome system and helicases are overrepresented. In summary, agroinfiltration is associated with an immune response mounted at the expense of photosynthesis.

#### Diversity and abundance of extracellular proteins increase upon agroinfiltration

We evaluated the effect of agroinfiltration on the extracellular proteome of *N. benthamiana* because the leaf extracellular space is the target site for glycoprotein accumulation in molecular farming, as well as the primary site of interaction with Agrobacterium and thus a promising site for improvement of the transient expression platform. Of all *N. benthamiana* proteins for which we identified extracellular peptides (*n* = 2233 protein groups as defined by MaxQuant (Tyanova *et al*., 2016)), the vast majority (*n* = 1697, 75.9%) changed significantly and more than twofold in abundance and were thus considered differential in abundance. Among these differentials, most (*n* = 1572, 92.6%) increased in abundance upon agroinfiltration (Figure 2c, Table S6, Appendix S3). The increase in the extracellular proteome was mirrored by a corresponding increase in protein concentration in apoplastic fluid (AF) from agro‐ but not mock‐infiltrated samples (Figure S2). Abundant intracellular housekeeping proteins such as actin, helicases and phosphofructokinases are overrepresented in the category of proteins that first increased in abundance at 10 dpi (*n* = 567), indicating that the interaction between *N. benthamiana* and Agrobacterium leads to cell content leakage at this late stage. Leakage may occur *in vivo* and during apoplastic fluid extraction.

Among proteins that first increased in abundance at 2 and 5 dpi, hydrolytic enzymes and inhibitors are overrepresented (Figure 2c and Table S7). This includes classical defence proteins such as xylanase inhibitors, chitinases (GH18) and pathogenesis‐related protein 2 (PR2, a GH17 glucanase) (Cosgrove, 2016). PR2 accumulation upon agroinfiltration is consistent with an earlier study (Goulet *et al*., 2010). Cell wall remodelling xyloglucan endotransglycolases/hydrolases (GH16) and versatile I06 α‐amylase/Ser protease inhibitors may contribute indirectly to plant defence. Family C01 proteases (PLCPs) are overrepresented in the small category of proteins that first decreased in abundance at 10 dpi. Thus, *N. benthamiana* extracellular PLCPs do not increase as strongly and persistently in abundance upon agroinfiltration as tomato extracellular PLCPs do upon pathogen challenge (van Esse *et al*., 2008). In contrast, PLCPs may localize to intracellular compartments as shown for RD21 in Arabidopsis (Hayashi *et al*., 2001) or may be degraded in the extracellular space. Invertase/pectin methyl esterase (PME) inhibitors are overrepresented both in the category of proteins first increasing at two and in the category of proteins first decreasing at 5 dpi in abundance. Invertase inhibition upon agroinfiltration thus appears to be transient, and indeed, invertases (GH32) are overrepresented in the category of proteins first increasing in abundance at 7 dpi. Plant invertases cleave the transport sugar sucrose, providing vital nutrients to sink tissues (Goetz *et al*., 2001). The chlorotic agroinfiltrated leaves may be less photosynthetically active and lose nutrients to the bacteria, turning them from a source into a sink organ. Some members of the GH32 family degrade extracellular polysaccharides from pathogens (Limoli *et al*., 2015), suggesting that GH32 family members may promote both nutrition and defence in agroinfiltrated leaves. GH3 α‐xylosidases are overrepresented in both the category of proteins first decreasing in abundance at 2 dpi and in the category of proteins with constant abundance. Some GH3 family members act in cell wall remodelling and others locally adjust auxin concentrations as auxin‐amido synthetases (Shigeyama *et al*., 2016; Zheng *et al*., 2016). This dual role may explain why GH3 members are overrepresented in both regulatory categories. Along with hydrolases and inhibitors, peroxidases and thioredoxins are overrepresented in several regulatory categories. These modulators of ROS levels facilitate both immune signalling and cell wall remodelling by extracellular ROS (Ivanchenko *et al*., 2013).

Besides plant proteins, we also identified peptides from bacterial proteins in the extracellular proteome of agroinfiltrated leaves. In fact, Agrobacterium proteins make up a quarter of the extracellular proteins in agroinfiltrated samples (738 bacterial vs 2233 plant proteins) and appear to mostly function in providing nutrients to the bacteria. Highly abundant bacterial proteins are ABC transporters, cytochrome P450 proteins and porins. This may include cytoplasmic bacterial proteins released into the extracellular proteome upon cell death or during the extraction of apoplastic fluid. We identified peptides corresponding to 17 different Agrobacterium proteases, including six Ser proteases, in the extracellular space (Table S8), but did not identify peptides from bacterial proteases using ABPP‐MS.

Ageing of leaves irrespective of their treatment during our 10‐day time course is associated with induction of defence and decrease in primary metabolism. 18.9% of detected transcripts and 6.5% of identified extracellular proteins changed significantly in abundance over time independent of the treatments. Analysis of predicted functions overrepresented among the changing transcripts and proteins suggests that while PLCPs, P450‐domain‐containing proteins and PR proteins accumulate, components of the photosynthetic machinery, histones and cytoskeleton elements decrease in abundance (R code in Appendix S4, data in Tables S9 and S10).

#### The repertoire of active extracellular PLCPs and Ser hydrolases is modulated, but not drastically expanded upon agroinfiltration

We next investigated extracellular hydrolase activity to identify active candidate proteases for depletion, focusing on Ser and Cys proteases because plant immune responses often result in increased abundance and activity of these protease classes and Cys proteases can degrade biopharmaceuticals *in vitro* (Paireder *et al*., 2016, 2017). We performed activity‐based protein profiling (ABPP) with probes targeting papain‐like Cys proteases (PLCPs) and serine hydrolases (SHs) (Greenbaum *et al*., 2002; Kaschani *et al*., 2009). Both probes consist of a specific inhibitor that covalently binds the active site of their respective targets, a linker and a biotin tag used for enrichment of active enzymes from extracellular proteomes prior to MS analysis. Both probes have been validated in plants using target detection, genetic target depletion and inhibition of probe binding with independent protease inhibitors, confirming that reactivity to the probe indicates the availability of the active site and thus enzyme activity (Kovács and van der Hoorn, 2016). We focused on 5 dpi when the response to agroinfiltration is fully developed. We identified peptides corresponding to two PLCPs and 29 SHs, 17 of which are proteases that were enriched from the extracellular proteome using ABPP‐MS. (Figure 2d). The abundance of peptides corresponding to one Clade II and one Clade III SCPL was increased upon agroinfiltration, indicating increased activity and/or abundance. In contrast, peptides corresponding to an RD21‐like PLCP were only identified in ABPP‐MS samples from mock‐infiltrated plants, indicating depletion of enzyme activity upon agroinfiltration. The abundance of extracellular peptides from this PLCP remained constant upon agroinfiltration at 5 dpi, suggesting a post‐translational regulatory mechanism. We identified peptides corresponding to six subtilases (one SBT5 and five SBT1 subtilases, including the proteins clustering with tomato P69), seven SCPLs (two Clade IB, three Clade II, four cade III) and one aleurain‐like PLCP with similar abundance in both agro‐ and mock‐infiltrated samples, indicating that activity of these enzymes remains constant upon agroinfiltration. This is surprising, as in tomato, active extracellular subtilases and PLCPs drastically increase in abundance and diversity during immune responses (van Esse *et al*., 2008; Sueldo *et al*., 2014). Besides the proteases, we identified peptides corresponding to 14 additional SHs annotated as lipases and esterases. Peptides from three GDSL lipases (containing a GDSL sequence motif) increased in abundance upon agroinfiltration, while peptides from one GDSL‐lipase decreased. Adjustment of extracellular GDSL‐lipase activity may contribute to immune signalling, as lipases regulate salicylic acid as well as ethylene signalling in Arabidopsis (Falk *et al*., 1999; Kim *et al*., 2013b) and upon powdery mildew infection, lipase‐encoding transcripts accumulate in grapevine (Szalontai *et al*., 2012) (R code in Appendix S5, data in Table S11).

#### The extracellular proteome and active secretome is under post‐transcriptional and post‐translational control

**Figure 3:**
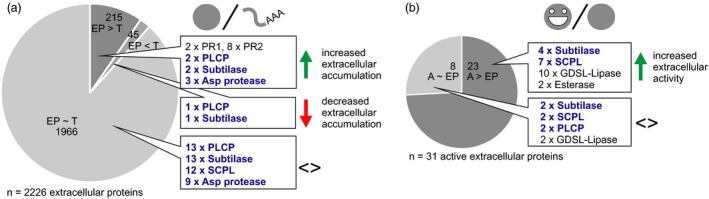
Post‐transcriptional and post‐translational control over the extracellular proteome. Fold changes in response to agroinfiltration at 5 dpi were compared between abundances of extracellular proteins and their transcripts (a) and between activity and abundance of extracellular proteins (b) (Student's *t*‐test; BH‐adjusted *P* < 0.1). Protein groups of interest are named next to the pie charts. Full data sets are given in Tables S12 and S13. The R code of the analysis is given in Appendix S6. T, fold change of transcript abundance; EP, fold change of extracellular protein abundance; AEP, fold change of activity of the extracellular protein.

Having transcriptome, extracellular proteome and active secretome data creates a unique opportunity to detect discrepancies in abundance changes between transcripts, total extracellular proteins and active extracellular proteins. To assess how much post‐transcriptional regulation shapes the extracellular proteome, we compared the fold changes of extracellular protein abundance and transcript abundance at 5 dpi, when the response to agroinfiltration is fully developed (Figure 3a, Appendix S6, Table S12). The extracellular protein (EP) was increased more or decreased less in abundance than its corresponding transcript (T) for 215 (9.7%) of the 2226 extracellular proteins for which we detected the corresponding transcript (significant difference between the fold changes, EP > T). Among these proteins are two PR1 proteins, eight PR2 glucanases and two P69‐like subtilases (PR7). This finding indicates that the immune response is accompanied by efficient extracellular protein delivery, as previously suggested based on transcriptional up‐regulation of the secretory pathway during immunity (Wang *et al*., 2005). More efficient extracellular delivery may be accompanied by enhanced stability of the secreted proteins. In addition to classical PR proteins, two PLCPs (one XCP and one RD19‐like) and three pepsin‐like aspartic proteases appeared efficiently delivered to the extracellular space with EP > T, suggesting they may be candidate immune proteases. Only 45 extracellular proteins (2.0%) increased less or decreased more in abundance than expected from their transcript level changes (EP < T).

Interestingly, for 23 of the 31 active enzymes for which we also detected the extracellular protein (74.2%), abundance of the active protein (A) increased more or declined less than expected based on changes in total extracellular protein abundance (A > EP) (Figure 3b, Supporting Table S13). This suggests that hydrolase activity is frequently post‐translationally controlled. Among the proteins with A > EP are four subtilases, three of which contain I09 domains and are thus likely activated by cleavage upon agroinfiltration. Activation by cleavage may explain why abundance of all active subtilases remained constant, while total protein abundance decreased in five cases. Seven SCPLs also remained constant or increased in active protein abundance although their total protein abundance decreased (Table S13). As SCPLs lack inhibitory domains, they may undergo post‐translational activation by release from an inhibitor or autoactivation triggered by pH or redox‐level changes. Taken together, efficient extracellular delivery influences the increase in the extracellular proteome upon agroinfiltration and many hydrolases for which we identified peptides by ABPP‐MS appear to be activated post‐translationally.

#### *N. benthamiana* deploys a large, diverse repertoire of proteases in agroinfiltrated leaves

**Figure 4:**
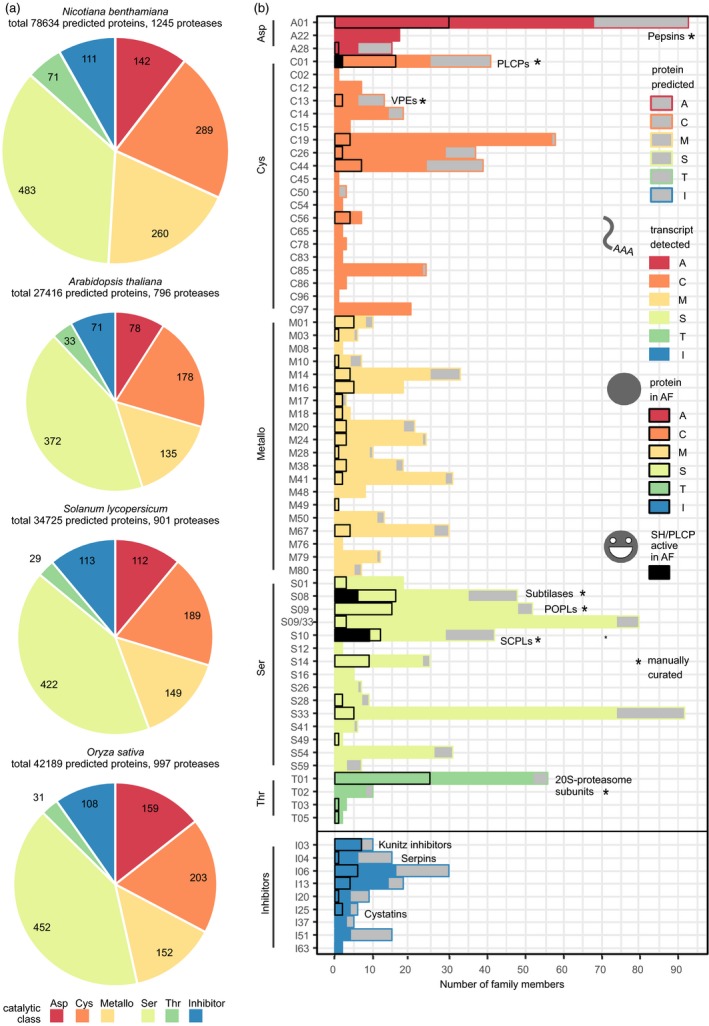
*Nicotiana benthamiana* has a diverse protease and protease inhibitor repertoire. (a) Number of proteases and noncatalytic protease homologs in each catalytic class or inhibitors annotated are given for each species (*N. benthamiana* curated proteome, *Arabidopsis thaliana* TAIR10, *O. sativa* v7 JGI,* S. lycopersicum* ITAG2.4). The area of each pie chart is scaled by the total number of proteases and inhibitors. (b) The protease and protease inhibitor repertoire of *N. benthamiana*. For each MEROPS family, bars give the size in the predicted proteome (grey), the number of transcripts detected in mock and/or agroinfiltrated leaves (filled), the number of proteins for which we detect corresponding extracellular peptides in agro‐ and/or mock‐infiltrated leaves (black outline) and the number of enzymes for which peptides were detected in ABPP‐MS, indicating activity (black fill). For the manually curated families (marked by an asterisk), protease homologs lacking the active site were not counted. Each protein group identified in MS and ABPP‐MS was counted as one family member. Note that due to the nature of the ABPP probes used, only SHs and PLCPs were monitored on the activity level. The S09 (prolyl oligopeptidase) and S33 (prolyl aminopeptidase) families share the α/β‐hydrolase fold (PFAM families PF12695 and PF12697), and sequences with only these PFAM identifiers are marked S09/S33.

To improve protease annotation, we analysed the *N. benthamiana* protease repertoire in the context of known plant proteases, using the MEROPS nomenclature. The MEROPS database of proteases and inhibitors defines families based on protein sequence homology that are grouped into clans based on structural homology. Protease family names consist of a letter denoting the catalytic class and a unique number (i.e. A01 for pepsin‐like aspartic proteases) (Rawlings *et al*., 2014). We identified 1245 proteases and noncatalytic protease homologs in the curated proteome of *N. benthamiana*. A smaller protease repertoire is encoded by genomes of three crop and model plants: Arabidopsis (796 proteases), tomato (901) and rice (997) (Figure 4a and Table S14). Although the *N. benthamiana* protease repertoire is much larger, the proportion of predicted proteins annotated as proteases is higher in the other plants (2.9% in Arabidopsis, 2.6% in tomato and 2.4% in rice) than in *N. benthamiana* (1.6%). The lower proportion of proteases in *N. benthamiana* may reflect the suboptimal genome annotation.

To characterize functional proteases and protease inhibitors in agroinfiltrated leaves, we analysed them at three levels. First, we detected transcripts for 975 proteases and 60 inhibitors. Second, we identified extracellular peptides from 196 proteases and 21 inhibitors, including proteases from every catalytic class. Third, we identified 17 active extracellular Ser and Cys proteases in agroinfiltrated leaves (Figure 4b). The most prominent features of the *N. benthamiana* protease repertoire are the large numbers of Cys, Metallo‐ and Thr proteases. Among the Cys proteases, the metacaspase family C14 is doubled in size (*n* = 18 members) compared to Arabidopsis (*n* = 9), tomato (*n* = 9) and rice (*n* = 8). We did not identify extracellular peptides corresponding to metacaspases, although 14 had detectable transcripts. The large number of metalloproteases in *N. benthamiana* (*n* = 260) compared to Arabidopsis (*n* = 135), tomato (*n* = 149) and rice (*n* = 152) is distributed among 20 families, and we identified extracellular peptides corresponding to members of most metalloprotease families. 10% (*n* = 26) of the metalloprotease‐encoding genes increased in transcript abundance upon agroinfiltration, while 13% (*n* = 35) decreased. In contrast, 75% (*n* = 28) of the metalloproteases for which we identified extracellular peptides increased in abundance and only one M28 protease decreased. Very few plant metalloproteases are functionally characterized, including AtSOL1, an M14 carboxypeptidase processing peptide hormones (Casamitjana‐Martínez *et al*., 2003; Tamaki *et al*., 2013) and AtPreP1 and 2, Arabidopsis M16 proteases cleaving organellar target peptides (Bhushan *et al*., 2005). The *N. benthamiana* M10 protease NMMP1 has been implicated in defence because silencing *NMMP1* confers susceptibility to bacterial pathogens (Kang *et al*., 2010). The transcript corresponding to NMMP1 (Niben101Scf10336XLOC_078719) increases in abundance upon agroinfiltration, while its extracellular peptides appear constant. The large metalloprotease repertoire of *N. benthamiana* is changing upon agroinfiltration, raising the question whether these metalloproteases might regulate the immune response through protein processing. The high number of Thr proteases (*n* = 71) compared to Arabidopsis (*n* = 34), tomato (*n* = 29) and rice (*n* = 31) is due to drastic expansion of the T01 family in *N. benthamiana* (*n* = 65, vs *n* = 24 in Arabidopsis, *n* = 20 in tomato and *n* = 23 in rice). T01 contains the α and β subunits of the 20S core protease of the proteasome. Phylogenetic analysis showed that *N. benthamiana* has more representatives of each subunit (Figure S3). We identified extracellular peptides corresponding to 25 T01 subunits, possibly due to cell content leakage. As transcripts of most (*n* = 52) T01 subunits were detected in leaves, multiple versions of the 20S proteasome may co‐exist, as they do in Arabidopsis (Book *et al*., 2010). Indeed, we recently showed that two sets of catalytic subunits are incorporated in functional 20S proteasomes in *N. benthamiana* (Misas‐Villamil *et al*., 2017).

In contrast to the protease repertoire, the protease inhibitor repertoire of *N. benthamiana* (*n* = 111 predicted protease inhibitors) is not much larger compared to tomato (*n* = 113), rice (*n* = 108) and Arabidopsis (*n* = 71). This apparent discrepancy may reflect the multifunctionality of many protease inhibitors (Grosse‐Holz and van der Hoorn, 2016) and incomplete annotation. Among the annotated protease inhibitors, the I03 (Kunitz) inhibitors probably function extracellularly, as we identified extracellular peptides corresponding to all seven Kunitz (family I03) inhibitors for which we detected transcripts. Kunitz inhibitors can inhibit both subtilases (family S08) and α‐amylases, but their bifunctional structure can also target other Ser or Cys proteases, and other proteins (Renko *et al*., 2012). *N. benthamiana* serpins (I04) appear to mostly be intracellular, as we detected six serpin‐encoding transcripts, but identified corresponding extracellular peptides for only one. Serpins have been found in both the cytoplasm (Lampl *et al*., 2013) and the extracellular space (Ghorbani *et al*., 2016), and regulate plant defence and programmed cell death through irreversible inhibition of Ser and Cys proteases (Bhattacharjee *et al*., 2017; Lampl *et al*., 2013). We detected transcripts for four and identified extracellular peptides corresponding to two cystatins (family I25). Cystatins target PLCPs and VPEs, regulating storage protein accumulation, germination and defence (Benchabane *et al*., 2008; Grosse‐Holz and van der Hoorn, 2016).

**Figure 5:**
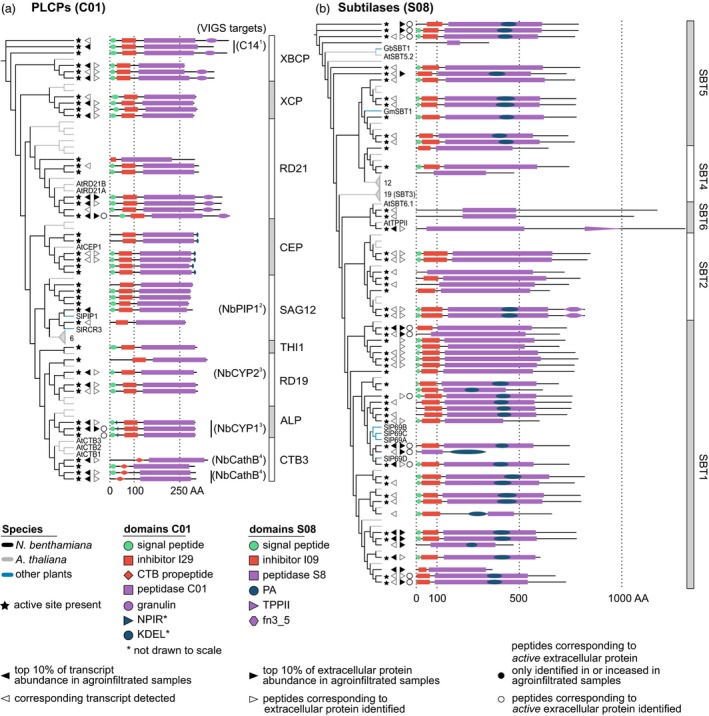
Annotation and detection of extracellular papain‐like Cys proteases (PLCPs) and subtilases in *Nicotiana benthamiana*. Phylogenetic trees based on the protein sequences of PLCPs (a) and subtilases (b) containing all proteases and protease homologs in the respective family in Arabidopsis (grey branches) and *N. benthamiana* (black branches), supplemented by well‐studied enzymes from other plant species (blue branches). Names are given as two‐letter species abbreviation followed by the name used in the literature. Grey triangles denote collapsed subtrees that contain only Arabidopsis sequences, with the number of proteins given next to the triangle. For protein abundance and activity, the respective symbols are shown next to all members of each protein group for which corresponding peptides were identified. VIGS targets were predicted based on >90% identical residues between the fragment used for VIGS and the respective transcript. References: 1 (Kaschani *et al*., 2010); 2 (Xu *et al*., 2012); 3 (Hao *et al*., 2006); 4 (Gilroy *et al*., 2007). Abbreviations: CTB, cathepsin‐B‐like; TPP, tripeptidyl‐peptidase; fn3_5, fibronectin‐3 like domain found on streptococcal C5a peptidase.

**Figure 6:**
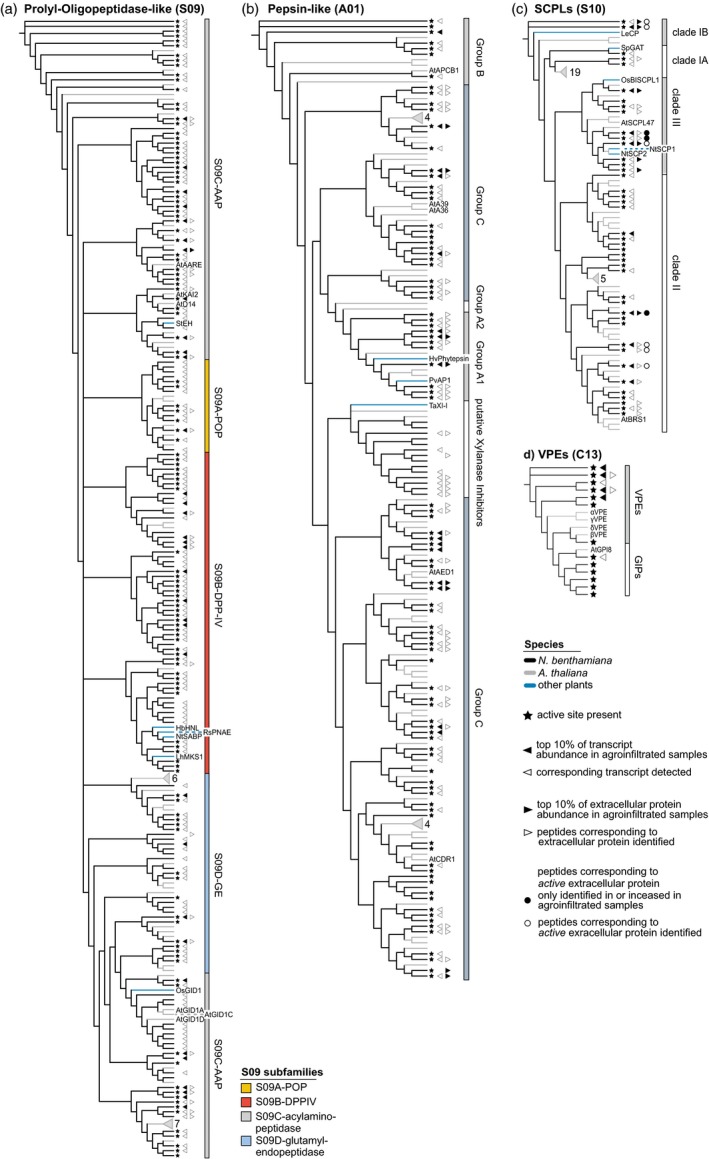
Annotation and detection of additional protease families in *Nicotiana benthamiana*. Phylogenetic trees based on the protein sequences of POPLs (a), pepsin‐like proteases (b), SCPLs (c) and VPEs (d) containing all proteases and protease homologs of the respective family in Arabidopsis (grey branches) and *N. benthamiana* (black branches), supplemented by well‐studied enzymes from other plant species (blue branches). Arabidopsis sequences that only carry the α/β‐hydrolase fold PFAM identifiers are not shown in the S09 tree for readability. Names and symbols are used as described for Figure 5.

Having obtained an overview of the *N. benthamiana* protease and protease inhibitor repertoire, we focused on six large protease families, which we curated manually (Appendix S1). For these six families, we performed phylogenetic analyses to resolve subfamilies and determine which *N. benthamiana* proteins are most similar to previously studied proteases (Figures 5 and 6).

#### The PLCP family is conserved in *N*. *benthamiana*, but PIP1‐ and RCR3‐like PLCPs are absent from the extracellular proteome of agroinfiltrated leaves

*Nicotiana benthamiana* has more papain‐like Cys proteases (PLCPs, family C01, *n* = 41 members) than Arabidopsis (*n* = 36) and tomato (*n* = 36), but less than rice (*n* = 54). PLCP subfamilies can be defined by shared sequence features (Richau *et al*., 2012) (Figure 5a). For example, the NPIR vacuolar localization signal is found in aleurain‐like proteases (ALPs) and the KDEL ER‐retention signal in Cys endopeptidases (CEPs). Cathepsin‐B‐like proteases (CTBs) have a specific prodomain (PF08127) serving as chaperone and inhibitor, like the I29 (PF08246) prodomain for other PLCPs. Most *N. benthamiana* PLCPs have a secretion signal predicted by SignalP (Dyrløv Bendtsen *et al*., 2004). Accordingly, we identified extracellular peptides corresponding to 18 of the 25 PLCPs for which we detected transcripts. Among the extracellular PLCPs are three granulin‐carrying proteases similar to the immune protease AtRD21 (Shindo *et al*., 2012), three Cathepsin B‐like proteases (CTBs) and NbCYP1 and NbCYP2, which limit susceptibility to fungal pathogens (Hao *et al*., 2006). We identified peptides in ABPP‐MS from NbRD21 and NbCYP1, indicating that these proteases are active extracellularly. Many of the PLCPs for which we detected transcripts and identified extracellular peptides contribute to *N. benthamiana* immunity. For instance, silencing *NbCathB* (Gilroy *et al*., 2007; McLellan *et al*., 2009) blocks the hypersensitive response (HR) and *NbC14/CP14* silencing confers susceptibility to *Phytophthora infestans* (Bozkurt *et al*., 2011; Kaschani *et al*., 2010). Surprisingly, we did not identify extracellular peptides corresponding to the *N. benthamiana* proteins clustering with the tomato immune proteases PIP1 (Tian *et al*., 2004) and RCR3 (Krüger *et al*., 2002), although we detected NbPIP1‐ and NbRCR3‐encoding transcripts.

#### P69‐like SBT1 subtilases are abundant and active in the extracellular proteome of agroinfiltrated *N. benthamiana* leaves

The *N. benthamiana* subtilase family (S08, *n* = 56 members) is the same size as in Arabidopsis (*n* = 56) and smaller than in tomato (*n* = 90) and rice (*n* = 61) (Figure 5b). We identified extracellular peptides for 28 of the 39 subtilases whose transcript we detected; 12 of the 28 subtilases for which we identified extracellular peptides were among the top 10% most abundant extracellular proteins and we identified peptides corresponding to 11 active subtilases using ABPP‐MS (Figure 5b). Across the whole subtilase family, the I09 prodomain is well conserved and the PA dimerization domain (Rose *et al*., 2010) is present in some members of each subfamily. An exception lacking SP, I09 and PA domains are the basal SBT6 subtilases (Taylor and Qiu, 2017). We detected transcripts encoding three *N. benthamiana* SBT6 subtilases and identified extracellular peptides from one. SBT6 subtilases can process peptide hormones regulating cell elongation (Ghorbani *et al*., 2016), or degrade peptides released by the 26S proteasome (Book *et al*., 2005). Remarkably, the SBT1 subfamily is threefold larger in *N. benthamiana* (*n* = 30 members) than in Arabidopsis (*n* = 9), while the SBT3 subfamily is absent in *N. benthamiana*. We detected 22 SBT1 subtilase‐encoding transcripts and identified corresponding extracellular peptides for 19. We also identified peptides corresponding to eight active SBT1 subtilases by ABPP‐MS. The tomato P69A, B and C subtilases (Jordá *et al*., 1999) cluster with the SBT1 subfamily, which is consistent with a recently published, updated phylogeny of the subtilase family (Taylor and Qiu, 2017). We detected transcripts for eight and identified extracellular peptides corresponding to four SBT5 subtilases. We also identified peptides corresponding to three active SBT5 subtilases by ABPP‐MS. SBT5 subtilases can regulate plant immunity as receptors (Duan *et al*., 2016), as transcription factor binding proteins (Serrano *et al*., 2016) or being processed to release peptide hormones (Pearce *et al*., 2010). The updated subtilase phylogeny (Taylor and Qiu, 2017) suggests to split SBT6 into two subfamilies and notes that the distinction between SBT4 and SBT5 subfamilies is weakly supported. Indeed, we find two subclades of SBT6 in *N. benthamiana*, clustering with one of the Arabidopsis representatives each. SBT4 falls into a Clade containing part of SBT5 in our tree, indicating the updated phylogeny agrees with our curated *N. benthamiana* proteome.

#### *Nicotiana benthamiana* POPLs are underrepresented in the extracellular proteome of agroinfiltrated leaves

The prolyl oligopeptidase‐like (S09, POPL) family in *N. benthamiana* (*n* = 180 members) is equivalent in size with the POPL families in Arabidopsis (*n* = 143), tomato (*n* = 162) and rice (*n* = 196) (Figure 6a). The S09 family is defined via the α/β‐hydrolase fold (PF12695 and PF12697), but these proteins are not always proteases (Mindrebo *et al*., 2016). Interestingly, we only identified extracellular peptides for 18 of the 122 family members for which we detect a transcript (15%), suggesting that *N. benthamiana* POPLs are primarily intracellular. S09A (prolyl oligopeptidase‐like, POPL) is the smallest subfamily and no plant POPL has been functionally characterized, but we detected 14 POPL‐encoding transcripts and identified extracellular peptides from two POPLs. The S09B dipeptidyl‐peptidase type IV (DPP‐IV) subfamily contains membrane‐bound exopeptidases (Tripathi and Sowdhamini, 2006), but also clusters with nonproteolytic α/β‐hydrolases, including HbHNL, RsPNAE and LhMKS1 and the salicylic acid receptor NtSABP2 (Auldridge *et al*., 2012; Dogru *et al*., 2000; Forouhar *et al*., 2005; Wagner *et al*., 1996). Interestingly, we only detected a transcript for NbSABP2. We identified extracellular peptides from four DPP‐IVs. We also detected transcripts for four and identified peptides for two aminoacyl‐removing peptidases (AAPs/AAREs, subfamily S09C) clustering with AtAARE. AtAARE is implicated in the cytoplasmic antioxidative system (Nakai *et al*., 2012; Yamauchi *et al*., 2003). We detected transcripts, but did not identify extracellular peptides for the nonproteolytic members of S09C, including the proteins clustering with the hormone receptors D14 (Yao *et al*., 2016), KAI2 (Guo *et al*., 2013), GID1 (Griffiths *et al*., 2006) and the potato epoxide hydrolase StEH (Stapleton *et al*., 1994).

#### Pepsin‐like aspartic proteases are highly abundant in agroinfiltrated leaves and pepsin‐like xylanase inhibitors have expanded in *N. benthamiana*

Extracellular peptides from pepsin‐like aspartic proteases (A01) were abundantly detected and the A01 family is expanded in *N. benthamiana* (*n* = 110 members) compared to Arabidopsis (*n* = 69) and tomato (*n* = 100), but is smaller than the rice A01 family (*n* = 130) (Figure 6b). We detected 76 A01 protease‐encoding transcripts and identified extracellular peptides from 45 pepsin‐like aspartic proteases. Eight pepsin‐like aspartic proteases were among the top 10% most abundant extracellular proteins. Pepsin‐like aspartic proteases are subdivided into subfamilies A1 (typical pepsin‐like), A2 (typical, but lacking the plant‐specific insert), B (nucellins) and C (atypical) (Faro and Gal, 2005). We detected transcripts and identified peptides for 10 A1 pepsin‐like proteases and they cluster with two enzymes implicated in stress responses, barley phythepsin and bean AP1 (Contour‐Ansel *et al*., 2010; Hückelhoven *et al*., 2001). Group A2 seems absent in the *N. benthamiana* predicted proteome. We detected transcripts, but did not identify extracellular peptides, for five A01 group B proteases. AtAPCB1 in group B is required for autophagy and resistance to *Botrytis* (Li *et al*., 2016). Group C is the largest A01 subfamily in *N. benthamiana*, with 54 A01 Group C‐encoding transcripts detected in leaves and corresponding extracellular peptides identified for 29. Two Arabidopsis members of group C, AtCDR1 and AtAED1, modulate plant defence responses in the extracellular space (Breitenbach *et al*., 2014; Xia *et al*., 2004). AtAED1 clusters with two abundant extracellular *N. benthamiana* proteins. We detected transcripts encoding several and identified extracellular peptides from one protein clustering with AtA36 and AtA39, two putative GPI‐anchored aspartic proteases (Gao *et al*., 2017). Interestingly, the A01 Clade clustering with the wheat xylanase inhibitor TaXI‐I is expanded drastically with 14 members in *N. benthamiana*, compared to two in Arabidopsis (Brutus *et al*., 2005; Sansen *et al*., 2004). TaXIs share the fold of pepsin‐like proteases, but have lost the active site and act as xylanase inhibitors. We detected transcripts for seven putative *N. benthamiana* xylanase inhibitors and identified extracellular peptides corresponding to six.

#### *Nicotiana benthamiana* has an expanded SCPL Clade III in the extracellular proteome of agroinfiltrated leaves

*Nicotiana benthamiana* has less serine carboxypeptidase‐like enzymes (SCPLs, S10, *n* = 42 members) than Arabidopsis (*n* = 54), tomato (*n* = 61) and rice (*n* = 59) (Figure 6c). We detected transcripts for 29 and identified extracellular peptides from 19 SCPLs. We also identified peptides corresponding to nine active extracellular SCPLs by ABPP‐MS. SCPLs fall into four clades (Fraser *et al*., 2005). We detected transcripts for three and identified extracellular peptides corresponding to one member of Clade IA, which contains the *Solanum pennellii* glucose acetyltransferase (SpGAT) (Franziska, 2013). Clade IB contains the wound‐inducible tomato carboxypeptidase LeCP (Moura *et al*., 2001) and two *N. benthamiana* proteins, for which we detected transcripts, identified extracellular peptides and peptides by ABPP‐MS, indicating activity. The largest S10 subfamily is Clade II, with transcripts detected for 16 members and extracellular peptides identified for eight. We also identified extracellular peptides corresponding to four active Clade II SCPLs in ABPP‐MS. Interestingly, Clade III is expanded in *N. benthamiana* (*n* = 10) compared to Arabidopsis (*n* = 5) and well represented in the extracellular proteome, with the encoding transcripts detected and extracellular peptides identified for eight members each. We also identified peptides corresponding to three active Clade III SCPLs in ABPP‐MS. Clade III members such as NtSCP1, NtSCP2 (Bienert *et al*., 2012) and AtSCPL47 (Charmont *et al*., 2005) are extracellular carboxypeptidases, and OsBISCPL1, a rice Clade III SCPL, enhances stress resistance when overexpressed in Arabidopsis (Liu *et al*., 2008).

#### Peptides corresponding to extracellular VPEs are identified in agroinfiltrated *N. benthamiana* leaves

We annotated seven vacuolar processing enzymes (VPEs/legumains/asparaginyl endopeptidases) and six GPI‐anchor transamidases that share the VPE domain architecture (PF01650) in *N. benthamiana*. Together, they constitute family C13 (*n* = 13 members), which is larger than in Arabidopsis (*n* = 5) and rice (*n* = 6), but smaller than in tomato (*n* = 19). We detected five VPE‐encoding transcripts and one NbGIP‐encoding transcript. Notably, we also identified extracellular peptides corresponding to two VPEs, consistent with observations made in tomato (Sueldo *et al*., 2014). VPEs can activate proteins in vacuoles, including proteases (Rojo *et al*., 2003) and protease inhibitors (Heath *et al*., 1995; Mylne *et al*., 2011). Silencing of *NbVPEs* blocks virus‐induced cell death in *N. benthamiana* (Hatsugai *et al*., 2004) and VPEs can also act in other forms of plant cell death (Gepstein *et al*., 2003; Hatsugai *et al*., 2015; Nakaune *et al*., 2005; Sueldo *et al*., 2014).

## Conclusions

Upon agroinfiltration, 25% of the full leaf mRNA transcriptome changes in abundance, associated with an immune response mounted at the expense of photosynthesis. 70% of all extracellular proteins increase in abundance and their predicted functions confirm that an extracellular immune response occurs. Increasing the extracellular proteome while photosynthesis is shut down appears to drive leaves into a nutrient‐deprived state. Engineering *N*. *benthamiana* to react less strongly to Agrobacterium, or Agrobacterium to be less immunogenic in *N. benthamiana*, may enhance RP expression by re‐directing limiting resources. Interestingly, the expression of the silencing inhibitor P19 had minor effects on the transcriptome and no effect on the extracellular proteome.

Discrepancies between changes in transcript, extracellular protein and active extracellular protein abundances suggest that the extracellular proteome is influenced post‐transcriptionally and that many extracellular enzymes are activated post‐translationally. The *N. benthamiana* immune response to agroinfiltration differs from immune responses to bacterial and fungal pathogens in Arabidopsis and tomato in that there is no drastic increase in numbers or amounts of active extracellular subtilases and PLCPs (van Esse *et al*., 2008; Gilroy *et al*., 2007; Sueldo *et al*., 2014; Xia *et al*., 2004). This is surprising, as *N. benthamiana* has an exceptionally large repertoire of 1245 proteases and noncatalytic protease homologs, transcripts corresponding to 975 proteases were detected in leaves and peptides corresponding to 196 proteases were identified in the extracellular space. Prominent features of the extracellular protease repertoire of agroinfiltrated leaves are an expanded clade of SCPLs, highly abundant pepsin‐like proteases and many SBT1 subtilases. Targeted depletion or inhibition of these enzymes may limit undesired proteolysis to improve agroinfiltrated *N. benthamiana* as a protein expression platform. We have selected several proteases for genetic depletion by genome editing to investigate their role in RP degradation and how they shape the endogenous proteome.

## Experimental procedures

All chemicals were obtained from Sigma (Sigma‐Aldrich, St. Louis, MO) unless specified otherwise.

### Agroinfiltration procedure

*Nicotiana benthamiana* plants were grown at 21 °C under a 16/8‐h light/dark regime in a growth room. *Agrobacterium* GV3101‐pMP90 (WT) and *Agrobacterium* GV3101‐pMP90 carrying a plasmid encoding silencing inhibitor P19 of tomato bushy stunt virus, driven by a 35S promoter (pJK050, a gift from Jiorgos Kourelis), were grown for 21 h at 28C with agitation in LB medium (10 g/L NaCl, 10 g/L Tryptone, 5 g/L yeast extract) containing 100 μm rifampicin and 100 μm gentamycin (for WT) plus 100 μm kanamycin (for P19). Bacteria were collected by centrifugation at 2000 g for 5 min at room temperature (RT), resuspended in infiltration buffer (10 mm 2‐(N‐morpholino)ethanesulfone (MES), 10 mm MgCl_2_, pH 5.7, 100 μm acetosyringone) to OD_600_ = 0.5 and left for 2 h at 28 °C with agitation to recover. The first and second fully expanded leaves of preflowering stage *N. benthamiana* (4–5 weeks old) were infiltrated with the bacteria suspension using a syringe without a needle.

### mRNA extraction and sequencing

For each sample, two leaf discs per leaf from six leaves (three different plants) were pulverized under liquid nitrogen using a mortar and pestle. RNA was extracted from 50 mg of leaf powder using TRIZOL (Thermo Fisher Inc, Waltham, MA) according to the manufacturer's instructions. DNA contamination was removed by in‐solution digest with the Qiagen RNAse‐free DNAse kit, followed by cleanup with the Qiagen RNeasy kit, following the manufacturer's instructions (Qiagen, Hilden, Germany). RNA quality was assessed using a Bioanalyzer with the Agilent RNA 6000 Nano Kit (Agilent Technologies, Santa Clara, CA) and following the manufacturer's instructions. All samples used for sequencing had a RIN (RNA integrity number, 28S to 18S rRNA ratio) >6.5. RNAseq library preparation and sequencing were performed by the Wellcome Trust Centre for Human Genetics, Oxford. mRNA was enriched using oligo‐dT beads and sequenced over three lanes of an Illumina HiSeq device, generating on average 184 million 100‐bp paired‐end reads per lane.

### Bioinformatics tools used for transcriptome analysis

To obtain the genome‐based transcriptome (DB4), RNAseq reads were filtered to only retain those with a Phred Q Score >30 (Ewing and Green, 1998) and aligned to the Niben101 genome (Bombarely *et al*., 2012) using TopHat version 2.0.14 (Kim *et al*., 2013a) with default settings. The transcriptome was assembled using StringTie (Pertea *et al*., 2015) on these alignments, allowing for multimapping of reads to several transcripts. TopHat and StringTie were run via the galaxy server (Afgan *et al*., 2016). This resulted in the genome‐based transcriptome (DB4). To obtain the *de novo* assembled transcriptome (DB3), raw reads were quality‐trimmed using TRIMMOMATIC‐0.32 (Bolger *et al*., 2014), BAYESHAMMER (SPADES‐3.5.0) (Nikolenko *et al*., 2013) and ALLPATHS‐LG‐4832 (Butler *et al*., 2008). Ribosomal RNA was removed using SORTMERNA‐1.9 (Kopylova *et al*., 2012). The quality‐trimmed reads were then normalized with a khmer size of 21 in KHMER‐0.7.1 (Crusoe *et al*., 2015). Normalized reads were then assembled and scaffolded using SGA (Simpson and Durbin, 2012), SSPACE‐v.3 (Boetzer *et al*., 2011) and CAP3 (Huang and Madan, 1999). Assembled scaffolds then underwent a final correction step using PILON‐1.6 (Walker *et al*., 2014). This resulted in the *de novo* assembled transcriptome (DB3). We manually curated protease sequences in DB4, using single transcripts from DB1‐3 and 5, as described in Appendix S1. The curated transcriptome was fed to Salmon version 0.7 (Patro *et al*., 2017) together with the filtered reads, and transcript quantification was performed in lightweight alignment mode. Thus, multimapping of reads was allowed during assembly of the transcriptome in DB4, but not during quantification. The NumReads output of Salmon was used for relative expression analysis in DESeq2 (Love *et al*., 2014).

### Bioinformatics tools for proteome prediction

All four transcriptome databases (DB1‐4, see Appendix S1) were subjected to coding sequence prediction using GeneMark‐ST (Tang *et al*., 2015), TransDecoder (http://transdecoder.github.io) and Prodigal (Hyatt *et al*., 2010) using default settings for eukaryotic gene sequences. In cases where all three methods predicted an open reading frame for a transcript the priority was given to the prediction made by GeneMark‐ST unless the GeneMark‐ST gene model was a substring of a longer TransDecoder gene model. Transcripts without predictions by any method were subjected to an additional round of gene prediction using Prodigal settings for bacterial genes and gene predictions were compiled to create the final predicted proteome.

### Apoplastic fluid (AF) extraction

Six *N. benthamiana* leaves per sample were detached and vacuum‐infiltrated with ice‐cold water, dried on the surface and placed in a syringe without needle and plunger that was inserted in a 50‐mL falcon tube. AF was collected by centrifugation at 2000 g, 4 °C for 25 min and stored at ‐80 °C until further use. Protein concentrations were determined with a Bradford assay according to Ernst and Zor (2010). To prove that leakage of cytosolic proteins into the extracellular proteome at later time points upon agroinfiltration is indeed caused by disease and not by our AF extraction method, we measured the activity of the intracellular enzyme malate dehydrogenase (MDH). MDH activity in our AF from mock‐infiltrated leaves falls within the range reported for AF that is virtually free from cytosolic contamination (Figure S4) (Goulet *et al*., 2010; Husted and Schjoerring, 1995).

### Mass spectrometry and ABPP‐MS

see supplemental methods, additional Appendix S7.

### Bioinformatics tools for extracellular proteome analysis

Peptide spectra were annotated using Andromeda (Cox *et al*., 2011). Included modifications were carbamidomethylation (static) and oxidation, N‐terminal acetylation and carbamylation of Lysines and N‐termini (dynamic). Protein quantification was performed using MaxQuant version 1.5.5.30 (Tyanova *et al*., 2016), including all modifications.

### Phylogenetic analyses

Sequences were aligned in Geneious (Kearse *et al*., 2012) using a plug‐in for MAFFT v7.017 (Katoh and Standley, 2013). Neighbour‐joining trees were constructed using the geneious tree builder with Jukes‐Cantor genetic distances and bootstrapped using 1000 times resampling. Trees were edited using iTOL (Letunic and Bork, 2016). Complete versions of the trees including all sequence names are given in Appendix S8.

### Databases and protease annotation

Protease and inhibitor sequences and PFAM annotations were retrieved for Arabidopsis from TAIR10 (Berardini *et al*., 2015) and for rice and tomato from Phytozome (Goodstein *et al*., 2012). Protease sequences from other species to extend the family trees were retrieved from GenBank (NCBI Resource Coordinators, 2017) or UniProt (The UniProt Consortium, 2017). All Arabidopsis, rice, tomato and *N. benthamiana* proteases were annotated by mapping PFAM domains to MEROPS family annotations according to Table S15.

## Data availability

The mass spectrometry proteomics data have been deposited to the ProteomeXchange Consortium via the PRIDE (Vizcaíno *et al*., 2016) partner repository (https://www.ebi.ac.uk/pride/archive/) with the data set identifier PXD006708. RNAseq data have been deposited in the NCBI Sequence Read Archive repository under identifier. [SRP109347]

## Author contributions

R.H. conceived the research. F.G.H. designed and performed experiments and analysed data unless specified otherwise. F.G.H. and R.H interpreted the results. S.K. provided guidance for RNAseq experimental design, transcriptome assembly and differential expression analyses and performed the *de novo* transcriptome assembly and coding sequence prediction. S.B. performed the sample preparation for MS. F.K. and M.K. performed LC‐MS/MS and peptide and protein identification steps for MS of extracellular proteomes and ABPP‐MS. F.G.H wrote the manuscript with feedback from R.H. All authors read and approved the final manuscript.

## Supporting information


**Figure S1** Fold change of transcripts differential between P19 and WT agroinfiltrated leaves.

**Figure S2** Protein concentration in apoplastic fluid over time.

**Figure S3** A phylogenetic tree of proteasome subunits in MEROPS family T01

**Figure S4** Malate dehydrogenase activity in apoplastic fluid.

**Table S1** Differential transcript abundance data, comparing WT and p19 agroinfiltrated leaves

**Table S2** PFAM Domains overrepresented among transcripts differential between WT and P19 agroinfiltrated leaves

**Table S3** Differential protein abundance data, comparing WT and p19 agroinfiltrated leaves

**Table S4** Differential transcript abundance data, comparing agro‐ and mock infiltrated leaves

**Table S5** PFAM Domains overrepresented among transcripts differential between agro‐ and mock infiltrated leaves

**Table S6** Differential protein abundance data, comparing agro‐ and mock infiltrated leaves

**Table S7** PFAM Domains overrepresented among proteins differential between agro‐ and mock infiltrated leaves

**Table S8** Agrobacterium proteins for which corresponding peptides were identified in the extracellular proteome

**Table S9** Differential transcript abundance data over time

**Table S10** Differential protein abundance data over time

**Table S11** Data from ABPP‐MS analyses

**Table S12** Discrepancies between changes in extracellular protein and transcript levels

**Table S13** Discrepancies between changes in extracellular activity and extracellular protein levels

**Table S14** Protease family sizes in Arabidopsis, tomato, rice and *N. benthamiana*

**Table S15** PFAM Families mapped to MEROPS families

**Appendix S1** Detailing curation of the proteome database

**Appendix S2** R code used for RNAseq data analysis

**Appendix S3** R code used for extracellular proteome data analysis

**Appendix S4** R code used for analysis of the effects of leaf ageing

**Appendix S5** R code used for ABPP‐MS data analysis

**Appendix S6** R code used for analysis of discrepancies between changes in extracellular activity, extracellular protein abundance and transcript abundance

**Appendix S7** Supplemental methods used for mass spectrometry sample preparation

**Appendix S8** Full versions of the trees shown in Figures 5 and 6 with all gene names
