# Supplementary material for: The transcriptome, extracellular proteome and active secretome of agroinfiltrated Nicotiana benthamiana uncover a large, diverse protease repertoire
Source: Plant Biotechnol J. 2017 Dec 17;16(5):1068–84. doi: 10.1111/pbi.12852 (PMC5902771; doi:10.1111/pbi.12852)
Supplement: Supplementary file 1 — Figure S1 Fold change of transcripts differential between P19 and WT agroinfiltrated leaves. [file PBI-16-1068-s001.pdf]

Figure S01: Transcripts with differential abundance between p19 and WT agroinfiltrated leaves

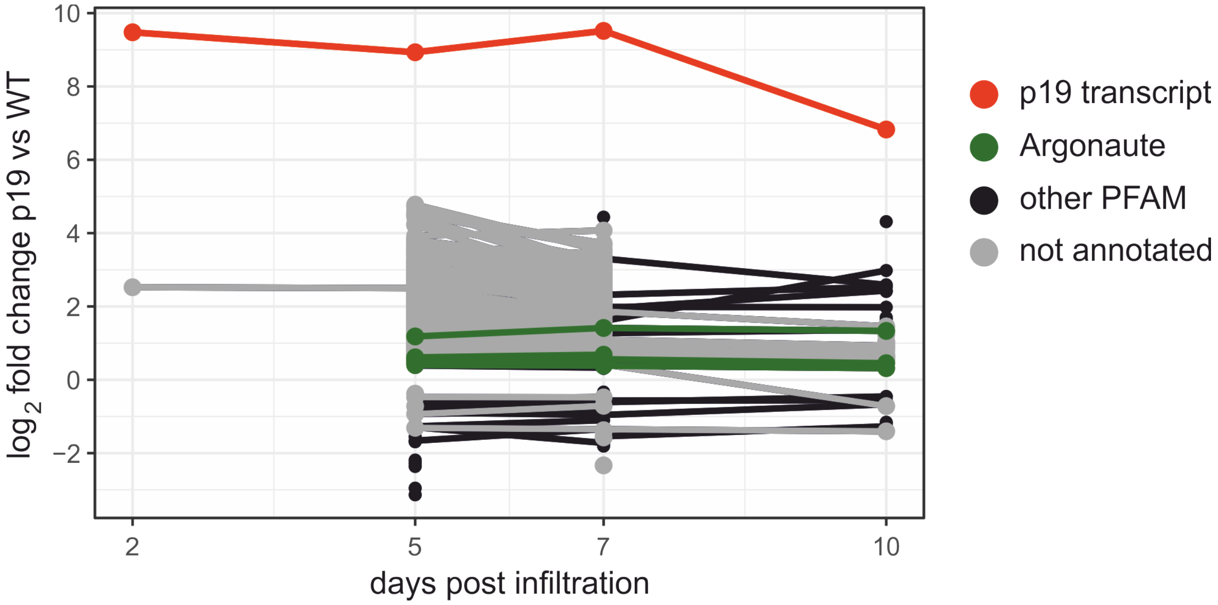

Testing for differential expression was performed as implemented in DESeq, using a Wald-Test and filtering for Benjamini-Hochberg adjusted p-value < 0.05. Only differentially abundant transcripts are shown.
