# Supplementary material for: The transcriptome, extracellular proteome and active secretome of agroinfiltrated Nicotiana benthamiana uncover a large, diverse protease repertoire
Source: Plant Biotechnol J. 2017 Dec 17;16(5):1068–84. doi: 10.1111/pbi.12852 (PMC5902771; doi:10.1111/pbi.12852)
Supplement: Supplementary file 2 — Figure S2 Protein concentration in apoplastic fluid over time. [file PBI-16-1068-s025.pdf]

Figure S10: protein concentration in apoplastic fluid samples

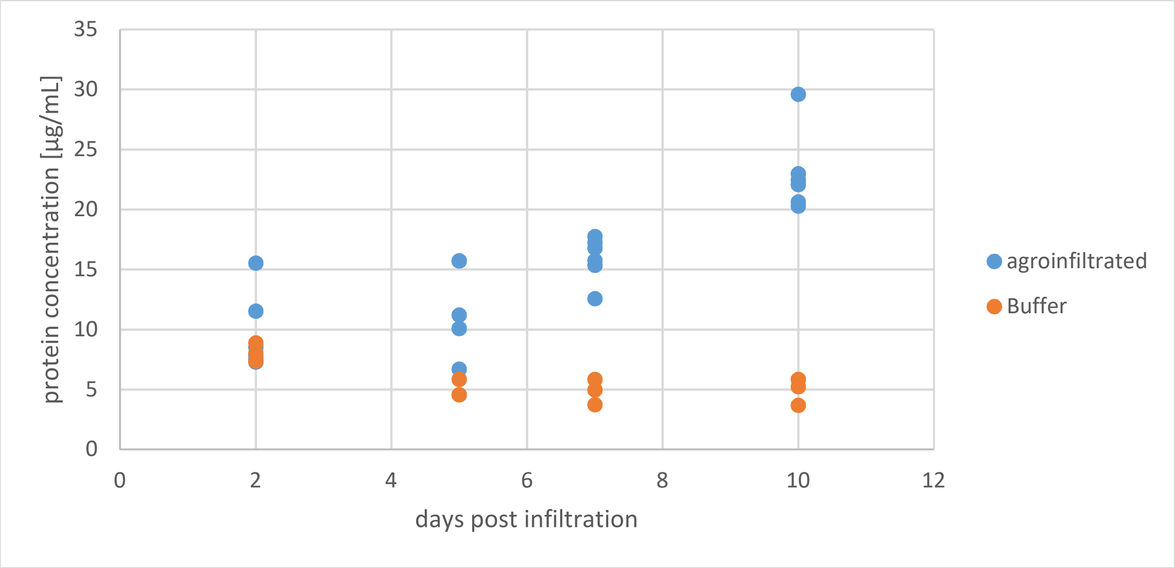

Protein concentrations were determined using a microtiter-plate based Bradford assay according to (Ernst & Zor, 2010). Values are given for each biological replicate as a mean of three dilutions measured on the same plate.

Ernst, O. & Zor, T. (2010) Linearization of the Bradford protein assay. *J. Vis. Exp. JoVE*,.
