# Supplementary figures and images for: The transcriptome, extracellular proteome and active secretome of agroinfiltrated Nicotiana benthamiana uncover a large, diverse protease repertoire
Source: Plant Biotechnol J. 2017 Dec 17;16(5):1068–84. doi: 10.1111/pbi.12852 (PMC5902771; doi:10.1111/pbi.12852)

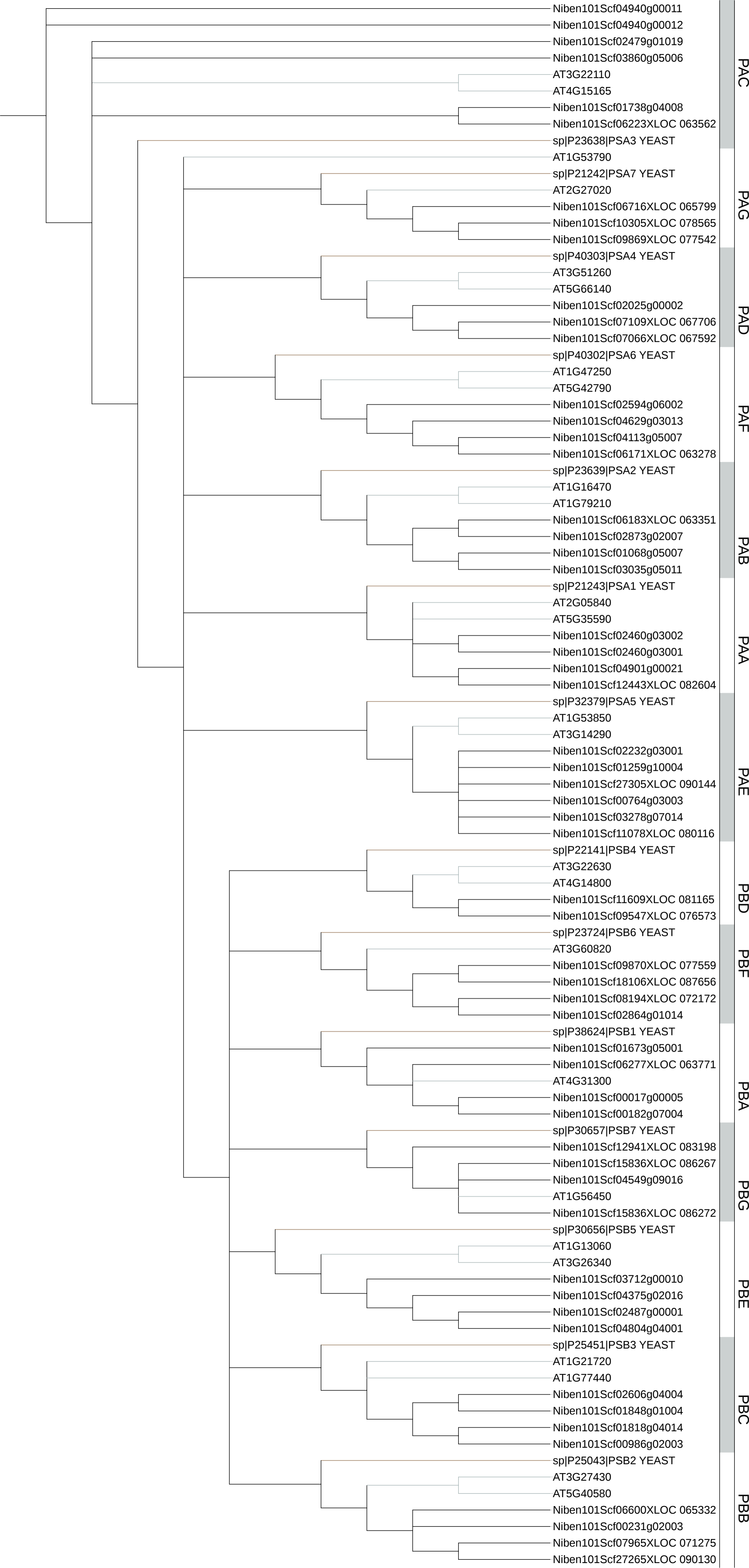

Supplement: Supplementary file 3 — Figure S3 A phylogenetic tree of proteasome subunits in MEROPS family T01 [file PBI-16-1068-s024.pdf]

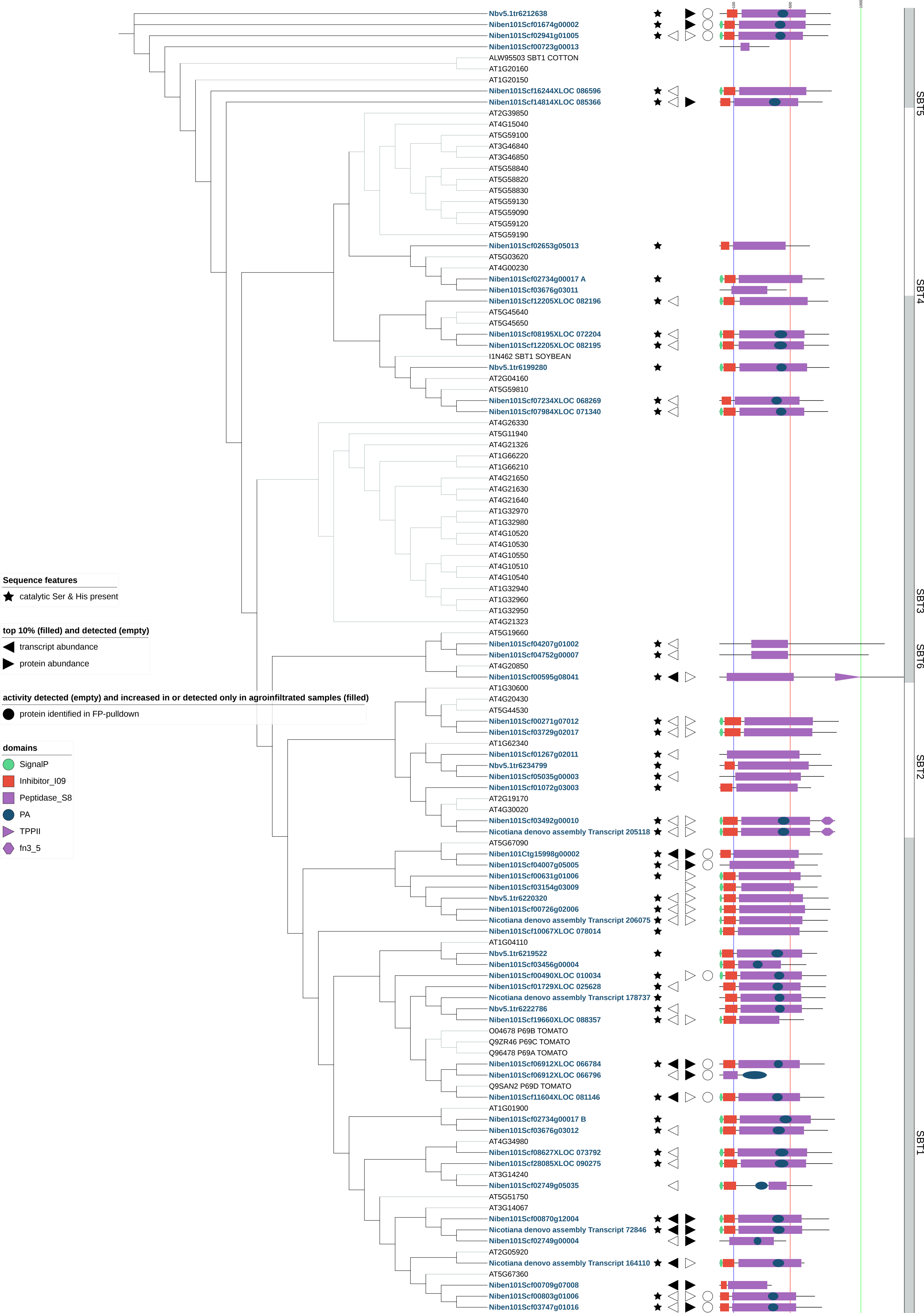

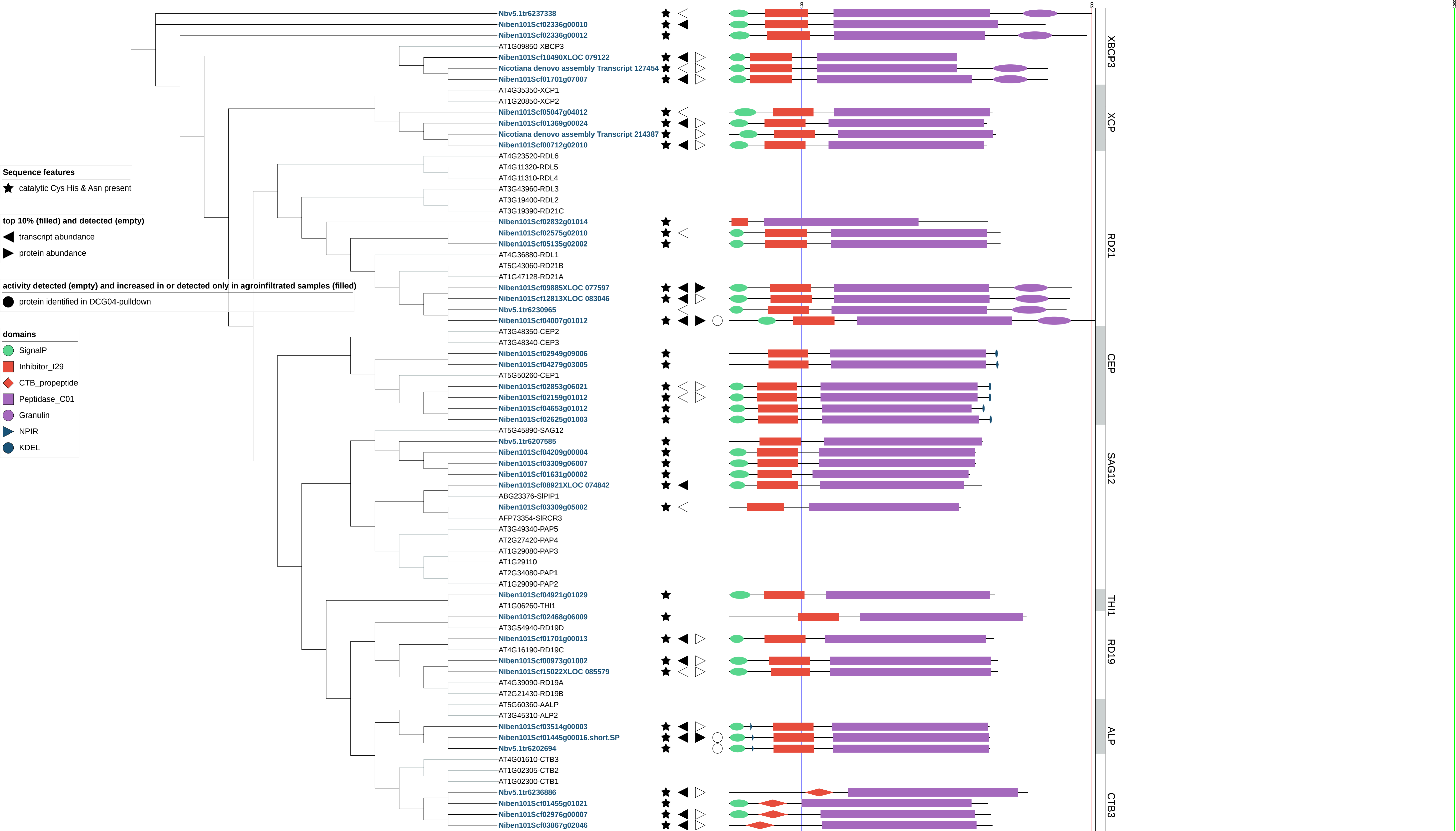

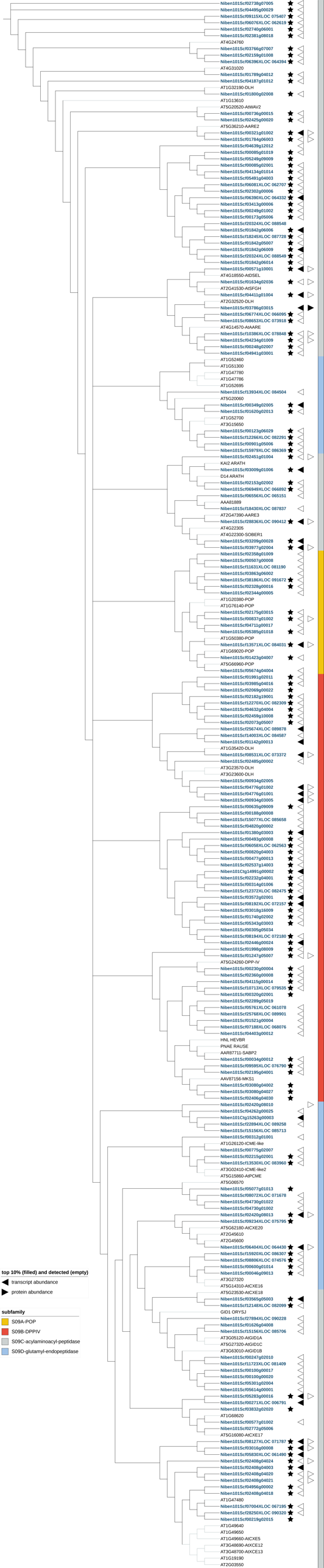

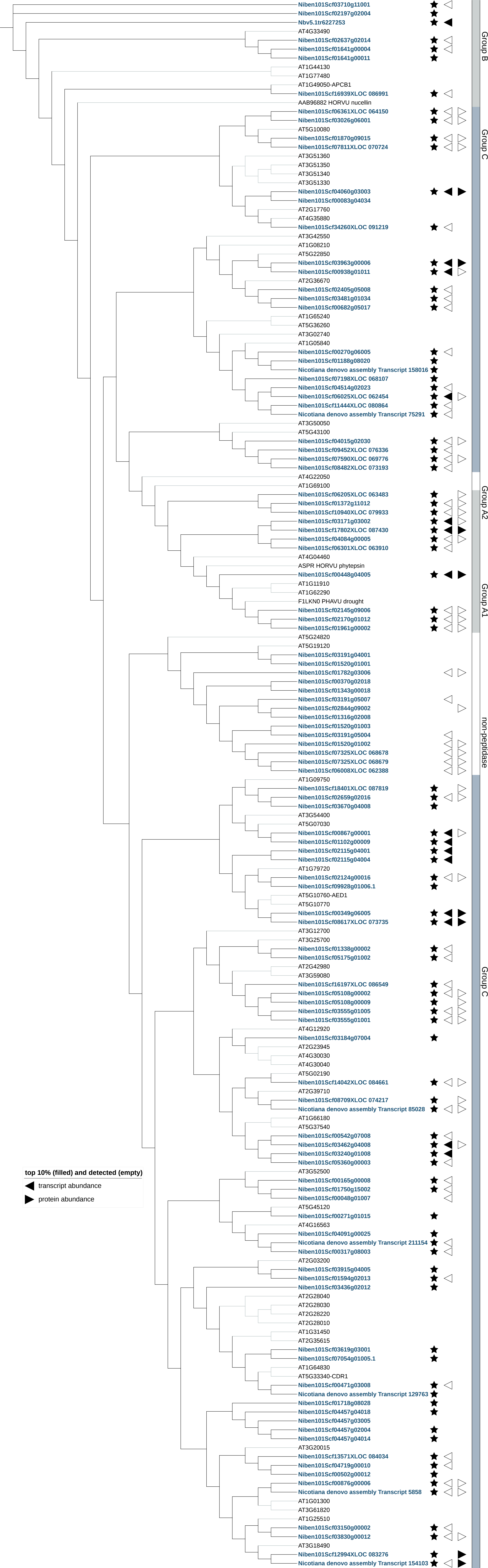

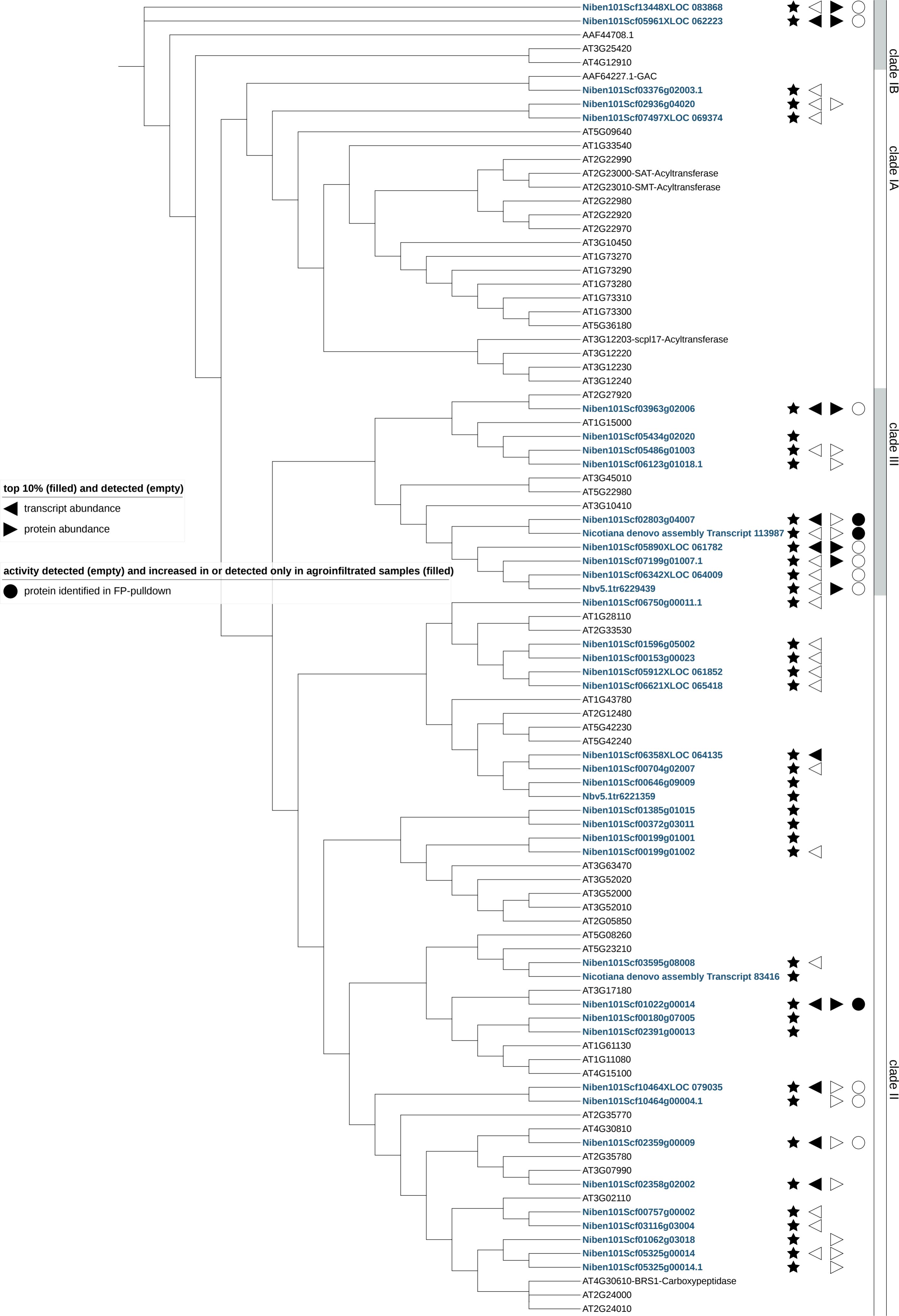

top 10% (filled) and detected (empty)

◀ transcript abundance

▶ protein abundance

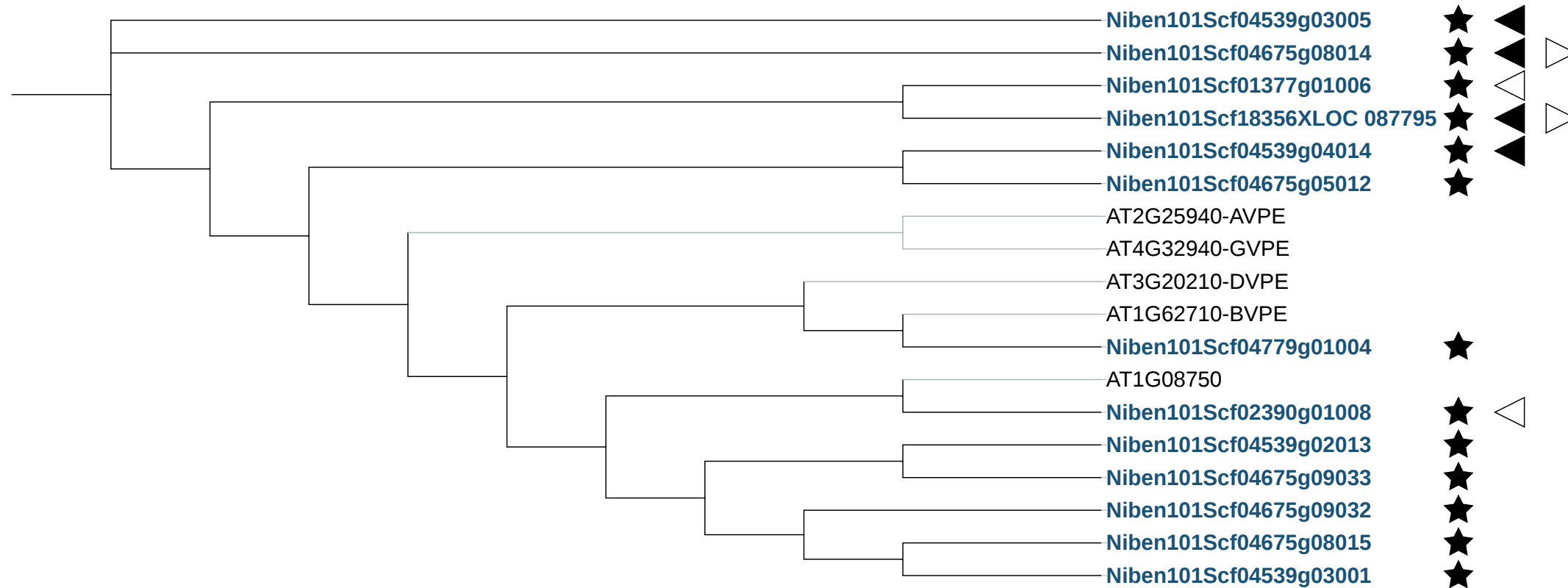

Supplement: Supplementary file 27 — Appendix S8 Full versions of the trees shown in Figures 5 and 6 with all gene names [file PBI-16-1068-s023.pdf]
