# Supplementary material for: The transcriptome, extracellular proteome and active secretome of agroinfiltrated Nicotiana benthamiana uncover a large, diverse protease repertoire
Source: Plant Biotechnol J. 2017 Dec 17;16(5):1068–84. doi: 10.1111/pbi.12852 (PMC5902771; doi:10.1111/pbi.12852)
Supplement: Supplementary file 4 — Figure S4 Malate dehydrogenase activity in apoplastic fluid. [file PBI-16-1068-s026.pdf]

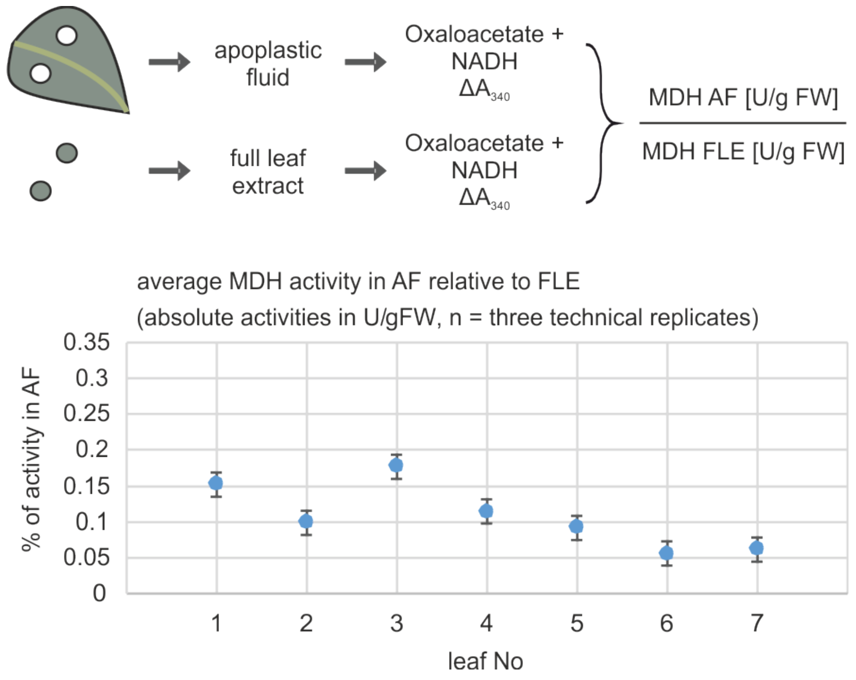

Figure S04: Malate dehydrogenase enzyme activity assay (MDH assay). *N. benthamiana* plants were infiltrated with infiltration buffer (mock treatment) and harvested at 2 dpi. One *N. benthamiana* leaf per sample was detached, a full leaf extract (FLE) was prepared from two leaf discs and apoplastic fluid (AF) extracted from the remainder of the leaf. MDH activity in U/g fresh weight (FW) was compared between AF and FLE from the same leaf for each sample. FLE was prepared by grinding frozen tissue using metal beads and a TissueLyser (Quiagen, Hilden, DE), mixing the tissue powder with 133 µl ice-cold PBS and separating the FLE from tissue and beads by centrifugation at 4 °C, 13000 g, for 10 min. Protein concentrations of FLE and AF were determined using a Bradford assay (Ernst & Zor, 2010) and FLE was diluted to meet the protein concentration of the corresponding AF. MDH activity was measured in 50 mM Tris-HCL, pH 7.5, 0.8 mM oxaloacetate and 0.4 mM NADH, using 20 µl AF or diluted FLE for each 200 µl reaction. The reduction in absorbance at 340 nm was read every 17 seconds using a microplate reader (Tecan Group Ltd., Maennedorf, CH) and the slope used to calculate the MDH activity according to Lambert-Beers law. Mean and standard deviation of three technical replicates per sample are shown. The assay was repeated twice with similar results.

Ernst, O. & Zor, T. (2010) Linearization of the Bradford protein assay. *J. Vis. Exp. JoVE*,.
