# Supplementary material for: The transcriptome, extracellular proteome and active secretome of agroinfiltrated Nicotiana benthamiana uncover a large, diverse protease repertoire
Source: Plant Biotechnol J. 2017 Dec 17;16(5):1068–84. doi: 10.1111/pbi.12852 (PMC5902771; doi:10.1111/pbi.12852)
Supplement: Supplementary file 20 — Appendix S1 Detailing curation of the proteome database [file PBI-16-1068-s017.pdf]

A curated proteome database for apoplastic proteome analysis

During a first annotation of the transcriptome and proteome data, we observed that well-known proteases including PLCPs (MEROPS family C01) and subtilases (S08) often appeared truncated or lacked conserved domains in the Niben101 proteome database (https://solgenomics.net/). To improve the protease annotation, we evaluated the predicted proteome databases from four transcriptomes: the Niben101 transcriptome as sequenced by a consortium at the Boyce Thompson institute (https://solgenomics.net/) (DB1); the Nbv5 transcriptome as sequenced by a consortium based at the University of Sydney (Nakasugi *et al.*, 2014) (DB2); a *de novo* assembly of our own RNAseq reads (DB3) and a transcriptome generated by mapping our RNAseq reads to the Niben101 genome (Bombarely *et al.*, 2012) (DB4). We compared the predicted protein databases (DB1-4) using three different metrics.

First, we analysed protein length as a proxy for protein completeness. Protein length distributions of DB1-4 showed that our *de novo* assembly (DB3) contains a large proportion of short, probably incomplete sequences, with a median protein length of 156 amino acids (AA). This is an inherent weakness of *de novo* assemblies, which cannot draw on the genome sequence at sites with insufficient overlap between RNAseq reads. The Nbv5 transcriptome (DB2) was more carefully assembled and has a median protein length of 180 AA, but DB2 is the smallest of the four databases. DB4 has a median protein length of 172 AA and thus has a higher proportion of short sequences than DB1 (median protein length 261 AA), but DB4 is substantially larger than any of the other three databases (Figure S19.1a).

Second, to assess which of the databases contains most complete plant proteins, we determined Best Hit Ratios (BHRs) between DB1-4 and the Arabidopsis TAIR10 database (Berardini *et al.*, 2015). The BHR is the number of matched positions between a *N. benthamiana* query sequence and its best blast hit in the Arabidopsis proteome divided by the length of the hit (O'Neil & Emrich, 2013). Histograms of the BHR values show that the genome-based database (DB4) has the highest number of complete best hits (BHR close to 1) (Figure S19.1b).

Third, we annotated MS/MS spectra from shotgun proteomics of 36 AF samples with each of the four databases. DB4 performed best in this analysis, closely followed by the proteome predicted from the Niben101 transcriptome (DB1) (Figure S19.1c).

We continued with our genome-based transcriptome assembly (DB4) as a starting point, because it is the largest database, has a large proportion of long sequences as well as the highest number of complete best blast hits (BHR = 1) and matched the MS data best. Protease families, however, were still poorly annotated in DB4. We therefore followed a manual curation pipeline to correct proteases

in six protease families: A01, C01, C13, S08, S09 and S10. We collected the proteases from the four *N. benthamiana* proteomes and Arabidopsis TAIR10 using PFAM 30.0 (Finn *et al.*, 2016) and PFAM to MEROPS mapping (Supplementary Table S1). We generated phylogenetic trees from DB4 and TAIR10 for each protease family and used the underlying alignment to identify 197 incomplete (lacking the catalytic site or crucial motifs) and truncated sequences in DB4, corresponding to 53 % of the proteases in the six families in DB4. We replaced 64 incomplete sequences with longer versions from DB1-4. By allowing for a replacement from DB4, we ensured that duplicated sequences were collapsed into the longest available version. We retained 74 sequences that lacked the catalytic site but had no complete version in any of the four databases. We discarded 59 sequences that were truncated and had no longer version in any of the four databases (Figure S19.2a). This resulted in a curated *N. benthamiana* proteome database DB5.

Searching the apoplastic proteome MS spectra with DB5 allowed us to detect 30 proteins more in the apoplast than with the genome-based database DB4, showing that the considerable effort of curating is warranted. We also identified new peptides in the MS data (i.e Figures S19.2b and S19.2d) and thus increased the confidence in the protein identification, reflected in a higher protein score (Tyanova *et al.*, 2016). Identification of new peptides with the curated database can lead to assignment of additional peptides to a protein because of the “winner takes it all”/razor principle used by the Andromeda search engine in MaxQuant (Tyanova *et al.*, 2016) (i.e. Figures S19.2b and S19.2d). In 151 cases, this allows us to detect proteins we were unable to identify before, either because they were not part of DB4 or because they had no peptides assigned to them (i.e. Figure S19.2c).

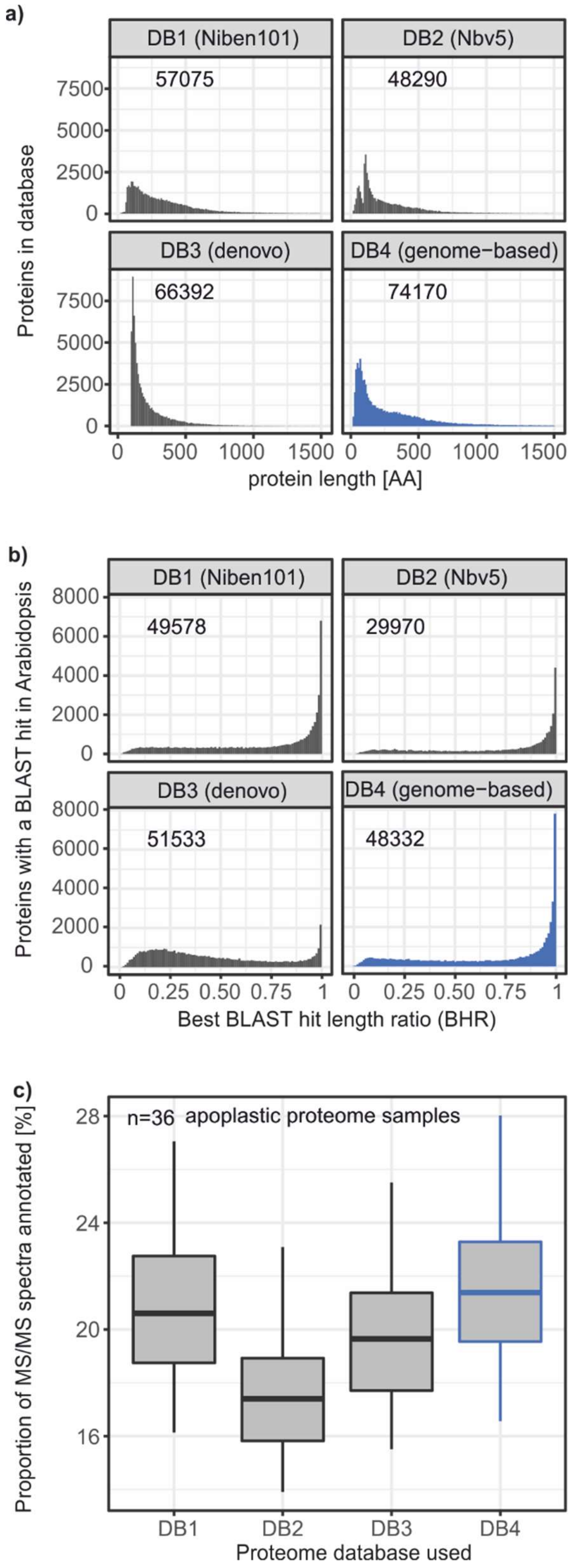

Figure S19.1: Four *N. benthamiana* proteome databases

Comparison of four *N. benthamiana* proteomes, predicted from the Niben101 transcriptome (DB1), the Nbv5 transcriptome (DB2), a *de novo* assembly of the RNAseq reads obtained in this study (DB3) and a genome-based assembly of the RNAseq reads obtained in this study using the Niben101 genome (DB4). a) Histograms of protein length values in the four databases and total number of sequences in each database. b) Histograms of best BLAST hit length ratio (BHR) values for the four databases and the total number of proteins with a BLAST hit in Arabidopsis in each database. c) Boxplots giving the proportion of MS spectra annotated with each database, with the upper and lower hinges corresponding to 25th and 75th percentiles, the middle line to the median and the whiskers to the lowest and highest data points within 1.5 interquartile ranges from the hinge.

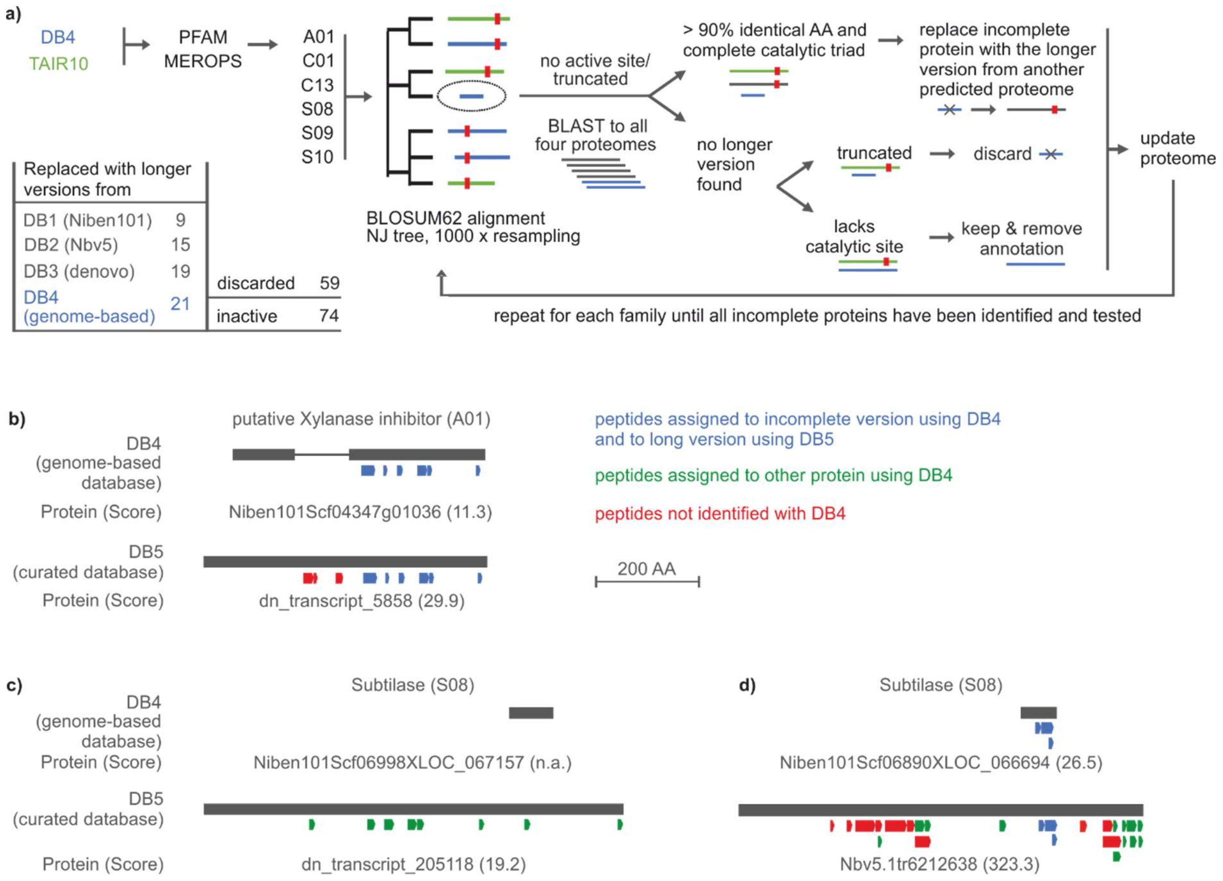

Figure S19.2: Manual curation of protease families

a) Flowchart detailing our manual annotation approach. b-d: Examples of proteins that could be identified with a higher confidence (higher protein score) using DB5 (curated proteome) compared to DB4 (proteome predicted from alignment of RNAseq reads to the Niben101 genome). Thick grey bars indicate protein lengths; thin grey bars indicate gaps in the alignment of the incomplete protein with its respective complete version. NJ, neighbour-joining; n.a. not applicable, as the protein was not detected.

Berardini, T.Z., Reiser, L., Li, D., Mezheritsky, Y., Muller, R., Strait, E., & Huala, E. (2015) The arabidopsis information resource: Making and mining the "gold standard" annotated reference plant genome. *genesis*, **53**, 474–485.

Bombarely, A., Rosli, H.G., Vrebalov, J., Moffett, P., Mueller, L.A., & Martin, G.B. (2012) A draft genome sequence of Nicotiana benthamiana to enhance molecular plant-microbe biology research. *Mol. Plant. Microbe Interact.*, **25**, 1523–1530.

Finn, R.D., Coggill, P., Eberhardt, R.Y., Eddy, S.R., Mistry, J., Mitchell, A.L., Potter, S.C., Punta, M., Qureshi, M., Sangrador-Vegas, A., Salazar, G.A., Tate, J., & Bateman, A. (2016) The Pfam protein families database: towards a more sustainable future. *Nucleic Acids Res.*, **44**, D279–D285.

Nakasugi, K., Crowhurst, R., Bally, J., & Waterhouse, P. (2014) Combining transcriptome assemblies from multiple de novo assemblers in the allo-tetraploid plant Nicotiana benthamiana. *PLOS ONE*, **9**, e91776.

O'Neil, S.T. & Emrich, S.J. (2013) Assessing De Novo transcriptome assembly metrics for consistency and utility. *BMC Genomics*, **14**, 465.

Tyanova, S., Temu, T., & Cox, J. (2016) The MaxQuant computational platform for mass spectrometry-based shotgun proteomics. *Nat. Protoc.*, **11**, 2301–2319.
