# Supplementary material for: The transcriptome, extracellular proteome and active secretome of agroinfiltrated Nicotiana benthamiana uncover a large, diverse protease repertoire
Source: Plant Biotechnol J. 2017 Dec 17;16(5):1068–84. doi: 10.1111/pbi.12852 (PMC5902771; doi:10.1111/pbi.12852)
Supplement: Supplementary file 21 — Appendix S2 R code used for RNAseq data analysis [file PBI-16-1068-s018.html]

Statistical analysis of transcriptome data


# Statistical analysis of transcriptome data

#### *FGH*

#### *24 January 2017*

Steps:

- Using the NumReads output of Salmon for each sample, make a heatmap reflecting the Euclidean distances between the whole transcriptomes (shown in Figure 6b)
- Calculate day-by-day t-tests with BH correction for Agro vs Buffer
- Group transcripts by when their abundance first changes significantly and more than two-fold in response to agroinfiltration
- Annotate the transcripts and test for enrichment of pfam annotations in each regulatory group vs all expressed transcripts.
- Plot the sizes of the regulatory groups for Figure 6c

### Euclidean distance clustering of transcriptomes (Figure 6b)

```
library(DESeq2)
```

```
## Warning: package 'Rcpp' was built under R version 3.2.5
```

```
## Warning: package 'RcppArmadillo' was built under R version 3.2.5
```

```
library(data.table)
# reading in & cosmetics
count.data.r <- read.csv("analysis\\count.data.salmonv0.7.csv", row.names = 1, 
    strip.white = T)  #This file contains the NumReads output of salmon
count.data.r$sumcounts <- NULL
col.data.noB5W.AgrovsBuf <- read.csv("raw.data\\col.data.noB5W.WTvsP19vsBuf.csv", 
    row.names = 1, strip.white = T)
names(col.data.noB5W.AgrovsBuf) <- c("treatment", "dpi")
col.data.noB5W.AgrovsBuf$treatment <- factor(col.data.noB5W.AgrovsBuf$treatment, 
    levels = c("Buffer", "Agro.WT", "Agro.p19"))
col.data.noB5W.AgrovsBuf$dpi <- factor(col.data.noB5W.AgrovsBuf$dpi, levels = c("2", 
    "5", "7", "10"))

# calculate distances
dds.AgrovsBuf <- DESeqDataSetFromMatrix(countData = count.data.r, colData = col.data.noB5W.AgrovsBuf, 
    design = ~dpi + treatment)
dds.AgrovsBuf.rld <- rlog(dds.AgrovsBuf)
RNAseq.distsRL <- dist(t(assay(dds.AgrovsBuf.rld)))
# cluster by behaviour
RNAseq.hc <- hclust(RNAseq.distsRL)
RNAseq.dd <- as.dendrogram(RNAseq.hc)
# cosmetics for plotting
library(dendextend)
```

```
## Warning: package 'dendextend' was built under R version 3.2.5
```

```
## Warning: replacing previous import by 'magrittr::%>%' when loading
## 'dendextend'
```

```
RNAseq.dd.reorder <- rotate(RNAseq.dd, c("A2B", "B2B", "C2B", "A5B", "B5B", 
    "C5B", "A7B", "B7B", "C7B", "A10B", "B10B", "C10B", "A2W", "B2W", "C2W", 
    "A2P", "B2P", "C2P", "A5W", "C5W", "A5P", "B5P", "C5P", "A7W", "B7W", "C7W", 
    "A7P", "B7P", "C7P", "A10W", "B10W", "C10W", "A10P", "B10P", "C10P"))
RNAseq.mat <- as.matrix(RNAseq.distsRL)
rownames(RNAseq.mat) <- colnames(RNAseq.mat) <- with(colData(dds.AgrovsBuf), 
    paste(treatment, dpi, sep = ", "))
# plotting the heatmap
library(RColorBrewer)
newcol <- colorRampPalette(brewer.pal(9, "Blues"))
ncols <- 50
bluecols2 <- newcol(ncols)
library(gplots)
```

```
## Warning: package 'gplots' was built under R version 3.2.5
```

```
heatmap.2(RNAseq.mat, Rowv = RNAseq.dd.reorder, symm = T, trace = "none", main = "Euclidean sample distances\nfor transcriptomes of agroinfiltrated leaves", 
    density.info = "none", col = rev(bluecols2))
```

### Statistical analysis of transcript abundance in agro- (incl p19) vs buffer infiltrated leaves at each time point

```
library(data.table)
At.RNA <- data.table(read.csv("analysis\\count.data.salmonv0.7.csv", stringsAsFactors = F, strip.white = T))#This file contains the NumReads output of salmon
At.RNA <- At.RNA[ID %like% "Niben" |ID %like% "Nbv" |ID %like% "Nicotiana" |
                   ID %like% "p19", ]# only looking @ the plant

library(DESeq2)
col.data.noB5W.AgrovsBuf <- read.csv("raw.data\\col.data.noB5W.AgrovsBuf.csv", row.names = 1)
names(col.data.noB5W.AgrovsBuf) <- c("treatment", "dpi")
col.data.noB5W.AgrovsBuf$treatment <- factor(col.data.noB5W.AgrovsBuf$treatment, levels = c("Buffer", "Agro"))
col.data.noB5W.AgrovsBuf$dpi <- factor(col.data.noB5W.AgrovsBuf$dpi, levels = c("2", "5", "7", "10"))

At.RNA.NumReads <- data.frame(At.RNA[, c(2:36), with=F], row.names = At.RNA[ , ID])

dds.At.RNA <- DESeqDataSetFromMatrix(countData = At.RNA.NumReads, colData = col.data.noB5W.AgrovsBuf, design = ~ 1)
dds.At.RNA$group <- factor(paste0(dds.At.RNA$treatment, dds.At.RNA$dpi))
design(dds.At.RNA) <- ~ group

#size factors (to account for differences in sequencing depth) are only based on transcripts with >3500 NumReads.
#By visual inspection of the density of NumRead values in different samples before and after normalization,
#this was determined to be the optimal treshold. 
count.data.forsizeFactors <- At.RNA.NumReads
count.data.forsizeFactors <- data.table(count.data.forsizeFactors)
count.data.forsizeFactors <- count.data.forsizeFactors[, sumcounts := Reduce(`+`, .SD)]
count.data.forsizeFactors.100 <- count.data.forsizeFactors[which(sumcounts>3500),]
count.data.forsizeFactors <- data.frame(count.data.forsizeFactors.100)
count.data.forsizeFactors$sumcounts <- NULL
dds.forsizefactors <- DESeqDataSetFromMatrix(count.data.forsizeFactors, col.data.noB5W.AgrovsBuf, design = ~ 1)
dds.forsizefactors$group <- factor(paste0(dds.At.RNA$treatment, dds.At.RNA$dpi))
design(dds.forsizefactors) <-  ~ group
dds.forsizefactors <- estimateSizeFactors(dds.forsizefactors)

sizeFactors(dds.At.RNA) <- sizeFactors(dds.forsizefactors)
dds.At.RNA <- estimateDispersions(dds.At.RNA)
dds.At.RNA <- nbinomWaldTest(dds.At.RNA, betaPrior = T)

#get the lfcs & padj (BH, as per default) for each timepoint
results.At.RNA <- results(dds.At.RNA, alpha = 0.05,
                          cooksCutoff = F,#turn outlier filtering off 
                          contrast = c("group", "Agro2", "Buffer2"))
resdpi <- data.frame(results.At.RNA)
resdpi[ , "ID"] <- row.names(resdpi)
resdpi <- data.table(resdpi)
resdpi[ , timepoint := "2dpi"]

results.At.RNA.5 <- results(dds.At.RNA, alpha = 0.05,
                          cooksCutoff = F,#turn outlier filtering off 
                          contrast = c("group", "Agro5", "Buffer5"))
res.5dpi <- data.frame(results.At.RNA.5)
res.5dpi[ , "ID"] <- row.names(res.5dpi)
res.5dpi <- data.table(res.5dpi)
res.5dpi[ , timepoint := "5dpi"]

results.At.RNA.7 <- results(dds.At.RNA, alpha = 0.05,
                          cooksCutoff = F,#turn outlier filtering off 
                          contrast = c("group", "Agro7", "Buffer7"))
res.7dpi <- data.frame(results.At.RNA.7)
res.7dpi[ , "ID"] <- row.names(res.7dpi)
res.7dpi <- data.table(res.7dpi)
res.7dpi[ , timepoint := "7dpi"]

results.At.RNA.10 <- results(dds.At.RNA, alpha = 0.05,
                          cooksCutoff = F,#turn outlier filtering off 
                          contrast = c("group", "Agro10", "Buffer10"))
res.10dpi <- data.frame(results.At.RNA.10)
res.10dpi[ , "ID"] <- row.names(res.10dpi)
res.10dpi <- data.table(res.10dpi)
res.10dpi[ , timepoint := "10dpi"]

res.all <- rbind(resdpi, res.5dpi, res.7dpi, res.10dpi)#results in long format.
res.all.w <- dcast(res.all, ID + baseMean ~ timepoint, value.var = c("log2FoldChange", "padj"))#results in wide format
```

### Dynamics categorization

Categorize by when a transcript is first regulated significantly and more than 2-fold (2, 5, 7 or 10 dpi) and whether that regulation is up or down.

```
# name up categories
res.all.w[log2FoldChange_2dpi > 1 & padj_2dpi < 0.05, `:=`(dynamics, "1st up 2 dpi")]

res.all.w[(log2FoldChange_5dpi > 1 & padj_5dpi < 0.05) & !(abs(log2FoldChange_2dpi) > 
    1 & padj_2dpi < 0.05), `:=`(dynamics, "1st up 5 dpi")]

res.all.w[(log2FoldChange_7dpi > 1 & padj_7dpi < 0.05) & !((abs(log2FoldChange_2dpi) > 
    1 & padj_2dpi < 0.05) | (abs(log2FoldChange_5dpi) > 1 & padj_5dpi < 0.05)), 
    `:=`(dynamics, "1st up 7 dpi")]

res.all.w[(log2FoldChange_10dpi > 1 & padj_10dpi < 0.05) & !((abs(log2FoldChange_2dpi) > 
    1 & padj_2dpi < 0.05) | (abs(log2FoldChange_5dpi) > 1 & padj_5dpi < 0.05) | 
    abs(log2FoldChange_7dpi) > 1 & padj_7dpi < 0.05), `:=`(dynamics, "1st up 10 dpi")]

# name down categories
res.all.w[log2FoldChange_2dpi < (-1) & padj_2dpi < 0.05, `:=`(dynamics, "1st down 2 dpi")]

res.all.w[(log2FoldChange_5dpi < (-1) & padj_5dpi < 0.05) & !(abs(log2FoldChange_2dpi) > 
    1 & padj_2dpi < 0.05), `:=`(dynamics, "1st down 5 dpi")]

res.all.w[(log2FoldChange_7dpi < (-1) & padj_7dpi < 0.05) & !((abs(log2FoldChange_2dpi) > 
    1 & padj_2dpi < 0.05) | (abs(log2FoldChange_5dpi) > 1 & padj_5dpi < 0.05)), 
    `:=`(dynamics, "1st down 7 dpi")]

res.all.w[(log2FoldChange_10dpi < (-1) & padj_10dpi < 0.05) & !((abs(log2FoldChange_2dpi) > 
    1 & padj_2dpi < 0.05) | (abs(log2FoldChange_5dpi) > 1 & padj_5dpi < 0.05) | 
    abs(log2FoldChange_7dpi) > 1 & padj_7dpi < 0.05), `:=`(dynamics, "1st down 10 dpi")]

# name constant
res.all.w[!((abs(log2FoldChange_2dpi) > 1 & padj_2dpi < 0.05) | (abs(log2FoldChange_5dpi) > 
    1 & padj_5dpi < 0.05) | (abs(log2FoldChange_7dpi) > 1 & padj_7dpi < 0.05) | 
    (abs(log2FoldChange_10dpi) > 1 & padj_10dpi < 0.05)), `:=`(dynamics, "constant")]

res.all.w[, .N, by = "dynamics"]
```

### Annotation and pfam family enrichment analysis

```
annot <- data.table(read.csv("raw.data\\annot.curated.csv", stringsAsFactors = F))
res.all.annot <- merge(res.all.w, annot, by="ID", all.x=T)
write.csv(res.all.annot, "analysis//AtRNA.1stat.curatedDB.res.all.annot.csv", row.names = F)#This is Table S4

res.all.annot <- data.table(read.csv("analysis//AtRNA.1stat.curatedDB.res.all.annot.csv", stringsAsFactors = F))

pfams.all <- res.all.annot[, .(pfam.No=unlist(strsplit(pfam.No, ";")), dynamics), by="ID"]
pfams.all.c <- pfams.all[ , .(N.all = .N), by="pfam.No"]#get all pfams in txome and how often they are represented

#Count how often each pfam turns up in each dynamic category and whether that's significant enrichment
pfamsdpi.c <- pfams.all[dynamics=="1st up 2 dpi", .N, by="pfam.No"]#count 2dpi
pfamsdpi.c <- merge(pfamsdpi.c, pfams.all.c[ , .(N.all, pfam.No)], by="pfam.No", all.x=T)#get NumReads in all
pfamsdpi.c[, p.overrep.up2 :=
             phyper(N,#see help->phyper. We are testing whether finding this pfam N or more times is significantly different than what you would expect if 2dpiup were a random subset of the txome.
                    N.all,
                    sum(pfams.all.c$N)-N.all,
                    sum(pfamsdpi.c$N), lower.tail = F),
           by="pfam.No"]

pfams.5dpi.c <- pfams.all[dynamics=="1st up 5 dpi", .N, by="pfam.No"]
pfams.5dpi.c <- merge(pfams.5dpi.c, pfams.all.c[ , .(N.all, pfam.No)], by="pfam.No", all.x=T)
pfams.5dpi.c[, p.overrep.up5 :=
             phyper(N,
                    N.all,
                    sum(pfams.all.c$N)-N.all,
                    sum(pfams.5dpi.c$N), lower.tail = F),
           by="pfam.No"]

pfams.7dpi.c <- pfams.all[dynamics=="1st up 7 dpi", .N, by="pfam.No"]
pfams.7dpi.c <- merge(pfams.7dpi.c, pfams.all.c[ , .(N.all, pfam.No)], by="pfam.No", all.x=T)
pfams.7dpi.c[, p.overrep.up7 :=
             phyper(N,
                    N.all,
                    sum(pfams.all.c$N)-N.all,
                    sum(pfams.7dpi.c$N), lower.tail = F),
           by="pfam.No"]

pfams.10dpi.c <- pfams.all[dynamics=="1st up 10 dpi", .N, by="pfam.No"]
pfams.10dpi.c <- merge(pfams.10dpi.c, pfams.all.c[ , .(N.all, pfam.No)], by="pfam.No", all.x=T)
pfams.10dpi.c[, p.overrep.up10 :=
             phyper(N,
                    N.all,
                    sum(pfams.all.c$N)-N.all,
                    sum(pfams.10dpi.c$N), lower.tail = F),
           by="pfam.No"]

#count down categories
pfamsdpi.cd <- pfams.all[dynamics=="1st down 2 dpi", .N, by="pfam.No"]#count 2dpi
pfamsdpi.cd <- merge(pfamsdpi.cd, pfams.all.c[ , .(N.all, pfam.No)], by="pfam.No", all.x=T)#get NumReads in all
pfamsdpi.cd[, p.overrep.down2 :=
             phyper(N,#see help->phyper. We are testing whether finding this pfam N or more times is significantly different than what you would expect if 2dpiup were a random subset of the txome.
                    N.all,
                    sum(pfams.all.c$N)-N.all,
                    sum(pfamsdpi.cd$N), lower.tail = F),
           by="pfam.No"]

pfams.5dpi.cd <- pfams.all[dynamics=="1st down 5 dpi", .N, by="pfam.No"]
pfams.5dpi.cd <- merge(pfams.5dpi.cd, pfams.all.c[ , .(N.all, pfam.No)], by="pfam.No", all.x=T)
pfams.5dpi.cd[, p.overrep.down5 :=
             phyper(N,
                    N.all,
                    sum(pfams.all.c$N)-N.all,
                    sum(pfams.5dpi.cd$N), lower.tail = F),
           by="pfam.No"]

pfams.7dpi.cd <- pfams.all[dynamics=="1st down 7 dpi", .N, by="pfam.No"]
pfams.7dpi.cd <- merge(pfams.7dpi.cd, pfams.all.c[ , .(N.all, pfam.No)], by="pfam.No", all.x=T)
pfams.7dpi.cd[, p.overrep.down7 :=
             phyper(N,
                    N.all,
                    sum(pfams.all.c$N)-N.all,
                    sum(pfams.7dpi.cd$N), lower.tail = F),
           by="pfam.No"]

pfams.10dpi.cd <- pfams.all[dynamics=="1st down 10 dpi", .N, by="pfam.No"]
pfams.10dpi.cd <- merge(pfams.10dpi.cd, pfams.all.c[ , .(N.all, pfam.No)], by="pfam.No", all.x=T)
pfams.10dpi.cd[, p.overrep.down10 :=
             phyper(N,
                    N.all,
                    sum(pfams.all.c$N)-N.all,
                    sum(pfams.10dpi.cd$N), lower.tail = F),
           by="pfam.No"]

pfams.const <- pfams.all[dynamics=="constant", .N, by="pfam.No"]
pfams.const <- merge(pfams.const, pfams.all.c[ , .(N.all, pfam.No)], by="pfam.No", all.x=T)
pfams.const[, p.overrep.const :=
             phyper(N,
                    N.all,
                    sum(pfams.all.c$N)-N.all,
                    sum(pfams.const$N), lower.tail = F),
           by="pfam.No"]

#Collect the results of how often each pfam turns up in each dynamic category and whether that's significant enrichment
pfams.all.c <- merge(pfams.all.c, pfamsdpi.c[ , .(pfam.No, p.overrep.up2, N.up2=N)], by="pfam.No", all.x=T)
pfams.all.c <- merge(pfams.all.c, pfams.5dpi.c[ , .(pfam.No, p.overrep.up5, N.up5=N)], by="pfam.No", all.x=T)
pfams.all.c <- merge(pfams.all.c, pfams.7dpi.c[ , .(pfam.No, p.overrep.up7, N.up7=N)], by="pfam.No", all.x=T)
pfams.all.c <- merge(pfams.all.c, pfams.10dpi.c[ , .(pfam.No, p.overrep.up10, N.up10=N)], by="pfam.No", all.x=T)
pfams.all.c <- merge(pfams.all.c, pfamsdpi.cd[ , .(pfam.No, p.overrep.down2, N.down2=N)], by="pfam.No", all.x=T)
pfams.all.c <- merge(pfams.all.c, pfams.5dpi.cd[ , .(pfam.No, p.overrep.down5, N.down5=N)], by="pfam.No", all.x=T)
pfams.all.c <- merge(pfams.all.c, pfams.7dpi.cd[ , .(pfam.No, p.overrep.down7, N.down7=N)], by="pfam.No", all.x=T)
pfams.all.c <- merge(pfams.all.c, pfams.10dpi.cd[ , .(pfam.No, p.overrep.down10, N.down10=N)], by="pfam.No", all.x=T)
pfams.all.c <- merge(pfams.all.c, pfams.const[ , .(pfam.No, p.overrep.const, N.const=N)], by="pfam.No", all.x=T)

#Get into long format to be able to summarize properly
pfams.all.c.m1 <- melt(pfams.all.c, id.vars = "pfam.No",
                      measure.vars = c("N.up2", "N.up5", "N.up7", "N.up10",
                                       "N.down2", "N.down5", "N.down7", "N.down10", "N.const"),
                      variable.name = "dynamics", "N.in.category")
pfams.all.c.m2 <- melt(pfams.all.c, id.vars = "pfam.No",
                      measure.vars = c("p.overrep.up2", "p.overrep.up5", "p.overrep.up7", "p.overrep.up10", "p.overrep.down2", "p.overrep.down5", "p.overrep.down7", "p.overrep.down10",
                                       "p.overrep.const"),
                      variable.name = "dynamics", "p.overrep")
pfams.all.c.m <- merge(pfams.all.c.m1[ , .(pfam.No,
                                           dynamics=gsub("N.", "", as.character(dynamics)),
                                           N.in.category)],
                       pfams.all.c.m2[ , .(pfam.No,
                                           dynamics=gsub("p.overrep.", "", as.character(dynamics)),
                                           p.overrep)],
                       by=c("pfam.No", "dynamics"))

#Do BH correction of pvals, correcting for the number of tests done per pfam.No
pfams.all.c.m[ , p.overrep.adj := p.adjust(p.overrep, method = "BH"), by="pfam.No"]
pfams.all.c.m.signif <- pfams.all.c.m[p.overrep.adj<0.05, ]#Only look @significantly enriched pfams

#annotate the enriched pfam.Nos with DE
#read and re-format the explanations of pfam IDs, I'm using Pfam 30.0
pfam.explained <- read.delim("raw.data\\Pfam-A.hmm.dat", strip.white = T, stringsAsFactors = F, header = F)
pfam.explained.cols <- data.frame(ID=c(1:16306))
pfam.explained.cols[ ,"ID"] <- substr(pfam.explained[grep(" ID ", pfam.explained$V1), "V1"], 9, 25)
pfam.explained.cols[ ,"AC"] <- substr(pfam.explained[grep(" AC ", pfam.explained$V1), "V1"], 9, 17)
pfam.explained.cols[ ,"DE"] <- substr(pfam.explained[grep(" DE ", pfam.explained$V1), "V1"], 9, 90)
pfam.explained.cols[ ,"TP"] <- substr(pfam.explained[grep(" TP ", pfam.explained$V1), "V1"], 9, 21)
pfam.explained.cols <- data.table(pfam.explained.cols, key="AC")
setnames(pfam.explained.cols, "AC", "pfam.No")
setnames(pfam.explained.cols, "ID", "pfam.ID")
pfam.explained.cols$pfam.No <- trimws(pfam.explained.cols$pfam.No, which = "both")
pfam.explained <- data.table(pfam.explained.cols)
rm(pfam.explained.cols)

#annotate and write out all significantly enriched pfams to look at
pfams.all.c.m.signif.annot <- merge(pfams.all.c.m.signif,
                                    pfam.explained[, .(pfam.No, DE)], by="pfam.No", all.x = T)
write.csv(pfams.all.c.m.signif.annot, "analysis\\pfams.tr.curatedDB.csv", row.names = F)#This is Table S6
```

### Make the transcript-level dynamics plot underlying Figure 6c

```
library(data.table)
res.all.annot <- data.table(read.csv("analysis//AtRNA.1stat.curatedDB.res.all.annot.csv",
                                     stringsAsFactors = F))
triangledata <- res.all.annot[ , .N, by="dynamics"]
triangledata[dynamics %like% "2" , time := 2]
triangledata[dynamics %like% "5" , time := 5]
triangledata[dynamics %like% "7" , time := 7]
triangledata[dynamics %like% "10" , time := 10]
triangledata[dynamics %like% "const" , time := 13]
triangledata[dynamics %like% "up", direction := 1]
triangledata[dynamics %like% "down", direction := -1]
triangledata[dynamics %like% "const" , direction := 0]

library(ggplot2)
```

```
## Warning: package 'ggplot2' was built under R version 3.2.5
```

```
t <- ggplot(triangledata, aes(x=time, y=direction))
t +
  geom_point(data=triangledata[!is.na(direction),], aes(size=N, colour=as.character(direction)))+
  scale_size(range=c(5, 35),
    #limits = c(1000, 6000),
   # breaks = c(1000, 2000, 4000, 6000),
    "number of\ntranscripts",
                     guide=F) +
  scale_x_continuous(limits=c(1, 14.5), breaks=c(2, 5, 7, 10)) +
  scale_y_continuous(limits=c(-5, 5), breaks=c(-1, 1), labels=c("down", "up"))+
  theme_bw(base_size = 15) +
  scale_color_manual(values = c("#d35400", "#5499c7", "#52be80"),
                     labels=c("down", "constant"), "direction\nof change",
                     guide=F) +
  geom_text(data = triangledata[!is.na(direction)],
            aes(label=N), #size=3,
            check_overlap = T) +
  labs(x="days post agroinfiltration", y="transcriptional regulation")
```
