# Supplementary material for: The transcriptome, extracellular proteome and active secretome of agroinfiltrated Nicotiana benthamiana uncover a large, diverse protease repertoire
Source: Plant Biotechnol J. 2017 Dec 17;16(5):1068–84. doi: 10.1111/pbi.12852 (PMC5902771; doi:10.1111/pbi.12852)
Supplement: Supplementary file 22 — Appendix S3 R code used for extracellular proteome data analysis [file PBI-16-1068-s015.html]

Statistical analysis of apoplastic proteome data


# Statistical analysis of apoplastic proteome data

#### *FGH*

#### *24 January 2017*

Steps:

- In Perseus: Removal of proteins detected in the reverse or contaminant databases or only identified by site, log2 transformation and filtering for valid values (in at least 3 samples in either Agro (incl p19) or Buffer).
- In Perseus: impute missing values with default settings File named “ACE\_0056\_curatedDB\_flfi.txt”. Load this file for statistical analysis. Use the plant proteins only.
- Normalize LFQ by sample volume (For MS, we used 3 ug protein per MS sample to ensure detection of low abundant proteins).
- make euclidean distance heatmap for the proteome
- calculate day-by-day t-tests with BH correction for Agro vs Buffer
- group proteins by when their abundance first changes significantly and more than two-fold in response to agroinfiltration
- annotate the proteins and test for enrichment of pfam annotations in each regulatory group vs the whole apoplastic proteome
- Plot the sizes of the regulatory groups for Figure 6d

### Importing and normalization of MS data

```
library(data.table)
# load the file from perseus after removal of usual suspects, log2
# transformation and filtering for valid values (in at least 3 samples in
# either Agro (incl p19) or Buffer).
MS <- data.table(read.delim("raw.data\\ACE_0056_curatedDB_flfi.txt", stringsAsFactors = F, 
    comment.char = "#"))
nameconv <- data.table(read.csv("analysis\\At.MS.nameconversion.done.csv", stringsAsFactors = F))
setnames(MS, nameconv$MS.ID, nameconv$My.ID)
rm(nameconv)
Buf <- c("A2B", "B2B", "C2B", "A5B", "B5B", "C5B", "A7B", "B7B", "C7B", "A10B", 
    "B10B", "C10B")
Agro <- c("A2W", "B2W", "C2W", "A5W", "B5W", "C5W", "A7W", "B7W", "C7W", "A10W", 
    "B10W", "C10W", "A2P", "B2P", "C2P", "A5P", "B5P", "C5P", "A7P", "B7P", 
    "C7P", "A10P", "B10P", "C10P")
samples <- c("A2B", "B2B", "C2B", "A5B", "B5B", "C5B", "A7B", "B7B", "C7B", 
    "A10B", "B10B", "C10B", "A2W", "B2W", "C2W", "A5W", "B5W", "C5W", "A7W", 
    "B7W", "C7W", "A10W", "B10W", "C10W", "A2P", "B2P", "C2P", "A5P", "B5P", 
    "C5P", "A7P", "B7P", "C7P", "A10P", "B10P", "C10P")
# only Niben
MS <- MS[(Protein.IDs %like% "Nbv") | (Protein.IDs %like% "Niben") | (Protein.IDs %like% 
    "Nicotiana") | (Protein.IDs %like% "p19"), ]

# normalize by volume
MS.l <- melt(MS, id.vars = c("Protein.IDs", "Majority.protein.IDs"), measure.vars = samples, 
    variable.name = "sample", value.name = "LFQ")
cc <- data.table(read.csv("raw.data\\At.samples.protein.cc.csv", stringsAsFactors = F))
MS.l <- merge(MS.l, cc[, .(sample, log2.v.mean..v.sample..)], by = "sample", 
    all = T)
MS.l[, `:=`(norm.LFQ, LFQ + log2.v.mean..v.sample..)]
MS.l$log2.v.mean..v.sample.. <- NULL

# Make a file to be used for Euclidean distance clustering
MS.normbyvol <- dcast(MS.l, Protein.IDs + Majority.protein.IDs ~ sample, value.var = "norm.LFQ")
write.csv(MS.normbyvol, "analysis\\MS.curatedDB.normbyvol.csv", row.names = F)
```

### Euclidean distance clustering as shown for transcriptome in Figure 6b

```
suppressPackageStartupMessages(library(dendextend))
```

```
## Warning: package 'dendextend' was built under R version 3.2.5
```

```
## Warning: replacing previous import by 'magrittr::%>%' when loading
## 'dendextend'
```

```
# making distance heatmap
At.MS.raw <- data.table(read.csv("analysis\\MS.curatedDB.normbyvol.csv", stringsAsFactors = F))
library(RColorBrewer)
library(gplots)
```

```
## Warning: package 'gplots' was built under R version 3.2.5
```

```
## 
## Attaching package: 'gplots'
```

```
## The following object is masked from 'package:stats':
## 
##     lowess
```

```
At.MS.raw.m <- dist(t(data.frame(At.MS.raw[, c("A2B", "B2B", "C2B", "A5B", "B5B", 
    "C5B", "A7B", "B7B", "C7B", "A10B", "B10B", "C10B", "A2W", "B2W", "C2W", 
    "A2P", "B2P", "C2P", "A5W", "B5W", "C5W", "A5P", "B5P", "C5P", "A7W", "B7W", 
    "C7W", "A7P", "B7P", "C7P", "A10W", "B10W", "C10W", "A10P", "B10P", "C10P"), 
    with = F])))
At.MS.raw.hc <- hclust(At.MS.raw.m)
# cosmetics for plotting
library(dendextend)
At.MS.raw.dd <- rotate(as.dendrogram(At.MS.raw.hc), c("A2B", "B2B", "C2B", "A5B", 
    "B5B", "C5B", "A7B", "B7B", "C7B", "A10B", "B10B", "C10B", "A2W", "B2W", 
    "C2W", "A2P", "B2P", "C2P", "A5W", "B5W", "C5W", "A5P", "B5P", "C5P", "A7W", 
    "B7W", "C7W", "A7P", "B7P", "C7P", "A10W", "B10W", "C10W", "A10P", "B10P", 
    "C10P"))

At.MS.raw.m <- as.matrix(At.MS.raw.m)

library(RColorBrewer)
newcol <- colorRampPalette(brewer.pal(9, "Blues"))
ncols <- 50
bluecols2 <- newcol(ncols)
heatmap.2(At.MS.raw.m, Rowv = At.MS.raw.dd, Colv = At.MS.raw.dd, trace = "none", 
    main = "Euclidean sample distances\nfor proteomes of agroinfiltrated leaves", 
    density.info = "none", col = rev(bluecols2))
```

### Statistical analysis & dynamics categorization

Categorize by when a protein is first regulated significantly and more than 2-fold (2, 5, 7 or 10 dpi) and whether that regulation is up or down.

```
# calculate day-by-day t-tests with BH correction for Agro vs Buffer make
# the factors I need
MS.l[sample %like% "W" | sample %like% "P", `:=`(treatment, "Agro")]
MS.l[sample %like% "[0-9]B", `:=`(treatment, "Buffer")]
MS.l[, `:=`(dpi, gsub("[A-Z]{1}([0-9]{1,2})[A-Z]", "\\1", sample))]
MS.l[, `:=`(bio.repl, substr(sample, 1, 1))]
# MS.l[ , .N/3, by=c('treatment', 'dpi')] so everything still in here

# get the data I need in long format
MS.l[, `:=`(av.biorepls, mean(norm.LFQ)), by = c("Majority.protein.IDs", "treatment", 
    "dpi")]
MS.lfcs <- merge(MS.l[treatment %in% c("Agro"), ], unique(MS.l[treatment == 
    "Buffer", .(Protein.IDs, av.Buf = av.biorepls, bio.repl, dpi)]), by = c("Protein.IDs", 
    "bio.repl", "dpi"), all.x = T)
MS.lfcs[, `:=`(lfc.AgrovsBuf, norm.LFQ - av.Buf), by = c("Protein.IDs", "sample")]
MS.lfcs.p <- merge(MS.lfcs, MS.l[treatment == "Buffer", .(Protein.IDs, norm.LFQ.Buf = norm.LFQ, 
    bio.repl, dpi)], by = c("Protein.IDs", "bio.repl", "dpi"), all.x = T)

# do the t-tests & BH correction
MS.lfcs.p[, `:=`(p.AgrovsBuf, t.test(norm.LFQ, norm.LFQ.Buf, alternative = "two.sided")$p.value), 
    by = c("Protein.IDs", "treatment", "dpi")]
MS.lfcs.p[, `:=`(padj.AgrovsBuf, p.adjust(p.AgrovsBuf, method = "BH")), by = c("treatment", 
    "dpi")]

# name regulatory categories

# get averaged lfcs (av of bio.repls) and adjusted pvals in wide format
MS.lfcs.p[, `:=`(av.lfc.AgrovsBuf, mean(lfc.AgrovsBuf)), by = c("Protein.IDs", 
    "treatment", "dpi")]
MS.dyn <- dcast(unique(MS.lfcs.p[, .(Protein.IDs, av.lfc.AgrovsBuf, padj.AgrovsBuf, 
    treatment, dpi)]), Protein.IDs ~ treatment + dpi, value.var = c("av.lfc.AgrovsBuf", 
    "padj.AgrovsBuf"))

setnames(MS.dyn, c("av.lfc.AgrovsBuf_Agro_10", "av.lfc.AgrovsBuf_Agro_2", "av.lfc.AgrovsBuf_Agro_5", 
    "av.lfc.AgrovsBuf_Agro_7", "padj.AgrovsBuf_Agro_10", "padj.AgrovsBuf_Agro_2", 
    "padj.AgrovsBuf_Agro_5", "padj.AgrovsBuf_Agro_7"), c("lfc_10", "lfc_2", 
    "lfc_5", "lfc_7", "padj_10", "padj_2", "padj_5", "padj_7"))

MS.dyn[lfc_2 > 1 & padj_2 < 0.05, `:=`(dynamics, "1st up 2 dpi")]
MS.dyn[!(abs(lfc_2) > 1 & padj_2 < 0.05) & lfc_5 > 1 & padj_5 < 0.05, `:=`(dynamics, 
    "1st up 5 dpi")]
MS.dyn[!((abs(lfc_2) > 1 & padj_2 < 0.05) | (abs(lfc_5) > 1 & padj_5 < 0.05)) & 
    lfc_7 > 1 & padj_7 < 0.05, `:=`(dynamics, "1st up 7 dpi")]
MS.dyn[!((abs(lfc_2) > 1 & padj_2 < 0.05) | (abs(lfc_5) > 1 & padj_5 < 0.05) | 
    (abs(lfc_7) > 1 & padj_7 < 0.05)) & lfc_10 > 1 & padj_10 < 0.05, `:=`(dynamics, 
    "1st up 10 dpi")]

MS.dyn[lfc_2 < (-1) & padj_2 < 0.05, `:=`(dynamics, "1st down 2 dpi")]
MS.dyn[!(abs(lfc_2) > 1 & padj_2 < 0.05) & lfc_5 < (-1) & padj_5 < 0.05, `:=`(dynamics, 
    "1st down 5 dpi")]
MS.dyn[!((abs(lfc_2) > 1 & padj_2 < 0.05) | (abs(lfc_5) > 1 & padj_5 < 0.05)) & 
    lfc_7 < (-1) & padj_7 < 0.05, `:=`(dynamics, "1st down 7 dpi")]
MS.dyn[!((abs(lfc_2) > 1 & padj_2 < 0.05) | (abs(lfc_5) > 1 & padj_5 < 0.05) | 
    (abs(lfc_7) > 1 & padj_7 < 0.05)) & lfc_10 < (-1) & padj_10 < 0.05, `:=`(dynamics, 
    "1st down 10 dpi")]

MS.dyn[!((abs(lfc_2) > 1 & padj_2 < 0.05) | (abs(lfc_5) > 1 & padj_5 < 0.05) | 
    (abs(lfc_7) > 1 & padj_7 < 0.05) | (abs(lfc_10) > 1 & padj_10 < 0.05)), 
    `:=`(dynamics, "constant")]

MS.dyn$dynamics <- factor(MS.dyn$dynamics, levels = c("1st up 2 dpi", "1st up 5 dpi", 
    "1st up 7 dpi", "1st up 10 dpi", "1st down 2 dpi", "1st down 5 dpi", "1st down 7 dpi", 
    "1st down 10 dpi", "constant"))
dyncounts <- MS.dyn[, .N, by = "dynamics"]
```

### Annotation and pfam family enrichment analysis

```
#annotate the protein data
###########################
annot <- data.table(read.csv("raw.data\\annot.curated.csv", stringsAsFactors = F))#This file was obtained by passing the predicted proteins through PFAM 30 and signalP 4.0

IDs.MS <- unique(MS.dyn[ , .(Protein.IDs)])
IDs.MS.l <- IDs.MS[ , .(ID = unlist(strsplit(Protein.IDs, ";"))), by="Protein.IDs"]
IDs.MS.l <- merge(IDs.MS.l, annot, all.x = T, by="ID")
IDs.MS.annot <- IDs.MS.l[ , lapply(.SD, paste, collapse=";"), by="Protein.IDs"]

IDs.MS.annot.s <- IDs.MS.annot[ , lapply(.SD, function(x) gsub(";NA", "", x)), by="Protein.IDs"]
IDs.MS.annot.s <- IDs.MS.annot.s[ , lapply(.SD, function(x) gsub("NA;", "", x)), by="Protein.IDs"]

dedup <- function(col.name){
  vapply(lapply(strsplit(col.name, ";"), unique), paste, character(1L), collapse = ";")
}

IDs.MS.annot.s[ , MEROPS.family := dedup(MEROPS.family)]
IDs.MS.annot.s[ , ppase.cattype := dedup(ppase.cattype)]
IDs.MS.annot.s[ , MEROPS.subfamily := dedup(MEROPS.subfamily)]
IDs.MS.annot.s[ , CAZY.family := dedup(CAZY.family)]
IDs.MS.annot.s[ , signalP := dedup(signalP)]
IDs.MS.annot.s[ , pfam.No := dedup(pfam.No)]
IDs.MS.annot.s[ , DE := dedup(DE)]
IDs.MS.annot.s <- IDs.MS.annot.s[ , lapply(.SD, function(x) gsub("^NA$", NA, x)), by="ID"]

IDs.MS.annot.s[MEROPS.family %like% "I09;S08", MEROPS.family := "S08"]
IDs.MS.annot.s[ppase.cattype %like% "I;S", ppase.cattype := "S"]

IDs.MS <- IDs.MS.annot.s
rm(IDs.MS.annot.s, IDs.MS.annot, IDs.MS.l, annot)
MS.dyn.annot <- merge(MS.dyn, IDs.MS, by="Protein.IDs")
write.csv(MS.dyn.annot, "analysis\\MS.curatedDB.dyn.annot.csv", row.names = F)

library(data.table)
MS.dyn.annot <- data.table(read.csv("analysis\\MS.curatedDB.dyn.annot.csv", stringsAsFactors = F))

#count pfam representation in each dynamic category
###################################################
pfams.all <- MS.dyn.annot[, .(pfam.No=unlist(strsplit(pfam.No, ";")), dynamics), by="Protein.IDs"]
pfams.all.c <- pfams.all[ , .(N.all = .N), by="pfam.No"]#get all pfams in proteome and how often they are represented

#Count how often each pfam turns up in each dynamic category and whether that's significant enrichment
pfams.2dpi.c <- pfams.all[dynamics=="1st up 2 dpi", .N, by="pfam.No"]#count 2dpi
pfams.2dpi.c <- merge(pfams.2dpi.c, pfams.all.c[ , .(N.all, pfam.No)], by="pfam.No", all.x=T)#get counts in all
pfams.2dpi.c[, p.overrep.up2 :=
             phyper(N,#see help->phyper. We are testing whether finding this pfam N or more times is significantly different than what you would expect if 2dpiup were a random subset of the txome.
                    N.all,
                    sum(pfams.all.c$N)-N.all,
                    sum(pfams.2dpi.c$N), lower.tail = F),
           by="pfam.No"]

pfams.5dpi.c <- pfams.all[dynamics=="1st up 5 dpi", .N, by="pfam.No"]
pfams.5dpi.c <- merge(pfams.5dpi.c, pfams.all.c[ , .(N.all, pfam.No)], by="pfam.No", all.x=T)
pfams.5dpi.c[, p.overrep.up5 :=
             phyper(N,
                    N.all,
                    sum(pfams.all.c$N)-N.all,
                    sum(pfams.5dpi.c$N), lower.tail = F),
           by="pfam.No"]

pfams.7dpi.c <- pfams.all[dynamics=="1st up 7 dpi", .N, by="pfam.No"]
pfams.7dpi.c <- merge(pfams.7dpi.c, pfams.all.c[ , .(N.all, pfam.No)], by="pfam.No", all.x=T)
pfams.7dpi.c[, p.overrep.up7 :=
             phyper(N,
                    N.all,
                    sum(pfams.all.c$N)-N.all,
                    sum(pfams.7dpi.c$N), lower.tail = F),
           by="pfam.No"]

pfams.10dpi.c <- pfams.all[dynamics=="1st up 10 dpi", .N, by="pfam.No"]
pfams.10dpi.c <- merge(pfams.10dpi.c, pfams.all.c[ , .(N.all, pfam.No)], by="pfam.No", all.x=T)
pfams.10dpi.c[, p.overrep.up10 :=
             phyper(N,
                    N.all,
                    sum(pfams.all.c$N)-N.all,
                    sum(pfams.10dpi.c$N), lower.tail = F),
           by="pfam.No"]

#count down categories
pfams.2dpi.cd <- pfams.all[dynamics=="1st down 2 dpi", .N, by="pfam.No"]#count 2dpi
pfams.2dpi.cd <- merge(pfams.2dpi.cd, pfams.all.c[ , .(N.all, pfam.No)], by="pfam.No", all.x=T)#get counts in all
pfams.2dpi.cd[, p.overrep.down2 :=
             phyper(N,#see help->phyper. We are testing whether finding this pfam N or more times is significantly different than what you would expect if 2dpiup were a random subset of the txome.
                    N.all,
                    sum(pfams.all.c$N)-N.all,
                    sum(pfams.2dpi.cd$N), lower.tail = F),
           by="pfam.No"]

pfams.5dpi.cd <- pfams.all[dynamics=="1st down 5 dpi", .N, by="pfam.No"]
pfams.5dpi.cd <- merge(pfams.5dpi.cd, pfams.all.c[ , .(N.all, pfam.No)], by="pfam.No", all.x=T)
pfams.5dpi.cd[, p.overrep.down5 :=
             phyper(N,
                    N.all,
                    sum(pfams.all.c$N)-N.all,
                    sum(pfams.5dpi.cd$N), lower.tail = F),
           by="pfam.No"]

pfams.7dpi.cd <- pfams.all[dynamics=="1st down 7 dpi", .N, by="pfam.No"]
pfams.7dpi.cd <- merge(pfams.7dpi.cd, pfams.all.c[ , .(N.all, pfam.No)], by="pfam.No", all.x=T)
pfams.7dpi.cd[, p.overrep.down7 :=
             phyper(N,
                    N.all,
                    sum(pfams.all.c$N)-N.all,
                    sum(pfams.7dpi.cd$N), lower.tail = F),
           by="pfam.No"]

pfams.10dpi.cd <- pfams.all[dynamics=="1st down 10 dpi", .N, by="pfam.No"]
pfams.10dpi.cd <- merge(pfams.10dpi.cd, pfams.all.c[ , .(N.all, pfam.No)], by="pfam.No", all.x=T)
pfams.10dpi.cd[, p.overrep.down10 :=
             phyper(N,
                    N.all,
                    sum(pfams.all.c$N)-N.all,
                    sum(pfams.10dpi.cd$N), lower.tail = F),
           by="pfam.No"]

pfams.const <- pfams.all[dynamics=="constant", .N, by="pfam.No"]
pfams.const <- merge(pfams.const, pfams.all.c[ , .(N.all, pfam.No)], by="pfam.No", all.x=T)
pfams.const[, p.overrep.const :=
             phyper(N,
                    N.all,
                    sum(pfams.all.c$N)-N.all,
                    sum(pfams.const$N), lower.tail = F),
           by="pfam.No"]

#Collect the results of how often each pfam turns up in each dynamic category and whether that's significant enrichment
pfams.all.c <- merge(pfams.all.c, pfams.2dpi.c[ , .(pfam.No, p.overrep.up2, N.up2=N)], by="pfam.No", all.x=T)
pfams.all.c <- merge(pfams.all.c, pfams.5dpi.c[ , .(pfam.No, p.overrep.up5, N.up5=N)], by="pfam.No", all.x=T)
pfams.all.c <- merge(pfams.all.c, pfams.7dpi.c[ , .(pfam.No, p.overrep.up7, N.up7=N)], by="pfam.No", all.x=T)
pfams.all.c <- merge(pfams.all.c, pfams.10dpi.c[ , .(pfam.No, p.overrep.up10, N.up10=N)], by="pfam.No", all.x=T)
pfams.all.c <- merge(pfams.all.c, pfams.2dpi.cd[ , .(pfam.No, p.overrep.down2, N.down2=N)], by="pfam.No", all.x=T)
pfams.all.c <- merge(pfams.all.c, pfams.5dpi.cd[ , .(pfam.No, p.overrep.down5, N.down5=N)], by="pfam.No", all.x=T)
pfams.all.c <- merge(pfams.all.c, pfams.7dpi.cd[ , .(pfam.No, p.overrep.down7, N.down7=N)], by="pfam.No", all.x=T)
pfams.all.c <- merge(pfams.all.c, pfams.10dpi.cd[ , .(pfam.No, p.overrep.down10, N.down10=N)], by="pfam.No", all.x=T)
pfams.all.c <- merge(pfams.all.c, pfams.const[ , .(pfam.No, p.overrep.const, N.const=N)], by="pfam.No", all.x=T)

#Get into long format to be able to summarize properly
pfams.all.c.m1 <- melt(pfams.all.c, id.vars = "pfam.No",
                      measure.vars = c("N.up2", "N.up5", "N.up7", "N.up10",
                                       "N.down2", "N.down5", "N.down7", "N.down10", "N.const"),
                      variable.name = "dynamics", "N.in.category")
pfams.all.c.m2 <- melt(pfams.all.c, id.vars = "pfam.No",
                      measure.vars = c("p.overrep.up2", "p.overrep.up5", "p.overrep.up7", "p.overrep.up10", "p.overrep.down2", "p.overrep.down5", "p.overrep.down7", "p.overrep.down10",
                                       "p.overrep.const"),
                      variable.name = "dynamics", "p.overrep")
pfams.all.c.m <- merge(pfams.all.c.m1[ , .(pfam.No,
                                           dynamics=gsub("N.", "", as.character(dynamics)),
                                           N.in.category)],
                       pfams.all.c.m2[ , .(pfam.No,
                                           dynamics=gsub("p.overrep.", "", as.character(dynamics)),
                                           p.overrep)],
                       by=c("pfam.No", "dynamics"))

#Do BH correction of pvals, correcting for the number of tests done per pfam.No
pfams.all.c.m[ , p.overrep.adj := p.adjust(p.overrep, method = "BH"), by="pfam.No"]
pfams.all.c.m.signif <- pfams.all.c.m[p.overrep.adj<0.05, ]#Only look @significantly enriched pfams

#annotate the enriched pfam.Nos with DE
#read and re-format the explanations of pfam IDs, I'm using Pfam 30.0
pfam.explained <- read.delim("raw.data\\Pfam-A.hmm.dat", strip.white = T, stringsAsFactors = F, header = F)
pfam.explained.cols <- data.frame(ID=c(1:16306))
pfam.explained.cols[ ,"ID"] <- substr(pfam.explained[grep(" ID ", pfam.explained$V1), "V1"], 9, 25)
pfam.explained.cols[ ,"AC"] <- substr(pfam.explained[grep(" AC ", pfam.explained$V1), "V1"], 9, 17)
pfam.explained.cols[ ,"DE"] <- substr(pfam.explained[grep(" DE ", pfam.explained$V1), "V1"], 9, 90)
pfam.explained.cols[ ,"TP"] <- substr(pfam.explained[grep(" TP ", pfam.explained$V1), "V1"], 9, 21)
pfam.explained.cols <- data.table(pfam.explained.cols, key="AC")
setnames(pfam.explained.cols, "AC", "pfam.No")
setnames(pfam.explained.cols, "ID", "pfam.ID")
pfam.explained.cols$pfam.No <- trimws(pfam.explained.cols$pfam.No, which = "both")
pfam.explained <- data.table(pfam.explained.cols)
rm(pfam.explained.cols)
rm(IDs.MS, MS, MS.dyn, MS.l, MS.lfcs,
   pfams.all.c.m2, pfams.all.c.m1, pfams.all.c)

all.enriched.pfams <- merge(pfams.all.c.m.signif, pfam.explained[ , .(pfam.No, DE)],
                            by="pfam.No", all.x = T)
all.enriched.pfams$dynamics <- factor(all.enriched.pfams$dynamics, levels=c(
  "up2", "up5", "up7", "up10",
  "down2", "down5", "down7", "down10", "const"))
write.csv(all.enriched.pfams[order(dynamics)],
          "analysis\\all.enriched.pfams.prot.csv", row.names = F)#This is Table S7
```

### Make the protein-level dynamics plot underlying Figure 6d

```
library(ggplot2)
```

```
## Warning: package 'ggplot2' was built under R version 3.2.5
```

```
library(data.table)
MS.dyn.annot <- data.table(read.csv("analysis\\MS.curatedDB.dyn.annot.csv", stringsAsFactors = F))

triangledata <- MS.dyn.annot[ , .N, by="dynamics"]
triangledata[dynamics %like% "2" , time := 2]
triangledata[dynamics %like% "5" , time := 5]
triangledata[dynamics %like% "7" , time := 7]
triangledata[dynamics %like% "10" , time := 10]
triangledata[dynamics %like% "const" , time := 13]
triangledata[dynamics %like% "up", direction := 1]
triangledata[dynamics %like% "down", direction := -1]
triangledata[dynamics %like% "const" , direction := 0]


#try coloured circles instead of triangles
t <- ggplot(triangledata, aes(x=time, y=direction))
t +
  geom_point(data=triangledata[!is.na(direction),],
             aes(size=N, colour=as.character(direction)))+
  scale_size(range=c(5, 35),
    "number of\nproteins",
                     guide=F) +
  scale_x_continuous(limits=c(1, 14.5), breaks=c(2, 5, 7, 10)) +
  scale_y_continuous(limits=c(-5, 5), breaks=c(-1, 1), labels=c("down", "up"))+
  theme_bw() +
  scale_color_manual(values = c("#d35400", "#5499c7", "#52be80"),
                     labels=c("down", "constant"), "direction\nof change",
                     guide=F) +
  geom_text(data = triangledata[!is.na(direction)],
            aes(label=N), #size=3,
            check_overlap = T) +
  labs(x="days post agroinfiltration", y="apoplast protein level")
```
