# Supplementary material for: The transcriptome, extracellular proteome and active secretome of agroinfiltrated Nicotiana benthamiana uncover a large, diverse protease repertoire
Source: Plant Biotechnol J. 2017 Dec 17;16(5):1068–84. doi: 10.1111/pbi.12852 (PMC5902771; doi:10.1111/pbi.12852)
Supplement: Supplementary file 23 — Appendix S4 R code used for analysis of the effects of leaf ageing [file PBI-16-1068-s019.html]

Transcript and protein dynamics over time


# Transcript and protein dynamics over time

#### *FGH*

#### *10 April 2017*

# Transcriptome changes over time

```
library(DESeq2)
```

```
## Warning: package 'Rcpp' was built under R version 3.2.5
```

```
## Warning: package 'RcppArmadillo' was built under R version 3.2.5
```

```
library(data.table)
#reading in & cosmetics
count.data.r <- read.csv("analysis\\count.data.salmonv0.7.csv", row.names = 1, strip.white = T)#This file contains the NumReads output of salmon
count.data.r$sumcounts <- NULL
count.data.r$ID <- row.names(count.data.r)
count.data.r <- data.table(count.data.r)
count.data.r <- count.data.r[ID %like% "Niben" |ID %like% "Nbv" |ID %like% "Nicotiana" |
                   ID %like% "p19", ]# only looking @ the plant
count.data.r <- data.frame(count.data.r[, c(1:36), with=F], row.names = count.data.r[ , ID])
count.data.r$ID <- NULL

col.data.noB5W.AgrovsBuf <- read.csv("raw.data\\col.data.noB5W.WTvsP19vsBuf.csv", row.names = 1, strip.white = T)
names(col.data.noB5W.AgrovsBuf) <- c("treatment", "dpi")
col.data.noB5W.AgrovsBuf$treatment <- factor(col.data.noB5W.AgrovsBuf$treatment, levels = c("Buffer", "Agro.WT", "Agro.p19"))
col.data.noB5W.AgrovsBuf$dpi <- factor(col.data.noB5W.AgrovsBuf$dpi, levels = c("2", "5", "7", "10"))

dds.At.RNA <- DESeqDataSetFromMatrix(countData = count.data.r, colData = col.data.noB5W.AgrovsBuf,
                                     design = ~ dpi)

#size factors (to account for differences in sequencing depth) are only based on transcripts with >3500 NumReads.
#By visual inspection of the density of NumRead values in different samples before and after normalization,
#this was determined to be the optimal treshold. 
count.data.forsizeFactors <- count.data.r
count.data.forsizeFactors <- data.table(count.data.forsizeFactors)
count.data.forsizeFactors <- count.data.forsizeFactors[, sumcounts := Reduce(`+`, .SD)]
count.data.forsizeFactors.100 <- count.data.forsizeFactors[which(sumcounts>3500),]
count.data.forsizeFactors <- data.frame(count.data.forsizeFactors.100)
count.data.forsizeFactors$sumcounts <- NULL
dds.forsizefactors <- DESeqDataSetFromMatrix(count.data.forsizeFactors, col.data.noB5W.AgrovsBuf, design = ~ dpi)
dds.forsizefactors <- estimateSizeFactors(dds.forsizefactors)

sizeFactors(dds.At.RNA) <- sizeFactors(dds.forsizefactors)
dds.At.RNA <- estimateDispersions(dds.At.RNA)
dds.At.RNA <- nbinomLRT(dds.At.RNA, reduced = ~ 1, betaPrior = T)

#get the lfcs & padj (BH, as per default) for each timepoint
results.At.RNA <- results(dds.At.RNA, alpha = 0.05,
                          cooksCutoff = F,#turn outlier filtering off 
                          contrast = c("dpi", "5", "2"))
res5dpi <- data.frame(results.At.RNA)
res5dpi[ , "ID"] <- row.names(res5dpi)
res5dpi <- data.table(res5dpi)
setnames(res5dpi, c("log2FoldChange", "padj"), c("lfc5", "padj5"))

results.At.RNA <- results(dds.At.RNA, alpha = 0.05,
                          cooksCutoff = F,#turn outlier filtering off 
                          contrast = c("dpi", "7", "2"))
res7dpi <- data.frame(results.At.RNA)
res7dpi[ , "ID"] <- row.names(res7dpi)
res7dpi <- data.table(res7dpi)
setnames(res7dpi, c("log2FoldChange", "padj"), c("lfc7", "padj7"))

results.At.RNA <- results(dds.At.RNA, alpha = 0.05,
                          cooksCutoff = F,#turn outlier filtering off 
                          contrast = c("dpi", "10", "2"))
res10dpi <- data.frame(results.At.RNA)
res10dpi[ , "ID"] <- row.names(res10dpi)
res10dpi <- data.table(res10dpi)
setnames(res10dpi, c("log2FoldChange", "padj"), c("lfc10", "padj10"))

lfcs.time <- merge(res5dpi[ , .(ID, lfc5, padj5)],
                   res7dpi[ , .(ID, lfc7, padj7)],
                   by="ID")
lfcs.time <- merge(lfcs.time,
                   res10dpi[ , .(ID, lfc10, padj10)],
                   by="ID")

#name dynamics groups: up/down only if all are lfc>1 and padj<0.05
lfcs.time[lfc5>0 & padj5<0.05 & lfc7>0 & padj7<0.05 & lfc10>0 & padj10<0.05, dynamics:="up.over.time"]
lfcs.time[lfc5<0 & padj5<0.05 & lfc7<0 & padj7<0.05 & lfc10<0 & padj10<0.05,
          dynamics:="down.over.time"]

lfcs.time[ , .N, by="dynamics"]
```

### Annotation

```
annot <- data.table(read.csv("raw.data\\annot.curated.csv", stringsAsFactors = F))

lfcs.time.annot <- merge(lfcs.time, annot, by = "ID", all.x = T)
write.csv(lfcs.time.annot, "analysis//lfcs.time.annot.csv", row.names = F)
```

### Pfam family enrichment analysis

```
library(data.table)
lfcs.time.annot <- data.table(read.csv("analysis//lfcs.time.annot.csv", stringsAsFactors = F))

pfams.all <- lfcs.time.annot[, .(pfam.No=unlist(strsplit(pfam.No, ";")), dynamics), by="ID"]
pfams.all.c <- pfams.all[ , .(N.all = .N), by="pfam.No"]#get all pfams in txome and how often they are represented

#Count how often each pfam turns up in the up or downregulated things over time and whether that's significant enrichment
pfamsAgeUp.c <- pfams.all[dynamics=="up.over.time", .N, by="pfam.No"]
pfamsAgeUp.c <- merge(pfamsAgeUp.c, pfams.all.c[ , .(N.all, pfam.No)], by="pfam.No", all.x=T)
pfamsAgeUp.c[, p.overrep.AgeUp :=
             phyper(N,#see help->phyper. We are testing whether finding this pfam N or more times is significantly different than what you would expect if 2dpiup were a random subset of the txome.
                    N.all,
                    sum(pfams.all.c$N)-N.all,
                    sum(pfamsAgeUp.c$N), lower.tail = F),
           by="pfam.No"]

pfamsAgeDown.c <- pfams.all[dynamics=="down.over.time", .N, by="pfam.No"]
pfamsAgeDown.c <- merge(pfamsAgeDown.c, pfams.all.c[ , .(N.all, pfam.No)], by="pfam.No", all.x=T)
pfamsAgeDown.c[, p.overrep.AgeDown :=
             phyper(N,#see help->phyper. We are testing whether finding this pfam N or more times is significantly different than what you would expect if 2dpiDown were a random subset of the txome.
                    N.all,
                    sum(pfams.all.c$N)-N.all,
                    sum(pfamsAgeDown.c$N), lower.tail = F),
           by="pfam.No"]

#Collect the results of how often each pfam turns up in each dynamic category and whether that's significant enrichment
pfams.all.c <- merge(pfams.all.c, pfamsAgeUp.c[ , .(pfam.No, p.overrep.AgeUp, N.AgeUp=N)], by="pfam.No", all.x=T)
pfams.all.c <- merge(pfams.all.c, pfamsAgeDown.c[ , .(pfam.No, p.overrep.AgeDown, N.AgeDown=N)],
                     by="pfam.No", all.x=T)

#Get into long format to be able to summarize properly
pfams.all.c.m1 <- melt(pfams.all.c, id.vars = "pfam.No",
                      measure.vars = c("N.AgeUp", "N.AgeDown"),
                      variable.name = "dynamics", "N.in.category")
pfams.all.c.m2 <- melt(pfams.all.c, id.vars = "pfam.No",
                      measure.vars = c("p.overrep.AgeUp", "p.overrep.AgeDown"),
                      variable.name = "dynamics", "p.overrep")
pfams.all.c.m <- merge(pfams.all.c.m1[ , .(pfam.No,
                                           dynamics=gsub("N.", "", as.character(dynamics)),
                                           N.in.category)],
                       pfams.all.c.m2[ , .(pfam.No,
                                           dynamics=gsub("p.overrep.", "", as.character(dynamics)),
                                           p.overrep)],
                       by=c("pfam.No", "dynamics"))

#Do BH correction of pvals, correcting for the number of tests done per pfam.No
pfams.all.c.m[ , p.overrep.adj := p.adjust(p.overrep, method = "BH"), by="pfam.No"]
pfams.all.c.m.signif <- pfams.all.c.m[p.overrep.adj<0.05, ]#Only look @significantly enriched pfams

#annotate the enriched pfam.Nos with DE
#read and re-format the explanations of pfam IDs, I'm using Pfam 30.0
pfam.explained <- read.delim("raw.data\\Pfam-A.hmm.dat", strip.white = T, stringsAsFactors = F, header = F)
pfam.explained.cols <- data.frame(ID=c(1:16306))
pfam.explained.cols[ ,"ID"] <- substr(pfam.explained[grep(" ID ", pfam.explained$V1), "V1"], 9, 25)
pfam.explained.cols[ ,"AC"] <- substr(pfam.explained[grep(" AC ", pfam.explained$V1), "V1"], 9, 17)
pfam.explained.cols[ ,"DE"] <- substr(pfam.explained[grep(" DE ", pfam.explained$V1), "V1"], 9, 90)
pfam.explained.cols[ ,"TP"] <- substr(pfam.explained[grep(" TP ", pfam.explained$V1), "V1"], 9, 21)
pfam.explained.cols <- data.table(pfam.explained.cols, key="AC")
setnames(pfam.explained.cols, "AC", "pfam.No")
setnames(pfam.explained.cols, "ID", "pfam.ID")
pfam.explained.cols$pfam.No <- trimws(pfam.explained.cols$pfam.No, which = "both")
pfam.explained <- data.table(pfam.explained.cols)
rm(pfam.explained.cols)

#annotate and write out all significantly enriched pfams to look at
pfams.all.c.m.signif.annot <- merge(pfams.all.c.m.signif,
                                    pfam.explained[, .(pfam.No, DE)], by="pfam.No", all.x = T)
write.csv(pfams.all.c.m.signif.annot, "analysis\\pfams.tr.overtime.csv", row.names = F)#This is Table S14
```

So the transcripts upregulated in ageing plants contain an overrepresentation of PLCPs, P450s and NAM proteins (regulators of cell division). The ones downregulated in ageing plants are enriched for the usual suspects (chlorophyll binding and histones), but also for LRRs and Peroxidases. Find out which PLCPs these are!

```
library(knitr)
```

```
## Warning: package 'knitr' was built under R version 3.2.5
```

```
PLCPs.ageing <- lfcs.time.annot[MEROPS.family %like% "C01" & !(is.na(dynamics)), 
    .(ID, dynamics)]
C01.domains <- data.table(read.csv("analysis\\161024_C01_domainsV3.2.csv", stringsAsFactors = F))
C01.subfams <- unique(C01.domains[Type == "Subfamily" | Type == "subfamily", 
    ])
PLCPs.ageing <- merge(PLCPs.ageing, C01.subfams[, .(ID = Sequence.Name, subfamily = Name)], 
    by = "ID", all.x = T)
kable(PLCPs.ageing[order(dynamics)])
```

| ID | dynamics | subfamily |
| --- | --- | --- |
| Niben101Scf00712g02010 | down.over.time | XCP |
| Niben101Scf01369g00024 | down.over.time | XCP |
| Niben101Scf02336g00012 | down.over.time | XBCP3 |
| Niben101Scf02575g02010 | down.over.time | RD21 |
| Niben101Scf02853g06021 | down.over.time | CEP |
| Nbv5.1tr6236886 | up.over.time | CTB3 |
| Niben101Scf00973g01002 | up.over.time | RD19 |
| Niben101Scf01445g00016.short.SP | up.over.time | ALP |
| Niben101Scf01701g00013 | up.over.time | RD19 |
| Niben101Scf01701g07007 | up.over.time | XBCP3 |
| Niben101Scf02976g00007 | up.over.time | CTB3 |
| Niben101Scf03867g02046 | up.over.time | CTB3 |
| Niben101Scf04007g01012 | up.over.time | RD21 |
| Niben101Scf08921XLOC\_074842 | up.over.time | SAG12 |
| Niben101Scf10490XLOC\_079122 | up.over.time | XBCP3 |

The upregulated SAG12 is, surprisingly, NbPIP1.

In total, 9.2200733 % of all transcripts are up (more abundant at 5, 7 and 10 dpi than at 2) over time in both Buffer and agroinfiltrated plants, while 9.6686103 % are down. This relatively minor change suggests that not much ageing happens within ten days, which is expected.

# Proteome changes over time

```
### Importing and normalization of MS data
library(data.table)
# load the file from perseus after removal of usual suspects, log2
# transformation and filtering for valid values (in at least 3 samples in
# either Agro (incl p19) or Buffer).
MS <- data.table(read.delim("raw.data\\ACE_0056_curatedDB_flfi.txt", stringsAsFactors = F, 
    comment.char = "#"))
nameconv <- data.table(read.csv("analysis\\At.MS.nameconversion.done.csv", stringsAsFactors = F))
setnames(MS, nameconv$MS.ID, nameconv$My.ID)
rm(nameconv)
Buf <- c("A2B", "B2B", "C2B", "A5B", "B5B", "C5B", "A7B", "B7B", "C7B", "A10B", 
    "B10B", "C10B")
Agro <- c("A2W", "B2W", "C2W", "A5W", "B5W", "C5W", "A7W", "B7W", "C7W", "A10W", 
    "B10W", "C10W", "A2P", "B2P", "C2P", "A5P", "B5P", "C5P", "A7P", "B7P", 
    "C7P", "A10P", "B10P", "C10P")
samples <- c("A2B", "B2B", "C2B", "A5B", "B5B", "C5B", "A7B", "B7B", "C7B", 
    "A10B", "B10B", "C10B", "A2W", "B2W", "C2W", "A5W", "B5W", "C5W", "A7W", 
    "B7W", "C7W", "A10W", "B10W", "C10W", "A2P", "B2P", "C2P", "A5P", "B5P", 
    "C5P", "A7P", "B7P", "C7P", "A10P", "B10P", "C10P")
# only Niben
MS <- MS[(Protein.IDs %like% "Nbv") | (Protein.IDs %like% "Niben") | (Protein.IDs %like% 
    "Nicotiana") | (Protein.IDs %like% "p19"), ]

# normalize by volume
MS.l <- melt(MS, id.vars = c("Protein.IDs", "Majority.protein.IDs"), measure.vars = samples, 
    variable.name = "sample", value.name = "LFQ")
cc <- data.table(read.csv("raw.data\\At.samples.protein.cc.csv", stringsAsFactors = F))
MS.l <- merge(MS.l, cc[, .(sample, log2.v.mean..v.sample..)], by = "sample", 
    all = T)
MS.l[, `:=`(norm.LFQ, LFQ + log2.v.mean..v.sample..)]
MS.l$log2.v.mean..v.sample.. <- NULL

# make the factors I need
MS.l[sample %like% "W" | sample %like% "P", `:=`(treatment, "Agro")]
MS.l[sample %like% "[0-9]B", `:=`(treatment, "Buffer")]
MS.l[, `:=`(dpi, gsub("[A-Z]{1}([0-9]{1,2})[A-Z]", "\\1", sample))]
MS.l[, `:=`(bio.repl, substr(sample, 1, 1))]
# MS.l[ , .N/3, by=c('treatment', 'dpi')] so everything still in here

# get the data I need in long format
MS.l[, `:=`(av.biorepls, mean(norm.LFQ)), by = c("Majority.protein.IDs", "treatment", 
    "dpi")]
MS.lfcs <- merge(MS.l[dpi %in% c("5", "7", "10"), ], unique(MS.l[dpi == "2", 
    .(Protein.IDs, av.2 = av.biorepls, bio.repl, treatment)]), by = c("Protein.IDs", 
    "bio.repl", "treatment"), all.x = T)
MS.lfcs[, `:=`(lfc.time, norm.LFQ - av.2), by = c("Protein.IDs", "treatment")]
MS.lfcs.p <- merge(MS.lfcs, MS.l[dpi == "2", .(Protein.IDs, norm.LFQ.2 = norm.LFQ, 
    bio.repl, treatment)], by = c("Protein.IDs", "bio.repl", "treatment"), all.x = T, 
    allow.cartesian = T)

# do the t-tests & BH correction
MS.lfcs.p[, `:=`(p.time, t.test(norm.LFQ, norm.LFQ.2, alternative = "two.sided")$p.value), 
    by = c("Protein.IDs", "treatment", "dpi")]
MS.lfcs.p[, `:=`(padj.time, p.adjust(p.time, method = "BH")), by = c("treatment", 
    "dpi")]

# name regulatory categories get averaged lfcs (av of bio.repls) and
# adjusted pvals in wide format
MS.lfcs.p[, `:=`(av.lfc.time, mean(lfc.time)), by = c("Protein.IDs", "treatment", 
    "dpi")]
MS.dyn <- dcast(unique(MS.lfcs.p[, .(Protein.IDs, av.lfc.time, padj.time, treatment, 
    dpi)]), Protein.IDs ~ treatment + dpi, value.var = c("av.lfc.time", "padj.time"))

#'age.up' means all 5, 7 and 10 have to be > 2 dpi!
MS.dyn[av.lfc.time_Agro_5 > 1 & padj.time_Agro_5 < 0.05 & av.lfc.time_Agro_7 > 
    1 & padj.time_Agro_7 < 0.05 & av.lfc.time_Agro_10 > 1 & padj.time_Agro_10 < 
    0.05, `:=`(dynamics, "age.up")]
MS.dyn[av.lfc.time_Agro_5 < (-1) & padj.time_Agro_5 < 0.05 & av.lfc.time_Agro_7 < 
    (-1) & padj.time_Agro_7 < 0.05 & av.lfc.time_Agro_10 < (-1) & padj.time_Agro_10 < 
    0.05, `:=`(dynamics, "age.down")]

library(knitr)
MS.dyn[, .(length(unique(Protein.IDs))), by = "dynamics"]
```

### Annotation and pfam family enrichment analysis

```
#file to get the protein annotation from
MS.dyn.annot <- data.table(read.csv("analysis\\MS.curatedDB.dyn.annot.csv", stringsAsFactors = F))

MS.dyn <- merge(MS.dyn, MS.dyn.annot[ , .(Protein.IDs, MEROPS.family, pfam.No, DE)],
                by="Protein.IDs", all.x=T)

#Pfam enrichment
pfams.all <- MS.dyn[, .(pfam.No=unlist(strsplit(pfam.No, ";")), dynamics), by="Protein.IDs"]
pfams.all.c <- pfams.all[ , .(N.all = .N), by="pfam.No"]#get all pfams in proteome and how often they are represented

#Count how often each pfam turns up in the up or downregulated things over time and whether that's significant enrichment
pfamsAgeUp.c <- pfams.all[dynamics=="age.up", .N, by="pfam.No"]
pfamsAgeUp.c <- merge(pfamsAgeUp.c, pfams.all.c[ , .(N.all, pfam.No)], by="pfam.No", all.x=T)
pfamsAgeUp.c[, p.overrep.AgeUp :=
             phyper(N,#see help->phyper. We are testing whether finding this pfam N or more times is significantly different than what you would expect if 2dpiup were a random subset of the txome.
                    N.all,
                    sum(pfams.all.c$N)-N.all,
                    sum(pfamsAgeUp.c$N), lower.tail = F),
           by="pfam.No"]

pfamsAgeDown.c <- pfams.all[dynamics=="age.down", .N, by="pfam.No"]
pfamsAgeDown.c <- merge(pfamsAgeDown.c, pfams.all.c[ , .(N.all, pfam.No)], by="pfam.No", all.x=T)
pfamsAgeDown.c[, p.overrep.AgeDown :=
             phyper(N,#see help->phyper. We are testing whether finding this pfam N or more times is significantly different than what you would expect if 2dpiDown were a random subset of the txome.
                    N.all,
                    sum(pfams.all.c$N)-N.all,
                    sum(pfamsAgeDown.c$N), lower.tail = F),
           by="pfam.No"]


#Collect the results of how often each pfam turns up in each dynamic category and whether that's significant enrichment
pfams.all.c <- merge(pfams.all.c, pfamsAgeUp.c[ , .(pfam.No, p.overrep.AgeUp, N.AgeUp=N)], by="pfam.No", all.x=T)
pfams.all.c <- merge(pfams.all.c, pfamsAgeDown.c[ , .(pfam.No, p.overrep.AgeDown, N.AgeDown=N)],
                     by="pfam.No", all.x=T)

#Get into long format to be able to summarize properly
pfams.all.c.m1 <- melt(pfams.all.c, id.vars = "pfam.No",
                      measure.vars = c("N.AgeUp", "N.AgeDown"),
                      variable.name = "dynamics", "N.in.category")
pfams.all.c.m2 <- melt(pfams.all.c, id.vars = "pfam.No",
                      measure.vars = c("p.overrep.AgeUp", "p.overrep.AgeDown"),
                      variable.name = "dynamics", "p.overrep")
pfams.all.c.m <- merge(pfams.all.c.m1[ , .(pfam.No,
                                           dynamics=gsub("N.", "", as.character(dynamics)),
                                           N.in.category)],
                       pfams.all.c.m2[ , .(pfam.No,
                                           dynamics=gsub("p.overrep.", "", as.character(dynamics)),
                                           p.overrep)],
                       by=c("pfam.No", "dynamics"))

#Do BH correction of pvals, correcting for the number of tests done per pfam.No
pfams.all.c.m[ , p.overrep.adj := p.adjust(p.overrep, method = "BH"), by="pfam.No"]
pfams.all.c.m.signif <- pfams.all.c.m[p.overrep.adj<0.05, ]#Only look @significantly enriched pfams

#annotate the enriched pfam.Nos with DE
#read and re-format the explanations of pfam IDs, I'm using Pfam 30.0
pfam.explained <- read.delim("raw.data\\Pfam-A.hmm.dat", strip.white = T, stringsAsFactors = F, header = F)
pfam.explained.cols <- data.frame(ID=c(1:16306))
pfam.explained.cols[ ,"ID"] <- substr(pfam.explained[grep(" ID ", pfam.explained$V1), "V1"], 9, 25)
pfam.explained.cols[ ,"AC"] <- substr(pfam.explained[grep(" AC ", pfam.explained$V1), "V1"], 9, 17)
pfam.explained.cols[ ,"DE"] <- substr(pfam.explained[grep(" DE ", pfam.explained$V1), "V1"], 9, 90)
pfam.explained.cols[ ,"TP"] <- substr(pfam.explained[grep(" TP ", pfam.explained$V1), "V1"], 9, 21)
pfam.explained.cols <- data.table(pfam.explained.cols, key="AC")
setnames(pfam.explained.cols, "AC", "pfam.No")
setnames(pfam.explained.cols, "ID", "pfam.ID")
pfam.explained.cols$pfam.No <- trimws(pfam.explained.cols$pfam.No, which = "both")
pfam.explained <- data.table(pfam.explained.cols)
rm(pfam.explained.cols)

#annotate and write out all significantly enriched pfams to look at
pfams.all.c.m.signif.annot <- merge(pfams.all.c.m.signif,
                                    pfam.explained[, .(pfam.No, DE)], by="pfam.No", all.x = T)
write.csv(pfams.all.c.m.signif.annot, "analysis\\pfams.prot.overtime.csv", row.names = F)#This is Table S15
```

So the extracellular proteins upregulated in ageing plants contain an overrepresentation of GH17 (degrading callose in stomata), PR1 and TaXI. The ones downregulated in ageing plants are enriched for actin & tubulin, indicating there is in general less leakage at later time points. This could be due to decreased cytosolic production of these building blocks, but definitely argues in favour of our AF being consistently clean. Interestingly, peroxidases are enriched in both the groups going up and down over time.

What’s up with those PLCPs that were regulated over time on transcript levels?

```
library(knitr)
kable(MS.dyn[MEROPS.family %like% "C01" & !(is.na(dynamics)), .(Protein.IDs, 
    dynamics)])
```

| Protein.IDs | dynamics |
| --- | --- |
| Niben101Scf02159g01012;Niben101Scf02853g06021 | age.down |
| Niben101Scf10490XLOC\_079122 | age.up |

```
kable(MS.dyn[!(is.na(MEROPS.family)) & !(is.na(dynamics)), .(Protein.IDs, MEROPS.family, 
    dynamics)])
```

| Protein.IDs | MEROPS.family | dynamics |
| --- | --- | --- |
| Niben101Scf00294g00014 | I13 | age.up |
| Niben101Scf00640g04023;Niben101Scf05133g06002;Niben101Scf15594XLOC\_086074 | S33 | age.up |
| Niben101Scf01372g11012;Niben101Scf05962XLOC\_062255;Niben101Scf06205XLOC\_063483 | A01 | age.down |
| Niben101Scf02159g01012;Niben101Scf02853g06021 | C01 | age.down |
| Niben101Scf03830g00012 | A01 | age.down |
| Niben101Scf03963g00006 | A01 | age.up |
| Niben101Scf04060g03003;Niben101Ctg12839g00001 | A01 | age.up |
| Niben101Scf05890XLOC\_061782 | S10 | age.up |
| Niben101Scf05948XLOC\_062102;Niben101Scf07261XLOC\_068442 | M38 | age.up |
| Niben101Scf06424XLOC\_064533 | I03 | age.up |
| Niben101Scf10490XLOC\_079122 | C01 | age.up |
