# Supplementary material for: The transcriptome, extracellular proteome and active secretome of agroinfiltrated Nicotiana benthamiana uncover a large, diverse protease repertoire
Source: Plant Biotechnol J. 2017 Dec 17;16(5):1068–84. doi: 10.1111/pbi.12852 (PMC5902771; doi:10.1111/pbi.12852)
Supplement: Supplementary file 24 — Appendix S5 R code used for ABPP‐MS data analysis [file PBI-16-1068-s020.html]

Bubbles figure for activity level


# Bubbles figure for activity level

#### *FGH*

#### *6 April 2017*

I will make a (day 5 only) bubbles figure for the activity level. This is File S10.

## Which proteins are pulled down on FP in Buf at 5 dpi, which in Agro?

```
library(data.table)
# load the file from perseus after removal of usual suspects, log2
# transformation and filtering for valid values .
MS <- data.table(read.delim("raw.data\\ACE_0245_separate\\ACE_0245_1-6_13-16_proteinGroups_flfi.txt", 
    stringsAsFactors = F, comment.char = "#"))
MS <- MS[Protein.IDs %like% "Niben" | Protein.IDs %like% "Nbv5" | Protein.IDs %like% 
    "Nicotiana"]
nameconv <- data.table(read.csv("analysis\\nameconv_FP.csv", stringsAsFactors = F))
setnames(MS, nameconv$MS_ID, nameconv$my_ID)
pulldown <- nameconv[!(my_ID %like% "now"), my_ID]

MS.long <- melt(MS, id.vars = c("Protein.IDs", "Majority.protein.IDs"), measure.vars = nameconv$my_ID, 
    variable.name = "sample", value.name = "LFQ")

MS.long[sample %like% "FP", `:=`(probe, "FP")]
MS.long[sample %like% "NPC", `:=`(probe, "NPC")]
MS.long[sample %like% "Agro", `:=`(treatment, "Agro")]
MS.long[sample %like% "Buf", `:=`(treatment, "Buffer")]
MS.long[sample %like% "NPC", `:=`(treatment, "mix")]  #consider the NPCs in bulk

library(stringi)
```

```
## Warning: package 'stringi' was built under R version 3.2.5
```

```
MS.long[, `:=`(bio.repl, stri_sub(sample, -1, -1))]
MS.long[sample == "NPC_Buf", `:=`(bio.repl, "C")]
MS.long[sample == "NPC_mix", `:=`(bio.repl, "D")]
MS.long[, `:=`(av.LFQ, mean(LFQ)), by = c("Protein.IDs", "Majority.protein.IDs", 
    "treatment")]

# test for significant enrichment in FP_Agro
MS.Agro <- merge(MS.long[treatment == "Agro", .(Protein.IDs, LFQ.Agro = LFQ, 
    bio.repl)], unique(MS.long[probe == "NPC", .(Protein.IDs, av.LFQ.NPC = av.LFQ, 
    LFQ.NPC = LFQ, bio.repl)]), by = c("Protein.IDs", "bio.repl"))
MS.Agro[, `:=`(p.FP.Agro, t.test(LFQ.Agro, LFQ.NPC, alternative = "two.sided")$p.value), 
    by = "Protein.IDs"]
MS.Agro[, `:=`(padj.FP.Agro, p.adjust(na.omit(p.FP.Agro), method = "BH"))]

# test for significant enrichment in FP_Buffer
MS.Buf <- merge(MS.long[treatment == "Buffer", .(Protein.IDs, LFQ.Buf = LFQ, 
    bio.repl)], unique(MS.long[probe == "NPC", .(Protein.IDs, av.LFQ.NPC = av.LFQ, 
    LFQ.NPC = LFQ, bio.repl)]), by = c("Protein.IDs", "bio.repl"))
MS.Buf[, `:=`(p.FP.Buf, t.test(LFQ.Buf, LFQ.NPC, alternative = "two.sided")$p.value), 
    by = "Protein.IDs"]
MS.Buf[, `:=`(padj.FP.Buf, p.adjust(na.omit(p.FP.Buf), method = "BH"))]

# Merge the results and print numbers
MS.res <- merge(MS.Agro, MS.Buf, by = c("Protein.IDs", "bio.repl", "av.LFQ.NPC", 
    "LFQ.NPC"))
MS.res[, `:=`(av.LFQ.Buf, mean(LFQ.Buf)), by = "Protein.IDs"]
MS.res[, `:=`(av.LFQ.Agro, mean(LFQ.Agro)), by = "Protein.IDs"]
MS.res[, `:=`(lfc.AvsB.FP, LFQ.Agro - av.LFQ.Buf), by = "Protein.IDs"]
MS.res[, `:=`(av.lfc.AvsB.FP, mean(lfc.AvsB.FP)), by = "Protein.IDs"]
MS.res[padj.FP.Agro < 0.1 & padj.FP.Buf < 0.1, `:=`(p.AvsB.FP, t.test(LFQ.Agro, 
    LFQ.Buf, alternative = "two.sided")$p.value), by = "Protein.IDs"]
MS.res[padj.FP.Agro < 0.1 & padj.FP.Buf < 0.1, `:=`(padj.AvsB.FP, p.adjust(na.omit(p.AvsB.FP), 
    method = "BH"))]
MS.res[padj.FP.Agro < 0.1 & !(padj.FP.Buf < 0.1), `:=`(dynamics.act, "active only in agroinfiltrated")]
MS.res[!(padj.FP.Agro < 0.1) & padj.FP.Buf < 0.1, `:=`(dynamics.act, "active only in control")]
MS.res[!(padj.FP.Agro < 0.1) & !(padj.FP.Buf < 0.1), `:=`(dynamics.act, "contaminant")]
MS.res[padj.FP.Agro < 0.1 & padj.FP.Buf < 0.1 & padj.AvsB.FP < 0.05 & av.lfc.AvsB.FP > 
    0, `:=`(dynamics.act, "activity up in agroinfiltrated")]
MS.res[padj.FP.Agro < 0.1 & padj.FP.Buf < 0.1 & padj.AvsB.FP < 0.05 & av.lfc.AvsB.FP < 
    0, `:=`(dynamics.act, "activity down in agroinfiltrated")]
MS.res[padj.FP.Agro < 0.1 & padj.FP.Buf < 0.1 & !(padj.AvsB.FP < 0.05 & abs(av.lfc.AvsB.FP) > 
    0), `:=`(dynamics.act, "active in both treatments")]
MS.res[, length(unique(Protein.IDs)), by = "dynamics.act"]
MS.res.FP <- MS.res
rm(MS, MS.Agro, MS.Buf, MS.long, MS.res, nameconv, pulldown)
write.csv(MS.res.FP, "analysis\\pulldownresults.FP.separateMQ.singlebiorepls.csv", 
    row.names = F)
```

## Which proteins are pulled down on DCG04 in Buf at 5 dpi, which in Agro?

```
library(data.table)
# load the file from perseus after removal of usual suspects, log2
# transformation and filtering for valid values .
MS <- data.table(read.delim("raw.data\\ACE_0245_separate\\ACE_0245_7-12_13-16_proteinGroups_flfi.txt", 
    stringsAsFactors = F, comment.char = "#"))
MS <- MS[Protein.IDs %like% "Niben" | Protein.IDs %like% "Nbv5" | Protein.IDs %like% 
    "Nicotiana"]
nameconv <- data.table(read.csv("analysis\\nameconv_DCG04.csv", stringsAsFactors = F))
setnames(MS, nameconv$MS_ID, nameconv$my_ID)
pulldown <- nameconv[!(my_ID %like% "now"), my_ID]

MS.long <- melt(MS, id.vars = c("Protein.IDs", "Majority.protein.IDs"), measure.vars = nameconv$my_ID, 
    variable.name = "sample", value.name = "LFQ")

MS.long[sample %like% "DCG04", `:=`(probe, "DCG04")]
MS.long[sample %like% "NPC", `:=`(probe, "NPC")]
MS.long[sample %like% "Agro", `:=`(treatment, "Agro")]
MS.long[sample %like% "Buf", `:=`(treatment, "Buffer")]
MS.long[sample %like% "NPC", `:=`(treatment, "mix")]  #consider the NPCs in bulk

library(stringi)
MS.long[, `:=`(bio.repl, stri_sub(sample, -1, -1))]
MS.long[sample == "NPC_Buf", `:=`(bio.repl, "C")]
MS.long[sample == "NPC_mix", `:=`(bio.repl, "D")]
MS.long[, `:=`(av.LFQ, mean(LFQ)), by = c("Protein.IDs", "Majority.protein.IDs", 
    "treatment")]

# test for significant enrichment in DCG04_Agro
MS.Agro <- merge(MS.long[treatment == "Agro", .(Protein.IDs, LFQ.Agro = LFQ, 
    bio.repl)], unique(MS.long[probe == "NPC", .(Protein.IDs, av.LFQ.NPC = av.LFQ, 
    LFQ.NPC = LFQ, bio.repl)]), by = c("Protein.IDs", "bio.repl"))
MS.Agro[, `:=`(p.DCG04.Agro, t.test(LFQ.Agro, LFQ.NPC, alternative = "two.sided")$p.value), 
    by = "Protein.IDs"]
MS.Agro[, `:=`(padj.DCG04.Agro, p.adjust(na.omit(p.DCG04.Agro), method = "BH"))]

# test for significant enrichment in DCG04_Buffer
MS.Buf <- merge(MS.long[treatment == "Buffer", .(Protein.IDs, LFQ.Buf = LFQ, 
    bio.repl)], unique(MS.long[probe == "NPC", .(Protein.IDs, av.LFQ.NPC = av.LFQ, 
    LFQ.NPC = LFQ, bio.repl)]), by = c("Protein.IDs", "bio.repl"))
MS.Buf[, `:=`(p.DCG04.Buf, t.test(LFQ.Buf, LFQ.NPC, alternative = "two.sided")$p.value), 
    by = "Protein.IDs"]
MS.Buf[, `:=`(padj.DCG04.Buf, p.adjust(na.omit(p.DCG04.Buf), method = "BH"))]

# Merge the results and print numbers
MS.res <- merge(MS.Agro, MS.Buf, by = c("Protein.IDs", "bio.repl", "av.LFQ.NPC", 
    "LFQ.NPC"))
MS.res[, `:=`(av.LFQ.Buf, mean(LFQ.Buf)), by = "Protein.IDs"]
MS.res[, `:=`(av.LFQ.Agro, mean(LFQ.Agro)), by = "Protein.IDs"]
MS.res[, `:=`(lfc.AvsB.DCG04, LFQ.Agro - av.LFQ.Buf), by = "Protein.IDs"]
MS.res[, `:=`(av.lfc.AvsB.DCG04, mean(lfc.AvsB.DCG04)), by = "Protein.IDs"]
MS.res[padj.DCG04.Agro < 0.1 & padj.DCG04.Buf < 0.1, `:=`(p.AvsB.DCG04, t.test(LFQ.Agro, 
    LFQ.Buf, alternative = "two.sided")$p.value), by = "Protein.IDs"]
MS.res[padj.DCG04.Agro < 0.1 & padj.DCG04.Buf < 0.1, `:=`(padj.AvsB.DCG04, p.adjust(na.omit(p.AvsB.DCG04), 
    method = "BH"))]
MS.res[padj.DCG04.Agro < 0.1 & !(padj.DCG04.Buf < 0.1), `:=`(dynamics.act, "active only in agroinfiltrated")]
MS.res[!(padj.DCG04.Agro < 0.1) & padj.DCG04.Buf < 0.1, `:=`(dynamics.act, "active only in control")]
MS.res[!(padj.DCG04.Agro < 0.1) & !(padj.DCG04.Buf < 0.1), `:=`(dynamics.act, 
    "contaminant")]
MS.res[padj.DCG04.Agro < 0.1 & padj.DCG04.Buf < 0.1 & padj.AvsB.DCG04 < 0.05 & 
    av.lfc.AvsB.DCG04 > 0, `:=`(dynamics.act, "activity up in agroinfiltrated")]
MS.res[padj.DCG04.Agro < 0.1 & padj.DCG04.Buf < 0.1 & padj.AvsB.DCG04 < 0.05 & 
    av.lfc.AvsB.DCG04 < 0, `:=`(dynamics.act, "activity down in agroinfiltrated")]
MS.res[padj.DCG04.Agro < 0.1 & padj.DCG04.Buf < 0.1 & !(padj.AvsB.DCG04 < 0.05 & 
    abs(av.lfc.AvsB.DCG04) > 0), `:=`(dynamics.act, "active in both treatments")]
MS.res[, length(unique(Protein.IDs)), by = "dynamics.act"]
MS.res.DCG04 <- MS.res
rm(MS, MS.Agro, MS.Buf, MS.long, MS.res, nameconv, pulldown)
write.csv(MS.res.DCG04, "analysis\\pulldownresults.DCG04.separateMQ.singlebiorepls.csv", 
    row.names = F)
```

## What are the proteins we pull down annotated as?

```
#collect the pulldown results
##############################
MS.res <- merge(unique(MS.res.FP[ , .(Protein.IDs, av.LFQ.NPC.FP=av.LFQ.NPC,
                               av.LFQ.Buf.FP=av.LFQ.Buf, av.LFQ.Agro.FP=av.LFQ.Agro,
                               padj.AvsB.FP, av.lfc.AvsB.FP,
                               dynamics.act.FP=dynamics.act)]),
                unique(MS.res.DCG04[ , .(Protein.IDs, av.LFQ.NPC.DCG04=av.LFQ.NPC,
                               av.LFQ.Buf.DCG04=av.LFQ.Buf, av.LFQ.Agro.DCG04=av.LFQ.Agro,
                               padj.AvsB.DCG04, av.lfc.AvsB.DCG04,
                               dynamics.act.DCG04=dynamics.act)]),
                by="Protein.IDs", all=T)

#annotate targets
#################
DS.targets <- data.table(read.csv("raw.data\\DS.target_FGH.csv",#I removed the Xylanase inhbitors from her "PI" class
                                  stringsAsFactors = F, strip.white = T))
DS.targets[pfam.No == "PF05922", Target := "SH"]  #classify all I09 as SH
DS.targets <- unique(DS.targets[Target %in% c("SH", "PLCP", "PI"), .(pfam.No, Target)])
#now this only has unique pfams and only the targets of the probes I used

#annotate all proteins
#########################
annot <- data.table(read.csv("raw.data\\annot.curated.csv", stringsAsFactors = F))

MS.IDs <- MS.res[ , .(ID=unlist(strsplit(Protein.IDs, ";"))),
              by=c("Protein.IDs")]
MS.IDs <- merge(MS.IDs, annot[ , .(ID, pfam.No)], by="ID", all.x = T)
MS.IDs <- MS.IDs[ , .(pfam.No.l = unlist(strsplit(pfam.No, ";"))),
                  by=c("Protein.IDs", "ID")]
setnames(DS.targets, "pfam.No", "pfam.No.l")
MS.IDs <- merge(MS.IDs, DS.targets, by="pfam.No.l", all.x = T)
MS.IDs <- merge(MS.IDs, annot, by="ID", all.x = T)

IDs.MS.annot <- MS.IDs[ , lapply(.SD, paste, collapse=";"), by="Protein.IDs"]
IDs.MS.annot.s <- IDs.MS.annot[ , lapply(.SD, function(x) gsub(";NA", "", x)), by="Protein.IDs"]
IDs.MS.annot.s <- IDs.MS.annot.s[ , lapply(.SD, function(x) gsub("NA;", "", x)), by="Protein.IDs"]

dedup <- function(col.name){
  vapply(lapply(strsplit(col.name, ";"), unique), paste, character(1L), collapse = ";")
}

IDs.MS.annot.s[ , MEROPS.family := dedup(MEROPS.family)]
IDs.MS.annot.s[ , ppase.cattype := dedup(ppase.cattype)]
IDs.MS.annot.s[ , MEROPS.subfamily := dedup(MEROPS.subfamily)]
IDs.MS.annot.s[ , CAZY.family := dedup(CAZY.family)]
IDs.MS.annot.s[ , signalP := dedup(signalP)]
IDs.MS.annot.s[ , pfam.No := dedup(pfam.No)]
IDs.MS.annot.s[ , pfam.No.l := dedup(pfam.No.l)]
IDs.MS.annot.s[ , DE := dedup(DE)]
IDs.MS.annot.s[ , Target := dedup(Target)]
IDs.MS.annot.s <- IDs.MS.annot.s[ , lapply(.SD, function(x) gsub("^NA$", NA, x)), by="ID"]

IDs.MS.annot.s[MEROPS.family %like% "I09;S08", MEROPS.family := "S08"]
IDs.MS.annot.s[ppase.cattype %like% "I;S", ppase.cattype := "S"]

MS.IDs <- IDs.MS.annot.s
rm(IDs.MS.annot.s, annot)

MS.res.annot <- merge(MS.res, MS.IDs, by="Protein.IDs")
MS.res.annot[ , dynamics.act.FP:= factor(dynamics.act.FP)]
MS.res.annot[ , dynamics.act.DCG04:= factor(dynamics.act.DCG04)]
MS.res.annot$ID <- NULL
MS.res.annot$pfam.No.l <- NULL
write.csv(MS.res.annot, "analysis\\pulldown.separateMQ.res.annot.csv", row.names = F)
```

## put the data together for bubbles figure

```
#put the data together for bubbles figure
library(data.table)
MS.res.annot <- data.table(read.csv("analysis\\pulldown.separateMQ.res.annot.csv", stringsAsFactors = F))
combi.pulldowns <- unique(MS.res.annot[
  (!(is.na(dynamics.act.FP)) | !(is.na(dynamics.act.DCG04))) & !(is.na(Target)),
    .(dynamics.act.FP, dynamics.act.DCG04, MEROPS.family, pfam.No, DE), by="Protein.IDs"])
combi.pulldowns[ , dynamics.act := dynamics.act.FP]
combi.pulldowns[is.na(dynamics.act.FP), dynamics.act := dynamics.act.DCG04]

triangledata <- combi.pulldowns[!(dynamics.act=="contaminant"), .N, by="dynamics.act"]
triangledata[ , time := 5]
triangledata[dynamics.act %like% "both", time := 7]
triangledata[dynamics.act %like% "agro", direction := 1]
triangledata[dynamics.act %like% "control", direction := -1]
triangledata[dynamics.act %like% "both", direction := 0]

library(ggplot2)
```

```
## Warning: package 'ggplot2' was built under R version 3.2.5
```

```
t <- ggplot(triangledata, aes(x=time, y=direction))
t +
  geom_point(data=triangledata[!is.na(direction),],
             aes(size=N, colour=as.character(direction)))+
  scale_size(range=c(5, 35),
    "number of\nactive proteins",
                     guide=F) +
  scale_x_continuous(limits=c(3, 10), breaks=c(5, 7)) +
  scale_y_continuous(limits=c(-5, 5), breaks=c(-1, 1), labels=c("down", "up"))+
  theme_bw() +
  scale_color_manual(values = c("#d35400", "#5499c7", "#52be80"),
                     labels=c("down", "constant"), "direction\nof change",
                     guide=F) +
  geom_text(data = triangledata[!is.na(direction)],
            aes(label=N), #size=3,
            check_overlap = T) +
  labs(x="days post agroinfiltration", y="apoplast activity level")
```

```
#make table S8
suppleight <- unique(MS.res.annot[
  (!(is.na(dynamics.act.FP)) | !(is.na(dynamics.act.DCG04))),
    .(dynamics.act.FP, dynamics.act.DCG04, Target, MEROPS.family, pfam.No, DE), by="Protein.IDs"])
write.csv(suppleight, "analysis\\pulldowns.S08.csv", row.names = F)
```
