# Supplementary material for: The transcriptome, extracellular proteome and active secretome of agroinfiltrated Nicotiana benthamiana uncover a large, diverse protease repertoire
Source: Plant Biotechnol J. 2017 Dec 17;16(5):1068–84. doi: 10.1111/pbi.12852 (PMC5902771; doi:10.1111/pbi.12852)
Supplement: Supplementary file 25 — Appendix S6 R code used for analysis of discrepancies between changes in extracellular activity, extracellular protein abundance and transcript abundance [file PBI-16-1068-s021.html]

Which proteases show a differential response in activity vs abundance upon agroinfiltration?


# Which proteases show a differential response in activity vs abundance upon agroinfiltration?

#### *FGH*

#### *4 April 2017*

I use the data from separate MQ runs for ACE\_0245, an in-solution digest performed on the input of the ABPP pulldown samples. I omit the Agro proteins, as I showed previously that they are not pulled down.

## For AEP/EP compare the pulldowns to the ISD from ACE\_0245

```
library(data.table)
#Get the pulldown data for both probes and make a table with Protein.IDs.pulldown, Majority.protein.IDs.pulldown, dynamics.activity, Target, MEROPS.family, pfam.No (use "analysis\\pulldownresults.FP.separateMQ.singlebiorepls.csv" & "analysis\\pulldownresults.DCG04.separateMQ.singlebiorepls.csv" made in FileS10_pulldown_analysis.Rmd)
pulldown.FP <- data.table(read.csv("analysis\\pulldownresults.FP.separateMQ.singlebiorepls.csv",
                                   stringsAsFactors = F))
FP.IDs <- data.table(read.delim(
  "raw.data\\ACE_0245_separate\\ACE_0245_1-6_13-16_proteinGroups_flfi.txt",
  stringsAsFactors = F, comment.char = "#"))
pulldown.FP <- merge(pulldown.FP, FP.IDs[ , .(Protein.IDs,
                                        Majority.protein.IDs.pulldown.FP=Majority.protein.IDs)],
                  by="Protein.IDs", all.x = T)
pulldown.FP <- pulldown.FP[dynamics.act %like% "active", .(Protein.IDs.pulldown.FP=Protein.IDs, bio.repl,
                                lfc.AvsB.FP, padj.AvsB.FP)]
rm(FP.IDs)

pulldown.DCG04 <- data.table(read.csv("analysis\\pulldownresults.DCG04.separateMQ.singlebiorepls.csv",
                                   stringsAsFactors = F))
DCG04.IDs <- data.table(read.delim(
  "raw.data\\ACE_0245_separate\\ACE_0245_1-6_13-16_proteinGroups_flfi.txt",
  stringsAsFactors = F, comment.char = "#"))
pulldown.DCG04 <- merge(pulldown.DCG04, DCG04.IDs[ , .(Protein.IDs,
                                        Majority.protein.IDs.pulldown.DCG04=Majority.protein.IDs)],
                  by="Protein.IDs", all.x = T)
pulldown.DCG04 <- pulldown.DCG04[dynamics.act %like% "active",
                                 .(Protein.IDs.pulldown.DCG04=Protein.IDs, bio.repl,
                                   lfc.AvsB.DCG04, padj.AvsB.DCG04)]
rm(DCG04.IDs)

#Put both pulldowns into one table
setnames(pulldown.FP, c("Protein.IDs.pulldown.FP", "lfc.AvsB.FP", "padj.AvsB.FP"),
         c("Protein.IDs.pulldown", "lfc.AvsB", "padj.AvsB"))
setnames(pulldown.DCG04, c("Protein.IDs.pulldown.DCG04", "lfc.AvsB.DCG04", "padj.AvsB.DCG04"),
         c("Protein.IDs.pulldown", "lfc.AvsB", "padj.AvsB"))
pulldown <- rbind(pulldown.DCG04, pulldown.FP)
rm(pulldown.DCG04, pulldown.FP)


#Get the protein abundance data for the proteins I pull down from the ISD in ACE_0245
#  Get the reproducibles (Protein.IDs.pulldown=Protein.IDs.new=Protein.IDs.old) from  "analysis\\ISD.ACE_0245.separateMQ.lfcs.singlebiorepls.csv" made in analysisStrategy4Separate.Rmd
ISD.new.lfcs <- data.table(read.csv("analysis\\ISD.ACE_0245.separateMQ.lfcs.singlebiorepls.csv",
                                    stringsAsFactors = F))
setnames(ISD.new.lfcs, c("lfc.AvsB", "padj.AvsB"), c("lfc.AvsB.new", "padj.AvsB.new"))

#get the reproducibles
pulldown.repr <- merge(pulldown[ , .(Protein.IDs.pulldown, bio.repl)],
                          ISD.new.lfcs[ , .(Protein.IDs.pulldown=Protein.IDs, bio.repl,
                                             lfc.AvsB.new, padj.AvsB.new)],
                       by=c("Protein.IDs.pulldown", "bio.repl"))

#  + For all Protein.IDs.pulldown that are not covered by this, split them into IDs
IDs.pulldown.notrepr <- unique(pulldown[
  !(Protein.IDs.pulldown %in% pulldown.repr$Protein.IDs.pulldown),
  .(ID=unlist(strsplit(Protein.IDs.pulldown, ";"))), by=c("Protein.IDs.pulldown", "bio.repl")])

#  + Split the ISD into IDs, make means for each Protein.IDs.pulldown for each ISD using na.omit=T - thus, the values get taken into account if they are there and if there is an NA in the final table, that means the protein wasn't found in that ISD. 
ISD.new.lfcs.l <- ISD.new.lfcs[ , .(ID=unlist(strsplit(Protein.IDs, ";")), lfc.AvsB.new, padj.AvsB.new),
                                by=c("Protein.IDs", "bio.repl")]

#need to have bio repls (all A-C) in this table pre-merge, otherwise the ones that are still NA after the first round don't get anything from the second round.
IDs.pulldown.notrepr <- merge(IDs.pulldown.notrepr, ISD.new.lfcs.l, by=c("ID", "bio.repl"), all.x = T)
IDs.pulldown.notrepr <- unique(IDs.pulldown.notrepr[ , .(
  lfc.AvsB.new=mean(lfc.AvsB.new, na.rm = T), padj.AvsB.new=mean(padj.AvsB.new, na.rm = T)),
  by=c("Protein.IDs.pulldown", "bio.repl")])
IDs.pulldown.notrepr <- IDs.pulldown.notrepr[!is.na(bio.repl)]#to get rid of Proteins that were never found

#  + Rbind the reproducibles and the non-reproducibles.
pulldown.rnr <- unique(rbind(pulldown.repr, IDs.pulldown.notrepr))

#these look good, so
pulldown <- merge(pulldown, pulldown.rnr, by=c("Protein.IDs.pulldown", "bio.repl"))
rm(IDs.pulldown.notrepr, ISD.new.lfcs.l,
   ISD.new.lfcs, pulldown.rnr, pulldown.repr)
setnames(pulldown,
         c("lfc.AvsB.new", "padj.AvsB.new"), c("lfc.AvsB.ACE_0245", "padj.AvsB.ACE_0245"))

#* Calculate lfc(A)-lfc(P) using the data from ACE_0245
#  + Use the values for the bio.repls that we have now and do the testing in here :)
pulldown[ , mean.lfc.P := mean(lfc.AvsB.ACE_0245), by="Protein.IDs.pulldown"]
pulldown[ , lfcdiff.AP := lfc.AvsB-mean.lfc.P, by=c("Protein.IDs.pulldown", "bio.repl")]
pulldown[!(is.na(lfc.AvsB.ACE_0245)|is.na(lfc.AvsB)),
            p.lfcdiff.AP := t.test(lfc.AvsB.ACE_0245, lfc.AvsB)$p.value,
             by="Protein.IDs.pulldown"]
pulldown[!is.na(p.lfcdiff.AP), padj.lfcdiff.AP := p.adjust(na.omit(p.lfcdiff.AP), method = "BH")]
pulldown[is.na(padj.AvsB), padj.AvsB:=1]
pulldown[is.na(padj.AvsB.ACE_0245), padj.AvsB.ACE_0245 := 1]
pulldown[is.na(padj.lfcdiff.AP), padj.lfcdiff.AP := 1]
pulldown[is.na(padj.lfcdiff.AP), p.lfcdiff.AP := 1]


pulldown.means <- pulldown[ , .(mean.lfc.P.ACE_0245=mean(lfc.AvsB.ACE_0245),
                           mean.padj.P.ACE_0245=mean(padj.AvsB.ACE_0245),
                           mean.lfc.A=mean(lfc.AvsB),
                           mean.padj.A=mean(padj.AvsB),
                           mean.lfcdiff.AP=mean(lfcdiff.AP),
                           mean.p.lfcdiff.AP=mean(p.lfcdiff.AP),
                           mean.padj.lfcdiff.AP=mean(padj.lfcdiff.AP)),
                       by="Protein.IDs.pulldown"]
#This file has 49 IDs, which matches the 47 + 2 that we pull down on FP and DCG04, so this is good.

#annotate the proteins we pull down
###################################
#get annot files
DS.targets <- data.table(read.csv("raw.data\\DS.target_FGH.csv",#I removed the Xylanase inhbitors from her "PI" class
                                  stringsAsFactors = F, strip.white = T))
DS.targets[pfam.No == "PF05922", Target := "SH"]  #classify all I09 as SH
DS.targets <- unique(DS.targets[Target %in% c("SH", "PLCP", "PI"), .(pfam.No, Target)])
#now this only has unique pfams and only the targets of the probes I used

annot <- data.table(read.csv("raw.data\\annot.curated.csv", stringsAsFactors = F))

#annotate FP pulldown
MS.IDs <- pulldown[ , .(ID=unlist(strsplit(Protein.IDs.pulldown, ";"))),
              by=c("Protein.IDs.pulldown")]
MS.IDs <- merge(MS.IDs, annot[ , .(ID, pfam.No)], by="ID", all.x = T)
MS.IDs <- MS.IDs[ , .(pfam.No.l = unlist(strsplit(pfam.No, ";"))),
                  by=c("Protein.IDs.pulldown", "ID")]
setnames(DS.targets, "pfam.No", "pfam.No.l")
MS.IDs <- merge(MS.IDs, DS.targets, by="pfam.No.l", all.x = T)
MS.IDs <- merge(MS.IDs, annot, by="ID", all.x = T)

IDs.MS.annot <- MS.IDs[ , lapply(.SD, paste, collapse=";"), by="Protein.IDs.pulldown"]
IDs.MS.annot.s <- IDs.MS.annot[ , lapply(.SD, function(x) gsub(";NA", "", x)), by="Protein.IDs.pulldown"]
IDs.MS.annot.s <- IDs.MS.annot.s[ , lapply(.SD, function(x) gsub("NA;", "", x)), by="Protein.IDs.pulldown"]

dedup <- function(col.name){
  vapply(lapply(strsplit(col.name, ";"), unique), paste, character(1L), collapse = ";")
}

IDs.MS.annot.s[ , MEROPS.family := dedup(MEROPS.family)]
IDs.MS.annot.s[ , ppase.cattype := dedup(ppase.cattype)]
IDs.MS.annot.s[ , MEROPS.subfamily := dedup(MEROPS.subfamily)]
IDs.MS.annot.s[ , CAZY.family := dedup(CAZY.family)]
IDs.MS.annot.s[ , signalP := dedup(signalP)]
IDs.MS.annot.s[ , pfam.No := dedup(pfam.No)]
IDs.MS.annot.s[ , pfam.No.l := dedup(pfam.No.l)]
IDs.MS.annot.s[ , DE := dedup(DE)]
IDs.MS.annot.s[ , Target := dedup(Target)]
IDs.MS.annot.s <- IDs.MS.annot.s[ , lapply(.SD, function(x) gsub("^NA$", NA, x)), by="ID"]

IDs.MS.annot.s[MEROPS.family %like% "I09;S08", MEROPS.family := "S08"]
IDs.MS.annot.s[ppase.cattype %like% "I;S", ppase.cattype := "S"]

MS.IDs <- IDs.MS.annot.s
rm(IDs.MS.annot.s)

pulldown.means <- merge(pulldown.means, MS.IDs, by="Protein.IDs.pulldown")

#Using the code at the end of the document, I figured out that the p-value to use here is the non-BH corrected one. Previously, I always did BH correction by Protein.IDs, here that means (only one time point) no BH correction.
#count the targets in the categories lfcdiff >/<0
pulldown.means[ , posttranslational := "none"]
pulldown.means[mean.lfcdiff.AP<0 & mean.padj.lfcdiff.AP<0.1, posttranslational := "down"]
pulldown.means[mean.lfcdiff.AP>0 & mean.padj.lfcdiff.AP<0.1, posttranslational := "up"]
pulldown.means[is.na(mean.lfc.P.ACE_0245), posttranslational := "up"]

posttranslat <- pulldown.means[!(is.na(Target)), .N, by="posttranslational"]
write.csv(posttranslat, "analysis\\posttranslat.csv", row.names = F)
#a file to find out what to put next to the pie and for supplement
write.csv(unique(pulldown.means[!(is.na(Target)), .(
  Protein.IDs.pulldown, signalP, MEROPS.family, pfam.No, DE,
  mean.lfc.P.ACE_0245, mean.padj.P.ACE_0245, mean.lfc.A, mean.padj.A,
  mean.lfcdiff.AP, mean.p.lfcdiff.AP, posttranslational)]),
  "analysis\\all.posttranslational.csv", row.names = F)

rm(annot, DS.targets, IDs.MS.annot, MS.IDs, pulldown.means, pulldown, posttranslat)
```

## For EP/T (compare ACE\_0056 and the RNAseq)

```
library(data.table)
#get the ISD data
ISD <- data.table(read.csv("analysis\\ISD5dpi.ACE_0056.lfcs.singlebiorepls.csv", stringsAsFactors=F))#made in analysisStrategy4Separate.Rmd
setnames(ISD, c("lfc.AvsB", "padj.AvsB"), c("lfc.AvsB.ACE_0056", "padj.AvsB.ACE_0056"))
ISD <- ISD[ , .(Protein.IDs, bio.repl, lfc.AvsB.ACE_0056, padj.AvsB.ACE_0056)]
IDs.ISD <- ISD[ , .(ID=unlist(strsplit(Protein.IDs, ";"))), by=c("Protein.IDs", "bio.repl")]

#Get the transcript data for the proteins I pull down: Collapse protein groups (Protein.IDs.pulldown) by averaging the lfcs over the members, taking the transcript-level lfcs from "apoplast.storyboard\\analysis\\Tr.res.all.singlebiorepls.csv" made in lfcPvslfcT.Rmd.
#Everything up to this point _has_ to be done in the full transcriptome dataset, else the dispersion factors etc would be different from what we do to analyze the transcript level response! This is how I got protein and transcript together for my PvsT analyses.
Transcripts <- data.table(read.csv("raw.data\\Tr.res.all.singlebiorepls.csv", stringsAsFactors = F))
Transcripts <- Transcripts[timepoint=="5dpi"]
setnames(Transcripts, "biorepl", "bio.repl")

#get transcript data for ISD
Transcripts.ISD <- merge(IDs.ISD, Transcripts, by=c("ID", "bio.repl"))
Transcripts.ISD[ , lfc.transcript := mean(log2FoldChange), by=c("Protein.IDs", "bio.repl")]#Now get padj in here: take the mean if there is one.
Transcripts.ISD[, padj.transcript := mean(padj, na.rm = T),
               by=c("Protein.IDs", "bio.repl")]
ISD <- merge(ISD,
             unique(Transcripts.ISD[ , .(lfc.transcript, padj.transcript), by=c("Protein.IDs", "bio.repl")]),
             by=c("Protein.IDs", "bio.repl"), all.x = T)
ISD[(is.na(padj.transcript)), padj.transcript := 1]#make sure these don't turn up as significant in excel, which takes an empty cell to be <0.05!!
rm(IDs.ISD, Transcripts.ISD, Transcripts)

#* Calculate lfc(P)-lfc(T) using the data from ACE_0056
ISD[ , mean.lfc.T := mean(lfc.transcript), by="Protein.IDs"]
ISD[ , lfcdiff.PT := lfc.AvsB.ACE_0056-mean.lfc.T, by=c("Protein.IDs", "bio.repl")]
ISD[!(is.na(lfc.AvsB.ACE_0056)|is.na(lfc.transcript)),
            p.lfcdiff.PT := t.test(lfc.AvsB.ACE_0056, lfc.transcript)$p.value,
             by="Protein.IDs"]
ISD[!is.na(p.lfcdiff.PT), padj.lfcdiff.PT := p.adjust(na.omit(p.lfcdiff.PT), method = "BH")]

#annotate the proteins
######################
#get annot files
DS.targets <- data.table(read.csv("raw.data\\DS.target_FGH.csv",#I removed the Xylanase inhbitors from her "PI" class
                                  stringsAsFactors = F, strip.white = T))
DS.targets[pfam.No == "PF05922", Target := "SH"]  #classify all I09 as SH
DS.targets <- unique(DS.targets[Target %in% c("SH", "PLCP", "PI"), .(pfam.No, Target)])
#now this only has unique pfams and only the targets of the probes I used

annot <- data.table(read.csv("raw.data\\annot.curated.csv", stringsAsFactors = F))

#annotate ISD
MS.IDs <- ISD[ , .(ID=unlist(strsplit(Protein.IDs, ";"))),
              by=c("Protein.IDs")]
MS.IDs <- merge(MS.IDs, annot[ , .(ID, pfam.No)], by="ID", all.x = T)
MS.IDs <- MS.IDs[ , .(pfam.No.l = unlist(strsplit(pfam.No, ";"))),
                  by=c("Protein.IDs", "ID")]
setnames(DS.targets, "pfam.No", "pfam.No.l")
MS.IDs <- merge(MS.IDs, DS.targets, by="pfam.No.l", all.x = T)
MS.IDs <- merge(MS.IDs, annot, by="ID", all.x = T)

IDs.MS.annot <- MS.IDs[ , lapply(.SD, paste, collapse=";"), by="Protein.IDs"]
IDs.MS.annot.s <- IDs.MS.annot[ , lapply(.SD, function(x) gsub(";NA", "", x)), by="Protein.IDs"]
IDs.MS.annot.s <- IDs.MS.annot.s[ , lapply(.SD, function(x) gsub("NA;", "", x)), by="Protein.IDs"]

dedup <- function(col.name){
  vapply(lapply(strsplit(col.name, ";"), unique), paste, character(1L), collapse = ";")
}

IDs.MS.annot.s[ , MEROPS.family := dedup(MEROPS.family)]
IDs.MS.annot.s[ , ppase.cattype := dedup(ppase.cattype)]
IDs.MS.annot.s[ , MEROPS.subfamily := dedup(MEROPS.subfamily)]
IDs.MS.annot.s[ , CAZY.family := dedup(CAZY.family)]
IDs.MS.annot.s[ , signalP := dedup(signalP)]
IDs.MS.annot.s[ , pfam.No := dedup(pfam.No)]
IDs.MS.annot.s[ , pfam.No.l := dedup(pfam.No.l)]
IDs.MS.annot.s[ , DE := dedup(DE)]
IDs.MS.annot.s[ , Target := dedup(Target)]
IDs.MS.annot.s <- IDs.MS.annot.s[ , lapply(.SD, function(x) gsub("^NA$", NA, x)), by="ID"]

IDs.MS.annot.s[MEROPS.family %like% "I09;S08", MEROPS.family := "S08"]
IDs.MS.annot.s[ppase.cattype %like% "I;S", ppase.cattype := "S"]

MS.IDs <- IDs.MS.annot.s
rm(IDs.MS.annot.s)

ISD <- merge(ISD, MS.IDs, by="Protein.IDs")
rm(annot, DS.targets, IDs.MS.annot, MS.IDs)

ISD[ , mean.lfcdiff.PT := mean(lfcdiff.PT), by="Protein.IDs"]

#Using the code at the end of the document, I figured out that the p-value to use here is the non-BH corrected one. Previously, I always did BH correction by Protein.IDs, here that means (only one time point) no BH correction.
#count the targets in the categories lfcdiff >/<0
ISD[ , posttranscriptional := "none"]
ISD[mean.lfcdiff.PT<0 & padj.lfcdiff.PT<0.1, posttranscriptional := "down"]
ISD[mean.lfcdiff.PT>0 & padj.lfcdiff.PT<0.1, posttranscriptional := "up"]

posttranscriptional <- ISD[!(is.na(lfcdiff.PT)), length(unique(Protein.IDs)), by="posttranscriptional"]
write.csv(posttranscriptional, "analysis\\posttranscriptional.csv", row.names = F)

#a file to find out what to put next to the pie and for the supplement
write.csv(unique(ISD[ , .(
  signalP, MEROPS.family, pfam.No, DE,
  mean.lfc.T, mean.lfc.P.ACE_0056=mean(lfc.AvsB.ACE_0056),
  mean.lfcdiff.PT, p.lfcdiff.PT, posttranscriptional),
  by="Protein.IDs"]),
  "analysis\\all.posttranscriptional.csv", row.names = F)
```
