# Supplementary material for: The transcriptome, extracellular proteome and active secretome of agroinfiltrated Nicotiana benthamiana uncover a large, diverse protease repertoire
Source: Plant Biotechnol J. 2017 Dec 17;16(5):1068–84. doi: 10.1111/pbi.12852 (PMC5902771; doi:10.1111/pbi.12852)
Supplement: Supplementary file 26 — Appendix S7 Supplemental methods used for mass spectrometry sample preparation [file PBI-16-1068-s022.docx]

# Supplementary methods for mass spectrometry

**Sample Preparation, Reduction/Alkylation and Tryptic Digestion.** In-solution digestion (ISD): Apoplastic fluid corresponding to 3 µg total protein (based on Bradford assay) was placed in a fresh Eppendorf tube and the total volume adjusted to 1 mL with MS-grade water. Proteins were then precipitated by addition of 100 µL trichloroacetic acid (TCA, final ~10%) and incubation on ice for 30 min. The precipitated proteins were collected by centrifugation (18000 g, 10 min, 4°C) and the protein pellet was then washed with 80% Acetone/20% 20 mM ammonium bicarbonate (ABC) solution. The pellet was dried at room temperature (RT) for 5 min and then taken up in 25 µL 8M urea. To help solubilization the samples were sonified in a Branson sonification bath (5 min). Protein reduction and alkylation was achieved by sequential incubation with dithiothreitol (DTT, final 8 mM, 30 min, RT) and iodoacetamide (IAM; final 17 mM, 30 min, RT). After alkylation the DTT concentration was raised to 22 mM in order to quench non-reacted IAM. To start protein digestion under denaturing conditions (urea concentration after reduction/alkylation is still 6.4 M) we added 100 ng LysC (Wako; ratio of 1/30 based on protein concentration). The samples were incubated at 37°C for 3h while gently shaking (600 rpm). The samples were then diluted with 50 mM ABC to a final urea concentration of 1 M. Then 100 ng Sequencing grade Trypsin (Promega; ratio of 1/30 based on protein concentration) was added and the samples incubated at 37 °C over night while gently shaking (600 rpm). The protein digestion was stopped by adding formic acid (FA, final 5% v/v).

On-bead digestion (OBD): for affinity purified proteins immobilized on avidin beads a slightly different approach was chosen. The protein-beads were taken up in 500 µL 8 M urea 50 mM Tris-HCl (pH 8) and reduced with Tris(2-carboxyethyl)phosphine (TCEP; final 10 mM, 15 min, 65 °C, gentle shaking). Alkylation was performed by subsequently adding IAM (final 20 mM, 30 min, 35°C, gentle shaking). LysC-Digestion was started by adding 2 µg of Promega Trypsin-LysC and incubation at 37 °C for three hours while gently shaking. To start the trypsin digestion samples were subsequently diluted with 50 mM Tris to a final urea concentration of 2M and left over night at 37 °C while gently shaking. Next the beads were removed (centrifugation and transfer of digestion mix to fresh Eppendorf tube) and the digestion was stopped by adding triflouracetic acid (TFA) to a final concentration of 1 %.

**Sample clean-up for LC-MS.** Acidified tryptic digests were desalted on home-made C18 StageTips as described (Rappsilber *et al.*, 2007). We used a 2 disc StageTips for each sample and passed the solution over the tips in 150 µL aliquots by centrifugation (600 – 1200 ×g). Bound peptides were washed with 0.1% FA and subsequently eluted with 80% Acetonitrile (ACN). After elution from the StageTips, samples were dried using a vacuum concentrator (Eppendorf) and the peptides were taken up in 10 µL 0.1 % formic acid solution.

**LC-MS/MS**. Experiments were performed on an Orbitrap Elite instrument (Thermo, (Michalski *et al.*, 2012)) that was coupled to an EASY-nLC 1000 liquid chromatography (LC) system (Thermo). The LC was operated in the one-column mode. The analytical column was a fused silica capillary (75 µm × 20 (ISD) or 32 cm(OBD)) with an integrated PicoFrit emitter (New Objective) packed in-house with Reprosil-Pur 120 C18-AQ 1.9 µm resin (Dr. Maisch). The analytical column was encased by a column oven (Sonation) and attached to a nanospray flex ion source (Thermo). The column oven temperature was adjusted to 45 °C during data acquisition. The LC was equipped with two mobile phases: solvent A (0.1% formic acid, FA, in water) and solvent B (0.1% FA in acetonitrile, ACN). All solvents were of UPLC grade (Sigma). Peptides were directly loaded onto the analytical column with a maximum flow rate that would not exceed the set pressure limit of 980 bar (usually around 0.5 – 0.8 µL/min). Peptides were subsequently separated on the analytical column by running a 140-min gradient of solvent A and solvent B at a flow rate of 300 nl/min (gradient ISD: start with 7% B; gradient 7% to 35% B for 120 min; gradient 35% to 100% B for 10 min and 100% B for 10 min) at a flow rate of 300 nl/min. Gradient OBD: start with 7% B; gradient 7% to 35% B for 120 min; gradient 35% to 80% B for 10 min and 80% B for 10 min). The mass spectrometer was operated using Xcalibur software (version 2.2 SP1.48). The mass spectrometer was set in the positive ion mode. Precursor ion scanning was performed in the Orbitrap analyzer (FTMS; Fourier Transform Mass Spectrometry) in the scan range of m/z 300-1800 and at a resolution of 60000 with the internal lock mass option turned on (lock mass was 445.120025 m/z, polysiloxane) (Olsen *et al.*, 2005). Product ion spectra were recorded in a data dependent fashion in the ion trap (ITMS) in a variable scan range and at a rapid scan rate. The ionization potential (spray voltage) was set to 1.8 kV. Peptides were analyzed using a repeating cycle consisting of a full precursor ion scan (1.0 × 106 ions or 50 ms) followed by 15 product ion scans (1.0 × 104 ions or 50 ms) where peptides are isolated based on their intensity in the full survey scan (threshold of 500 counts) for tandem mass spectrum (MS2) generation that permits peptide sequencing and identification. Collision induced dissociation (CID) energy was set to 35% for the generation of MS2 spectra. During MS2 data acquisition dynamic ion exclusion was set to 60 seconds with a maximum list of excluded ions consisting of 500 members and a repeat count of one. Ion injection time prediction, preview mode for the Fourier transform mass spectrometer (FTMS, the orbitrap), monoisotopic precursor selection and charge state screening were enabled. Only charge states higher than 1 were considered for fragmentation.

**Peptide and Protein Identification using MaxQuant**. RAW spectra were submitted to an Andromeda (Cox *et al.*, 2011) search in MaxQuant (version 1.5.3.30) using the default settings (Cox & Mann, 2008). Label-free quantification and match-between-runs was activated (Cox *et al.*, 2014). MS/MS spectra data were searched against our in house *N. benthamiana* (DB05_representative_proteins_curated.fasta; 74091 entries) and the Uniprot *Agrobacterium fabrum* (strain C58) (UP000000813_176299.fasta; 5376 entries) databases. All searches included a contaminants database (as implemented in MaxQuant, 245 sequences) and the p19_vector_proteins.fasta database (3 entries). The contaminants database contains known MS contaminants and was included to estimate the level of contamination, the p19 Database contains all proteins encoded by the p19 vector. Andromeda searches allowed oxidation of methionine residues (16 Da), acetylation of the protein N-terminus (42 Da) and Carbamylation on protein N-terminus and Lysin (K) (43 Da) as dynamic modifications and the static modification of cysteine (57 Da, alkylation with iodoacetamide). Enzyme specificity was set to “Trypsin/P”. The instrument type in Andromeda searches was set to Orbitrap and the precursor mass tolerance was set to ±20 ppm (first search) and ±4.5 ppm (main search). The MS/MS match tolerance was set to ±0.5 Da. The peptide spectrum match FDR and the protein FDR were set to 0.01 (based on target-decoy approach). Minimum peptide length was 7 amino acids. For protein quantification unique and razor peptides were allowed. Modified peptides were allowed for quantification. The minimum score for modified peptides was 40. Further analysis and filtering of the results was done in Perseus v1.5.5.3 (Tyanova *et al.*, 2016).

**ABPP-MS and affinity purification of extracellular Ser hydrolases and PLCPs**: 4 mL of AF were collected from 15 leaves for each sample and adjusted to 50 mM NaAc pH 5.5, 5 mM DTT, consistent with the acidic environment in the extracellular proteome. Labelling of Ser hydrolases with 5 µM FP (Kaschani *et al.*, 2012) was performed for 2h at room temperature with agitation and labelling of PLCPs with 5 µM DCG-04 (Greenbaum *et al.*, 2002) was performed for 4h at room temperature with agitation. The pH was adjusted by adding 0.4 mL Tris pH 8 to each sample and Methanol-Chloroform precipitation was performed to terminate the labelling reaction and remove excess probe. For Methanol-Chloroform precipitation, the sample was mixed with 16 mL cold (-20 °C) MeOH, 4 mL cold (-20 °C) chloroform and 12 mL ice-cold water. After centrifugation for 30 min at 3000 x g and 4 °C, the top layer was discarded, the remaining sample mixed with 16 mL cold (-20 °C) MeOH and centrifuged again for 30 min at 3000 x g and 4 °C. The resulting protein pellet was dried and denatured in 1.2 % SDS-PBS (1.2 % w/v sodium dodecyl sulphate in phosphate buffered saline) for 5 min at 95 °C and samples were diluted to reach a final concentration of SDS < 0.2 %. Labelled proteins were then enriched on avidin-agarose beads (Sigma-Aldrich, St. Louis, US). The beads were washed five times in PBS prior to use, then incubated with the sample for 1 hour at room temperature with agitation. Unspecifically bound proteins were removed by washing four times in 1 % SDS and two times in water. The on-bead immobilized proteins were not eluted but on-bead digested using Trypsin/LysC (Promega, Fitchburg, US) according to the manufacturer’s instructions and peptides were prepared for mass spectrometry.

Cox, J., Hein, M.Y., Luber, C.A., Paron, I., Nagaraj, N., & Mann, M. (2014) Accurate proteome-wide label-free quantification by delayed normalization and maximal peptide ratio extraction, termed MaxLFQ. *Mol. Cell. Proteomics*, **13**, 2513–2526.

Cox, J. & Mann, M. (2008) MaxQuant enables high peptide identification rates, individualized p.p.b.-range mass accuracies and proteome-wide protein quantification. *Nat. Biotechnol.*, **26**, 1367–1372.

Cox, J., Neuhauser, N., Michalski, A., Scheltema, R.A., Olsen, J.V., & Mann, M. (2011) Andromeda: a peptide search engine integrated into the MaxQuant environment. *J. Proteome Res.*, **10**, 1794–1805.

Michalski, A., Damoc, E., Lange, O., Denisov, E., Nolting, D., Muller, M., Viner, R., Schwartz, J., Remes, P., Belford, M., Dunyach, J.-J., Cox, J., Horning, S., Mann, M., & Makarov, A. (2012) Ultra high resolution linear ion trap Orbitrap mass spectrometer (Orbitrap Elite) facilitates top down LC MS/MS and versatile peptide fragmentation modes. *Mol. Cell. Proteomics*, **11**, O111.013698-O111.013698.

Olsen, J.V., de Godoy, L.M., Li, G., Macek, B., Mortensen, P., Pesch, R., Makarov, A., Lange, O., Horning, S., & Mann, M. (2005) Parts per million mass accuracy on an Orbitrap mass spectrometer via lock mass injection into a C-trap. *Mol. Cell. Proteomics*, **4**, 2010–2021.

Rappsilber, J., Mann, M., & Ishihama, Y. (2007) Protocol for micro-purification, enrichment, pre-fractionation and storage of peptides for proteomics using StageTips. *Nat. Protoc.*, **2**, 1896–1906.

Tyanova, S., Temu, T., Sinitcyn, P., Carlson, A., Hein, M.Y., Geiger, T., Mann, M., & Cox, J. (2016) The Perseus computational platform for comprehensive analysis of (prote)omics data. *Nat. Methods*, **13**, 731–740.
